# Dual‐Responsive Phosphorus‐Based Fluorescent Sensors: Synthesis and Selective Metal Sensing of Pyrazolyl Phosphine Oxides

**DOI:** 10.1002/anie.202501421

**Published:** 2025-04-07

**Authors:** Ethan D. E. Calder, Isobel R. J. Hawes, Andrew R. Jupp

**Affiliations:** ^1^ School of Chemistry University of Birmingham Edgbaston Birmingham B15 2TT UK

**Keywords:** Dual‐responsive sensors, Fluorescent metal sensing, Main‐group‐based sensors, Phosphorus‐based sensors, Pyrazolyl phosphine oxides

## Abstract

Despite a wealth of previously reported frameworks for fluorescent metal sensors, there are few examples of phosphorus‐based fluorophores being used in metal sensing applications. Here, we report the synthesis and characterization of a new family of pyrazolyl phosphine oxides and their use in metal sensing applications. The mechanism of their formation has been probed in detail with both computational and experimental studies, rationalizing the selectivity of the reaction. Their use as dual‐responsive fluorescent metal sensors is then demonstrated, with “turn‐off” and “turn‐on” responses observed for Fe^3+^ and Al^3+^, respectively. These systems exhibit good selectivity, large Stokes shifts, and submicromolar limits of detection and will open new avenues in phosphorus‐based fluorophores and metal‐sensing applications.

## Graphical abstract

Pyrazolyl phosphine oxides are shown to act as dual‐responsive fluorescent sensors, displaying a selective “turn‐off” fluorescence response for Fe^3+^ and a “turn‐on” response for Al^3+^. These systems serve as a new framework in the field of sensing materials based on main‐group elements such as phosphorus.

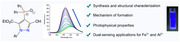

## Introduction

The use of main‐group elements in sensing applications has expanded greatly in recent years. The Lewis acidic character of many main‐group elements has allowed for the development of sensors for an array of substrates, including halide ions,^[^ ^1^, ^2^, ^3^ ^]^ and small molecules such as nitrous oxide and carbon monoxide.^[^ ^4^, ^5^ ^]^ Another key class of substrates for such sensors is metal ions. The sensing of metal ions used in biological processes, such as iron and copper, is vital for medical contexts whereby either an excess or deficiency can cause health issues.^[^ ^6^, ^7^, ^8^ ^]^ The ability to sense metal ions not directly involved in biological processes, such as aluminum, also retains importance due to their toxicity at even low concentrations.^[^ ^9^, ^10^ ^]^ Therefore, sensors that can detect these metals quickly and selectively are of widespread interest. Various analytical methods can be employed to sense such ions, such as mass spectrometry,^[^ ^11^, ^12^ ^]^ atomic absorption spectroscopy,^[^ ^13^ ^]^ or electrochemical techniques.^[^ ^14^, ^15^ ^]^ However, sensing materials based on fluorescence remain particularly popular owing to their high sensitivity, relatively lower cost, and rapid analysis.^[^ ^16^ ^]^ This has allowed for the development of a variety of molecular systems able to sense metal ions in solution with fluorescence spectroscopy, via either a “turn‐off” or “turn‐on” response.^[^ ^17^ ^]^

**Figure 1:**
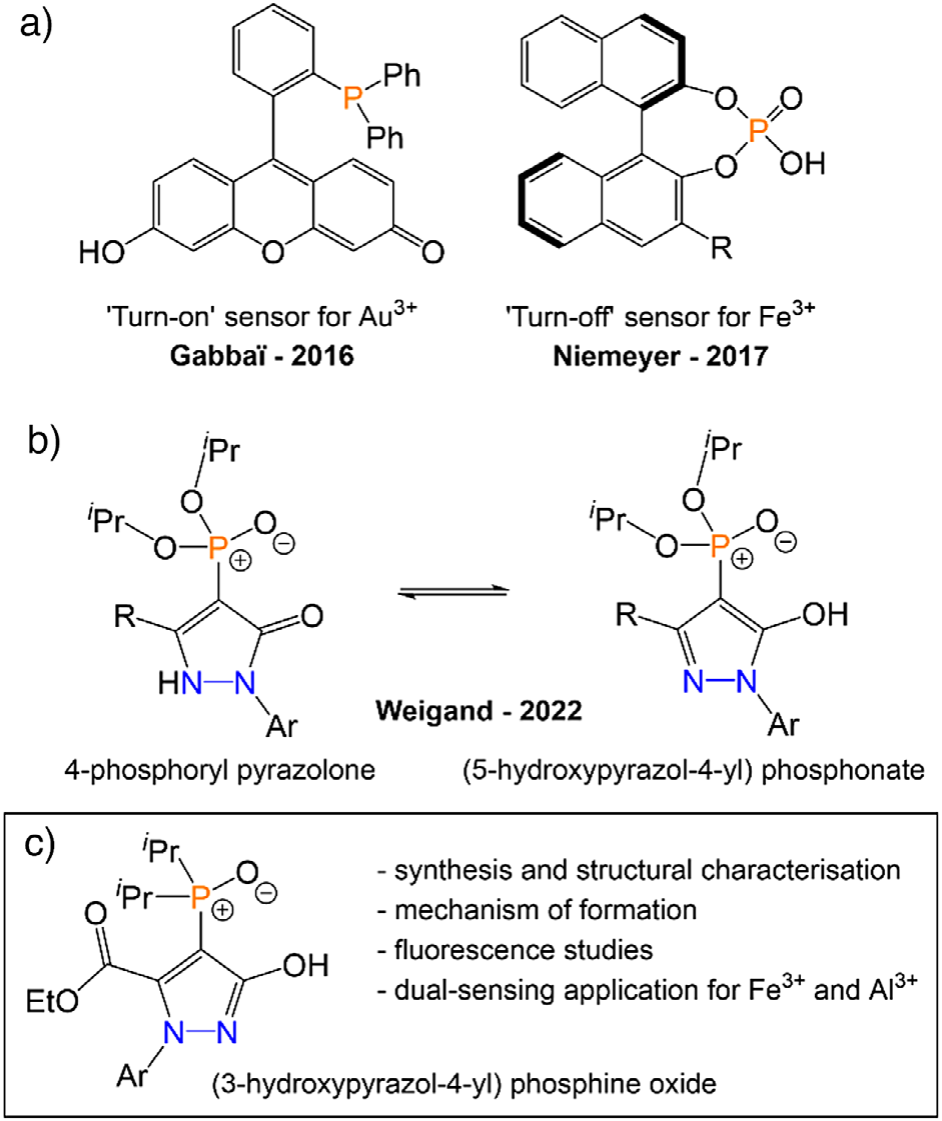
a) Examples of phosphorus‐based fluorescent metal sensors. b) 4‐phosphoryl pyrazolones and the corresponding tautomer previously used in metal binding. c) This work.

While numerous systems have been developed as fluorescent metal sensors, and certain main‐group elements such as sulfur have been incorporated into sensors,^[^ ^18^ ^]^ other main‐group elements such as phosphorus have scarcely been used in such applications. Such systems typically rely on a phosphorus‐based functional group that is responsible for interacting or binding with the metal, taking advantage of the well‐established donor abilities of trivalent phosphines or pentavalent P═O containing moieties, such as phosphine oxides or phosphoric acid derivatives. This is tethered to a fluorescent backbone, allowing for a “turn‐off” or “turn‐on” response in the presence of metal ions (Figure 1a). In 2016, Christianson and Gabbaï reported a phosphine‐decorated fluorescein, in which an arylphosphine was linked to the fluorescein backbone via an *o*‐phenylene group.^[^ ^19^ ^]^ Coordination of the phosphine to Au^3+^ yielded a “turn‐on” fluorescence response and use of the system as a sensor for Au^3+^ in water. In 2020, Gates and co‐workers reported a polymeric phosphine sensor that also exhibited “turn‐on” fluorescence in the presence of Au^+^ or Au^3+^.^[^ ^20^ ^]^ This effect was also attributed to coordination of the P(III) center to the metal ions, in conjunction with a fluorene‐based backbone. Other phosphorus‐based moieties have also been reported in the context of metal sensing: in 2017, Niemeyer and co‐workers developed a BINOL (1,1′‐binaphthyl‐2,2′‐diol) derived bis‐phosphoric acid framework that exhibited a selective “turn‐off” response in the presence of Fe^3+^.^[^ ^21^ ^]^ Other examples based on phosphine sulfides,^[^ ^22^ ^]^ phosphonates,^[^ ^23^ ^]^ and phospholes^[^ ^24^ ^]^ have also been reported. These systems benefit as metal sensors from the donor abilities of the phosphorus moieties, in combination with a robust fluorescent backbone.

Searching for other systems that may serve as phosphorus‐based metal sensors, we explored the possibility of a phosphorus‐functionalized pyrazole unit. Although free pyrazole is not fluorescent, appropriately substituted pyrazoles often display fluorescence, with high quantum yields and large Stokes shifts; these advantages make them particularly useful in metal sensing applications.^[^ ^25^, ^26^ ^]^ Phosphoryl‐substituted pyrazoles and pyrazolines have been widely studied in medicinal applications and in the agrochemical industry as herbicides and insecticides.^[^ ^27^, ^28^ ^]^ A 4‐phosphoryl pyrazolone, bearing a phosphine oxide unit, was reported in 1996, but no further reactivity was explored.^[^ ^29^ ^]^ Tris(pyrazolyl)phosphine oxides featuring P─N bonds have also been studied as scorpionate ligands for metal centers.^[^ ^30^, ^31^, ^32^, ^33^, ^34^ ^]^ Very recently, Wang and co‐workers developed a synthesis of axially chiral pyrazol‐3‐ylphosphine oxides bearing atropisomeric arylpyrazole moieties.^[^ ^35^ ^]^ Furthermore, Weigand and co‐workers have recently reported a range of 4‐phosphoryl pyrazolones.^[^ ^36^, ^37^, ^38^, ^39^ ^]^ These molecules exist in equilibrium between the keto (pyrazolone) and enol (hydroxypyrazole) tautomers; the majority crystallized as the latter and feature an ─OH unit adjacent to the phosphonate group (Figure 1b). This arrangement creates a binding pocket that has been demonstrated to coordinate metals including lanthanides, actinides, alkali metals, and alkaline earth metals. However, no luminescence was reported for any of these molecules.

Despite the variety of phosphorus‐appended pyrazoles discussed above, there are no examples of metal sensing using these systems. In this manuscript, we report the synthesis and characterization of a new range of pyrazolyl phosphine oxides via the reaction of five‐membered phosphorus‐based heterocycles with H_2_O. Our method allows one‐pot access to the unexplored (3‐hydroxy‐1*H*‐pyrazol‐4‐yl)phosphine oxides (Figure 1c), henceforth simplified to pyrazolyl phosphine oxides. The mechanism of this transformation has been probed both computationally and experimentally. The pyrazolyl phosphine oxides are highly fluorescent, which allows these molecules to act as metal sensors. These air‐ and water‐stable sensors are selective for different metals, affording either a “turn‐off” dynamic quenching mechanism for Fe^3+^, or a “turn‐on” binding mechanism for Al^3+^, representing the first example of a phosphorus‐appended pyrazole as a fluorescent metal sensor.

## Results and Discussion

**Scheme 1:**
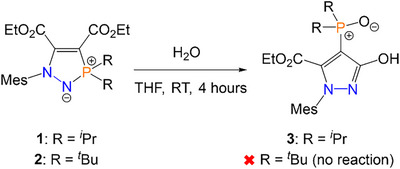
Synthesis of pyrazolyl phosphine oxide **3** from precursor **1**. The structurally related precursor **2** does not react with H_2_O to undergo the same transformation.

We recently reported the synthesis of five‐membered phosphorus/nitrogen‐containing heterocycles **1** and **2** (Scheme 1) via the cycloaddition reactions of azophosphines (MesN_2_PR_2_; Mes = mesityl; R = *^i^* Pr, *^t^* Bu) with diethyl acetylenedicarboxylate ((C(CO_2_Et))_2_).^[^ ^40^ ^]^ Structurally analogous species were also reported by the Tanaka group.^[^ ^41^ ^]^ The addition of water to a THF solution of heterocycle **1** resulted in clean conversion to a new compound, pyrazolyl phosphine oxide **3** (Scheme 1), with a ^31^P NMR chemical shift of 62.3 ppm. This reaction features loss of the initial phosphorus/nitrogen‐based heterocycle and the formation of a hydroxypyrazole core with an exocyclic phosphine oxide moiety. Overall, the transformation of **1** to **3** corresponds to the addition of H_2_O and the loss of EtOH, with the phosphorus center remaining in a P^V^ oxidation state. The reaction can proceed slowly if **1** is simply exposed to air (full conversion within 4 days) but can be carried out in under 4 h if a large excess (100 equiv.) of H_2_O is added directly to the solution of **1**. Although **1** reacts readily with H_2_O to form **3**, it was not observed to react with other O─H or N─H bonds; the reactions of **1** with NH_3_ and various anilines and alcohols yielded no reaction when monitored by ^31^P{^1^H} NMR spectroscopy, even with heating to 60 °C over 2 days (see Section S1.9). Interestingly, the bulkier analogue **2** (R = *^t^* Bu) showed no reactivity toward H_2_O by ^31^P{^1^H} NMR spectroscopy, even with a significant excess of H_2_O (100 equiv.) and heating to reflux in various solvents (see Section S1.8).

**Scheme 2:**
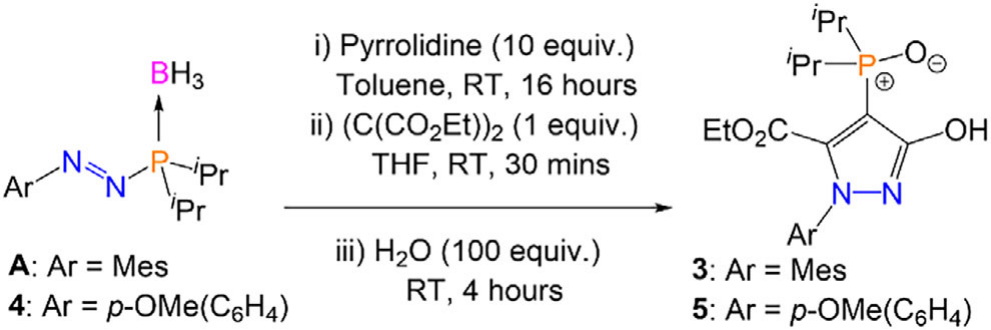
One‐pot synthesis of pyrazolyl phosphine oxides **3** and **5** from azophosphine–boranes.

Although the hydrolysis reaction to generate **3** is very facile, it does require prior synthesis and isolation of heterocycle **1**. This heterocycle is synthesized by the cycloaddition of the activated alkyne to a free azophosphine, the latter of which is produced by the deprotection of the azophosphine–borane MesN_2_P*^i^* Pr_2_.BH_3_ (**A**; see Figure S1 in Section S1.7). To enable simple access to the desired pyrazolyl phosphine oxides, we devised a one‐pot approach directly from the air‐stable precursor **A** (Scheme 2, and in Section S1.7). The one‐pot approach precludes the need to isolate the azophosphine and heterocycle intermediates, which significantly reduces the amount of solvent used and removes the silica required for chromatography. Furthermore, the total yield of **3** via the one‐pot method was higher than the combined yields of the stepwise approach (65% versus 48%).

To assess the effects of a more electron‐donating aryl ring on the properties of the pyrazolyl phosphine oxides, azophosphine–borane *p*‐OMe(C_6_H_4_)N_2_P(*^i^* Pr)_2_.BH_3_ (**4**, Scheme 2) was synthesized according to our general synthetic procedure.^[^ ^42^, ^43^ ^]^ The spectroscopic data for this new compound are consistent with our previously reported azophosphine–boranes, and the single‐crystal X‐ray diffraction (SXRD) structure is shown in Figure S72 (see Supporting Information for details). **4** could be converted to the corresponding pyrazolyl phosphine oxide **5** via the one‐pot procedure in 56% yield (Scheme 2). In the ^1^H NMR spectra of both **3** and **5**, the ─OH protons possess very downfield chemical shifts (both 11.1 ppm), consistent with the *enol* tautomers of the pyrazole unit, rather than the *keto* form. In the IR spectra for both compounds, only one C═O peak (from the ester moiety) is observed for each, also consistent with the *enol* form.

**Figure 2:**
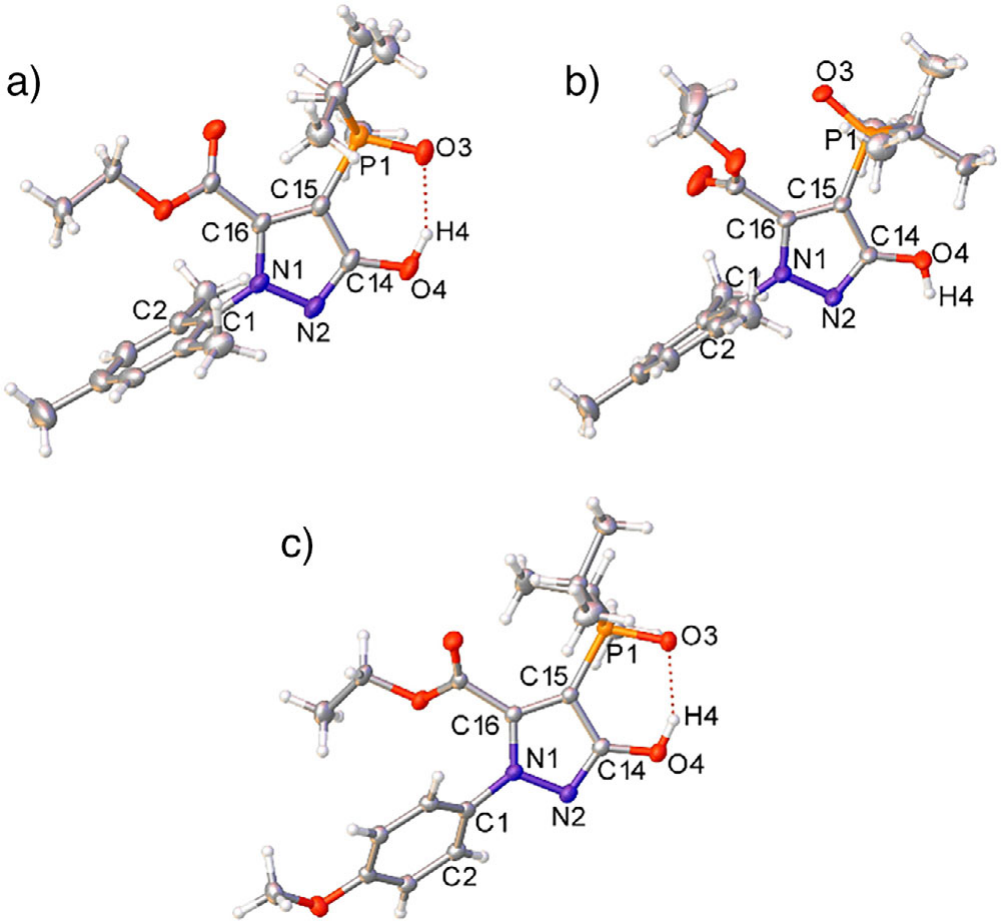
Single crystal structures of **3α** a), **3β** b), and **5** c) with thermal ellipsoids drawn at the 50% probability level. **3β** crystallized with two product molecules in the asymmetric unit with statistically similar bond metrics and one THF (solvent) molecule; only one product molecule is shown here for clarity.^[^ ^44^ ^]^ Selected bond distance (Å) and angles (°): **3α** N1─N2 1.369(3), N2─C14 1.325(4), C14─C15 1.414(4), C15─C16 1.385(4), N1─C16 1.358(4), C14─O4 1.347(4), P1─O3 1.508(2), N2─N1─C1─C2 −89.9(3); **3β** N1─N2 1.375(4), N2─C14 1.323(4), C14─C15 1.430(4), C15─C16 1.386(5), N1─C16 1.346(4), C14─O4 1.331(4), P1─O3 1.504(2), N2─N1─C1─C2 −‍88.9(4). **5** N1─N2 1.369(2), N2─C14 1.326(2), C14─C15 1.422(2), C15─C16 1.391(2), N1─C16 1.355(2), C14─O4 1.345(2), P1─O3 1.5093(13), N2─N1─C1─C2 −55.9(2).

Pyrazolyl phosphine oxide **3** crystallized in two different forms, designated here as **3α** and **3β**. Slow evaporation of a concentrated *n*‐hexane solution of **3** yielded single crystals of **3α** with no solvent of crystallization present in the structure (Figure 2a). The solid‐state structure of **3α** is in its *enol* form, with intramolecular hydrogen bonding between the ─OH unit and the phosphine oxide. The P─O bond length of **3α** is typical of a phosphine oxide [1.508(2) Å], though longer than the P─O bonds reported in the similar phosphonates reported by Zhang et al.^[^ ^37^ ^]^ The N2─C14 [1.325(4) Å] and C14─O4 bond lengths are [1.347(4) Å], indicative of predominantly double‐bond and single‐bond character, respectively. Single crystals of **5** were grown by slow evaporation of a concentrated *n*‐hexane solution of **5**, which are analogous to **3α** and display similar bond metrics (Figure 2c). The most notable difference is the aryl ring of **3α**, which is almost perpendicular to the pyrazole core, whereas the aryl ring of **5** is significantly less so (**3α**: N2─N1─C1─C2 = −89.9(3)°, **5**: N2─N1─C1─C2 = −55.9(2)°). This effect can be attributed to the additional steric hindrance of the *ortho*‐CH_3_ groups in **3α**. When single crystals of **3** were grown from slow evaporation of a THF/hexane mixture (1:5 v/v), **3β** was preferentially formed instead (Figure 2b). There are two molecules of **3β** in the asymmetric unit along with one noncoordinating molecule of THF. The phosphine oxide moiety has rotated around the P1─C15 bond so there is no longer any intramolecular hydrogen bonding, but the crystal packing does enable intermolecular hydrogen bonding between the OH unit of one molecule and the phosphine oxide of another. This gives rise to a coordination network of **3β** molecules, connected via intermolecular H‐bonding interactions (Figure S73). This also highlights that the interaction among O3, H4, and O4 is not so strong as to prevent rotation of the phosphorus functionality.

**Scheme 3:**
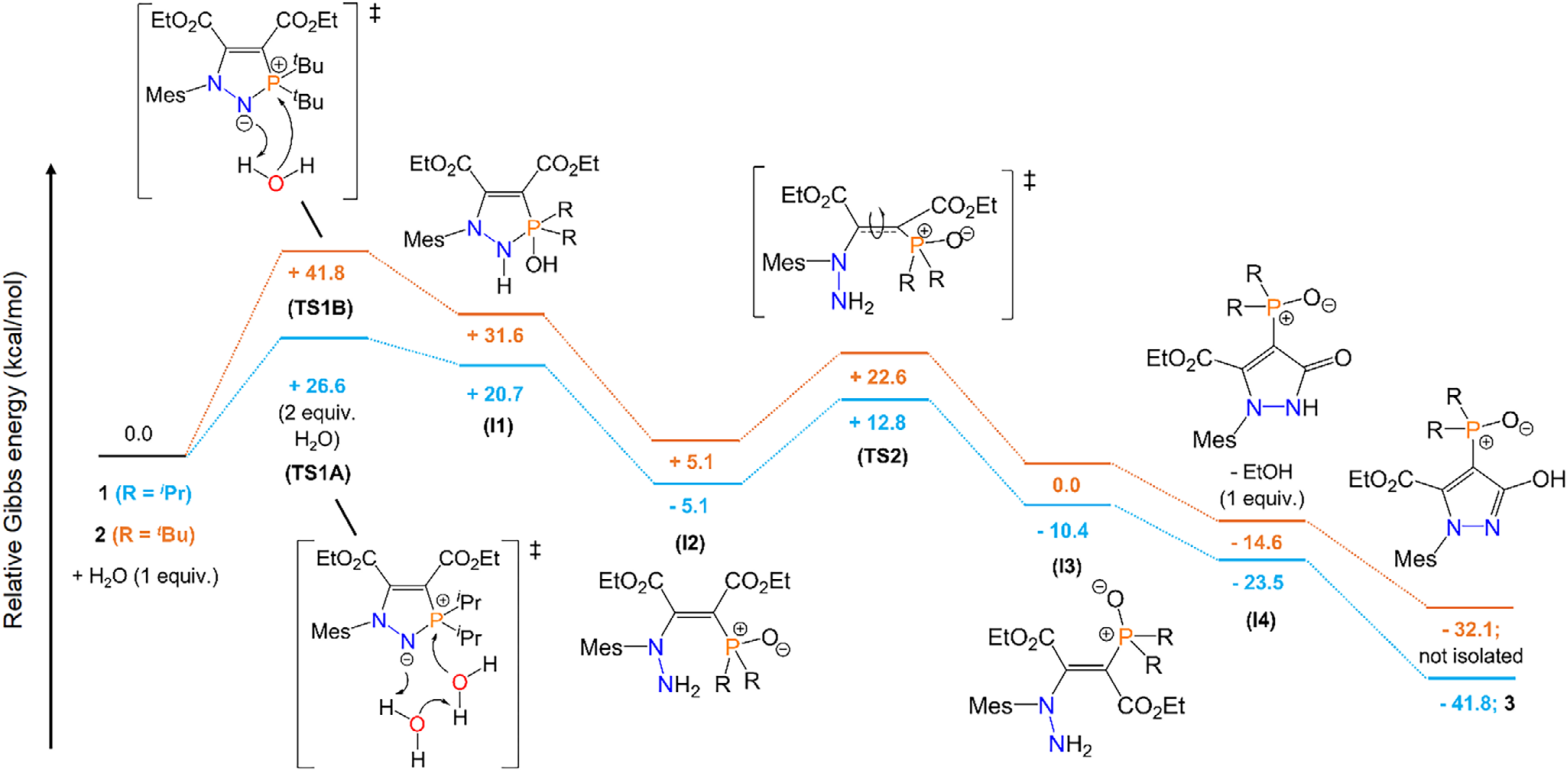
Computed pathways (Gibbs free energy, kcal mol^−1^) for the reactions of **1** and **2** with H_2_O to form the corresponding pyrazolyl phosphine oxides, at the ωB97XD/def2TZVP//ωB97XD(toluene)//def2QZVP level of theory. For full computational details, see Supporting Information.

To determine the minimum energy pathway for the formation of pyrazolyl phosphine oxide **3** from **1**, density functional theory (DFT) calculations were carried out. We also sought to rationalize why the reaction is unique to H_2_O, and why the analogous pyrazolyl phosphine oxide with R = *^t^* Bu substituents did not form. Our calculations showed the addition of H_2_O across the N─P bond to be the first and rate‐limiting step for this reaction (Scheme 3). For **1** (R = *^i^* Pr), the lowest energy pathway occurs via cooperative action of two H_2_O molecules, with a six‐membered transition state (**TS1A**) over a high but accessible barrier of 26.6 kcal mol^−1^. For **2** (R = *^t^* Bu), an analogous six‐membered transition state could not be found, presumably due to the additional steric bulk of the *^t^* Bu groups. Instead, addition of one H_2_O molecule across the N─P bond proceeds over a significantly higher energy barrier of 41.8 kcal mol^−1^ (**TS1B**), which we observed to be experimentally inaccessible even with heating to 120 °C. Heating higher than this led to the decomposition of **2** and an intractable mixture of products (see Section S1.8). Proceeding over **TS1** leads to intermediate **I1**, with N─H and P─OH moieties, before cleavage of the N─P single bond and tautomerism to a phosphine oxide yields intermediate **I2**. Cummins and co‐workers previously reported the reaction of an *N*‐heterocyclic iminophosphorane, structurally similar to **1** and **2**, to also react with H_2_O to give N─P bond cleavage and formation of N─NH_2_ and phosphine oxide functionalities, analogous to **I2**, although no further reactivity was reported.^[^ ^41^ ^]^

**I2** exists initially as the *Z*‐isomer, isomerizing to the thermodynamically favored *E*‐isomer (**I3**) over an accessible energy barrier of 17.9 kcal mol^−1^ for R = *^i^* Pr (**TS2**). The low energy barrier for this C═C isomerism can be attributed to the delocalization of the lone pair of the adjacent nitrogen center into the C═C bond, and further into the ─CO_2_Et and phosphine oxide functionalities.

This was supported by natural bond orbital (NBO) analysis, which showed donation from the nitrogen lone pair into the C═C π* orbital of 65.3 kcal mol^−1^ for **I2** and 67.4 kcal mol^−1^ for **I3** (R = *^i^* Pr). Analysis of the Wiberg bond indices also showed reduced double bond character in the C═C bond (1.56 for **I2**; 1.65 for **I3,** both R = *^i^* Pr) and increased N─C double bond character (1.21 for **I2**; 1.16 for **I3,** both R = *^i^* Pr). Following this isomerism, ring‐closing occurs via attack of the nucleophilic amine onto the carbonyl of the ester group, which are both on the same side of the *E*‐isomer, to form **I4**, with concomitant release of EtOH. **I4** exists as the *keto* form of the product; our calculations show that the *enol* form is clearly favored over the *keto* form (energy difference of 18.3 kcal mol^−1^ for **3**). This preference for the *enol* form is in corroboration with both solid‐state and solution‐phase experimental data.

**Scheme 4:**
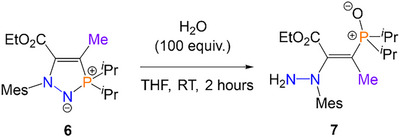
Synthesis of alkene **7** via the reaction of precursor **6** with H_2_O.

**Figure 3:**
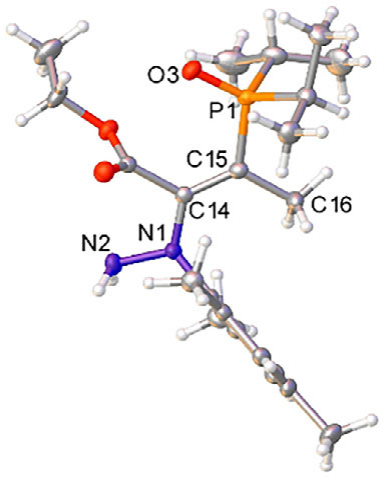
Single crystal structure of **7**, with thermal ellipsoids drawn at the 50% probability level. **7** crystallized with two molecules in the asymmetric unit with statistically similar bond metrics, both the *E*‐isomer; only one is shown here for clarity.^[^ ^44^ ^]^ Selected bond distance (Å): N1─N2 1.430(3), N1─C14 1.380(3), C14─C15 1.362(4), P1─O3 1.495(2).

In addition to the aforementioned DFT studies, we also sought to gain experimental evidence for the proposed reaction pathway. We speculated that the replacement of the reactive ─CO_2_Et functionality on **1** with an unreactive ─CH_3_ group would prevent ring‐closing and thus stop the reaction from proceeding past **I3**. We thus synthesized phosphorus/nitrogen‐containing heterocycle **6** (Scheme 4) in situ, via the reaction of the corresponding azophosphine (MesN_2_P*^i^* Pr_2_) with ethyl 2‐butynoate (H_3_CC≡CCO_2_Et). Addition of an excess (10 equiv.) of H_2_O allowed for conversion to the new product **7** within 2 h (Scheme 4). Slow evaporation of an *n*‐hexane solution yielded single crystals of **7** suitable for SXRD (Figure 3). To our satisfaction, **7** is structurally analogous to the proposed intermediate **I3**, with one ─CO_2_Et replaced by a ─CH_3_ group. **7** exists as the computationally favored *E*‐isomer. In line with the structure proposed by DFT, **7** possesses a longer than idealized C14═C15 bond [1.362(4) Å] and a shorter than idealized N1─C14 bond [1.380(3) Å], reflective of the weakened C═C bond that allows isomerism to occur. In the solution phase, only one set of peaks was observed by multinuclear NMR spectroscopy, supporting the presence of only one isomer in solution. 2D‐NOESY NMR spectroscopy also supported the assignment of **7** as the *E*‐isomer, with a clear cross‐peak between the methyl group on the alkene and the *ortho*‐methyl groups on the mesityl substituent.

**Figure 4:**
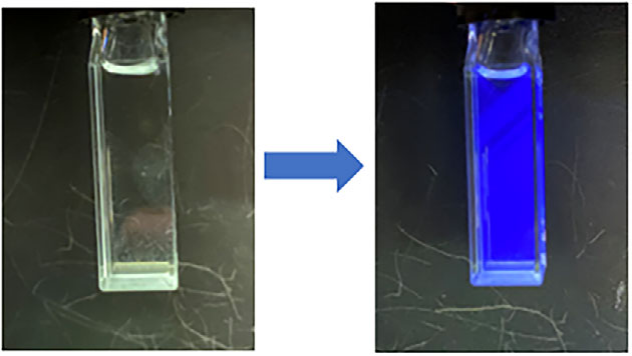
Left side: 5 µM solution of **3** in DCM in visible light. Right side: The same solution irradiated under a short‐wave (*λ*_exc_ = 254 nm) UV lamp.

Given our aim of using pyrazolyl phosphine oxides **3** and **5** in fluorescence applications, we investigated their photophysical properties in solution. Both **3** and **5** display absorption maxima (*λ*_abs_) in the UV region (**3** = 274–287 nm, **5** = 279–298 nm) with molar absorption coefficients of 4000–13 000 M^−1^ cm^−1^ depending on the solvent (Table 1). Both **3** and **5** exhibit fluorescence, displaying blue‐colored emission (Figure 4), with very large Stokes shifts (139–189 nm) across a range of solvents. The emission of pyrazolyl phosphine oxides could, in principle, be modulated further via modifying the *N*‐aryl substituent on the azophosphine precursors, which has previously been shown to affect their photophysical properties.^[^ ^43^ ^]^ The fluorescence intensities and quantum yields of **3** and **5** differ significantly, however, with **3** displaying greater fluorescence intensities and higher quantum yields (Table 1). Choice of solvent also has a significant effect on intensity and quantum yield, with more electron‐donating solvents yielding lower fluorescence intensities and quantum yields. This is presumably due to such donating solvents disrupting the intramolecular interactions between the adjacent P═O and O─H units on the pyrazole unit, which TD‐DFT calculations showed to be crucial in the luminescence of pyrazolyl phosphine oxides (see Section S5.3).

**Table 1:** Photophysical properties of pyrazolyl phosphine oxides **3** and **5** across a range of solvents.

Compound	Solvent	*λ*_abs_ ^a)^ (nm)	*λ*_em_ ^b)^ (nm)	*Φ*_F_ ^c)^
**3**	Toluene	287	426	0.89
**3**	DCM	283	422	0.40
**3**	MeCN	281	423	0.24
**3**	MeCN/H_2_O (1:1)	274	461	0.12
**5**	Toluene	298	462	0.36
**5**	DCM	292	466	0.089
**5**	MeCN	288	470	0.043
**5**	MeCN/H_2_O (1:1)	279	468	0.029

*λ*_abs_: absorbance wavelength, *λ*_exc_: excitation wavelength, *λ*_em_: emission wavelength, *Φ*_F_: fluorescence quantum yield.

^a)^ 50 µM solutions.

^b)^ 5 µM solutions, *λ*_abs_ = *λ*_exc_.

^C)^ 50 µM solutions, *λ*_abs_ = *λ*_exc_.

**Figure 5:**
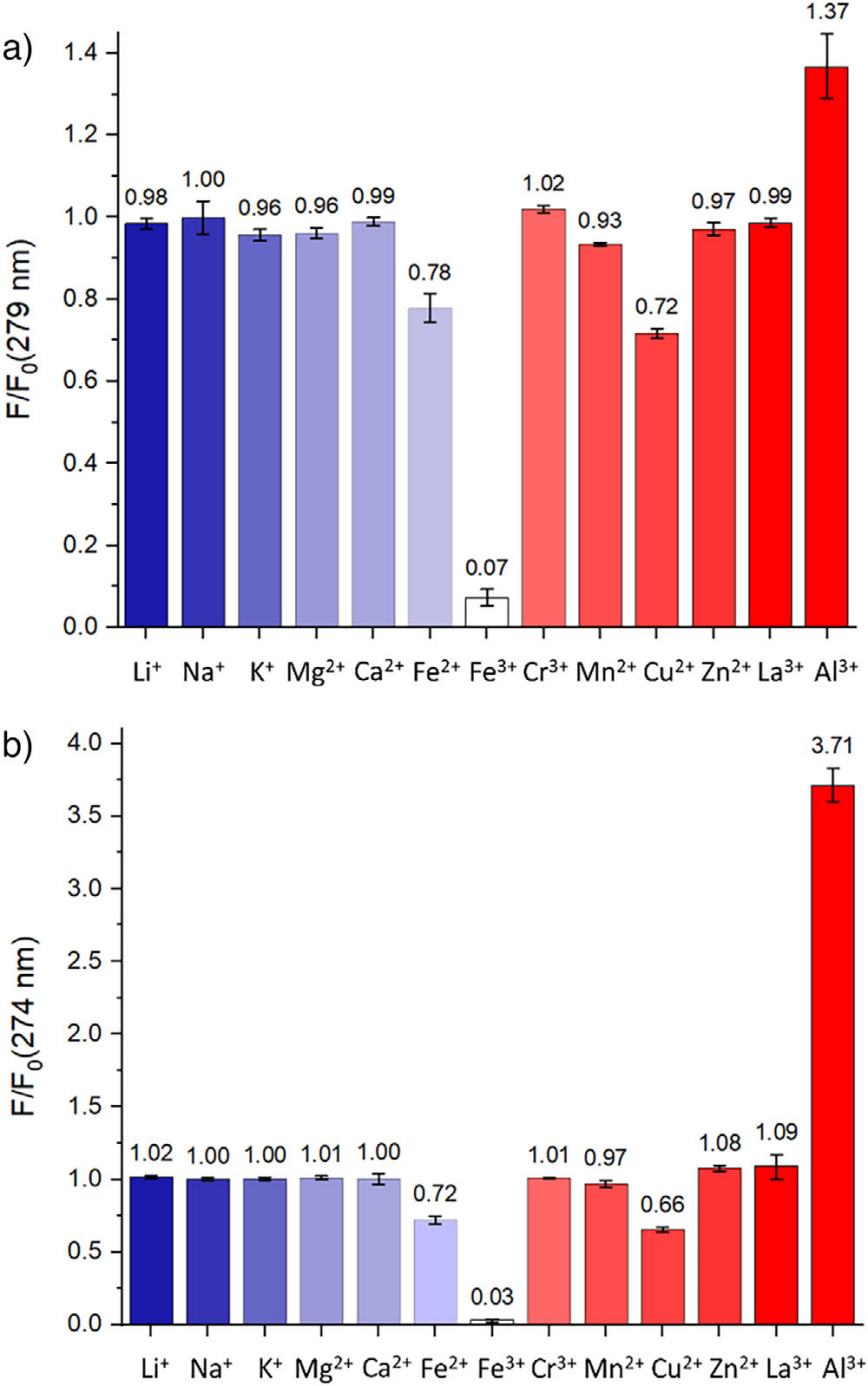
*F*/*F*_0_ ratios for **5** a) and **3** b) (50 µM in 1:1 MeCN/H_2_O) in the presence of different metal ions (5 equiv., 250 µM). All values from triplicate measurements. **5**: *λ*_exc_ = 279 nm, *λ*_em_ = 468 nm. **3**: *λ*_exc_ = 274 nm, *λ*_em_ = 461 nm. Error bars represent the maximum and minimum values obtained from triplicate measurements.

We next evaluated the fluorescence behavior of pyrazolyl phosphine oxides **3** and **5** in the presence of a series of metal ions. We examined the interactions of **3** and **5** with a variety of alkali and alkaline earth metals (Li^+^, Na^+^, K^+^, Mg^2+^, Ca^2+^), transition metals (Cr^3+^, Mn^2+^, Fe^2+^, Fe^3+^, Cu^2+^, Zn^2+^), a main‐group metal (Al^3+^), and a lanthanide metal (La^3+^). Due to the poor solubility of **3** and **5** in water and the poor solubility of many metal salts in organic solvents, a 1:1 MeCN/H_2_O mixture was used as the solvent for all metal‐sensing experiments, as this was found to fully dissolve both the metal salts and **3** and **5**. To obtain an overview of the interactions of **3** and **5** with the metal ions, 5 equivalents of the corresponding metal salt were mixed with 1 equivalent of pyrazolyl phosphine oxide **5**. For the majority of metals (Li^+^, Na^+^, K^+^, Mg^2+^, Ca^2+^, Mn^2+^, Zn^2+^), the fluorescence intensity remained largely unchanged (*F*/*F*_0_ = 0.93–1.00, Figure 5a). However, in the presence of Fe^3+^, the fluorescence intensity becomes almost completely quenched (*F*/*F*_0_ = 0.07). Less pronounced quenching was also observed in the presence of Fe^2+^ (*F*/*F*_0_ = 0.78) and Cu^2+^ (*F*/*F*_0_ = 0.72). Notably, two of the other trivalent ions (Cr^3+^, La^3+^) showed only negligible changes in fluorescence intensity in the presence of **5** (*F*/*F*_0_ = 0.99–1.02). The final trivalent cation, Al^3+^, reproducibly showed a slight increase in intensity (*F*/*F*_0_ = 1.37). These results indicate the possibility of **5** as a selective fluorescence “turn‐off” sensor for Fe^3+^. Due to the ubiquitous nature of Fe^3+^ ions in biological and environmental systems, sensitive techniques for Fe^3+^ sensing remain highly in demand. The apparent selectivity of **5** for Fe^3+^ over Fe^2+^ and Cu^2+^ is particularly valuable, with these ions being the most common competitor ions for Fe^3+^, especially in “turn‐off” fluorescent sensors.^[^ ^6^, ^45^ ^]^

The interactions of pyrazolyl phosphine oxide **3** with most metal ions are very similar to that of **5** (Figure 5b), including Fe^3+^ (*F*/*F*_0_ = 0.03), Fe^2+^ (*F*/*F*_0_ = 0.72), and Cu^2+^ (*F*/*F*_0_ = 0.66). However, the interaction of **3** with Al^3+^ was remarkably more pronounced, with a significant increase in fluorescence intensity observed (*F*/*F*_0_ = 3.71), Despite its toxic nature in many biological systems, fluorescent sensors for Al^3+^ are relatively less common than for other metal cations.^[^ ^9^, ^10^ ^]^ The majority of previously reported examples of fluorescent sensors for Al^3+^ rely on Schiff bases, with Al^3+^ binding to the fluorophore via N and O donor centers and inducing a “turn‐on” response.^[^ ^46^, ^47^, ^48^ ^]^ The significant “turn‐on” response observed here with Al^3+^ is particularly beneficial, as such systems generally provide greater sensitivity and fewer false positives than “turn‐off” fluorescence responses.

**Figure 6:**
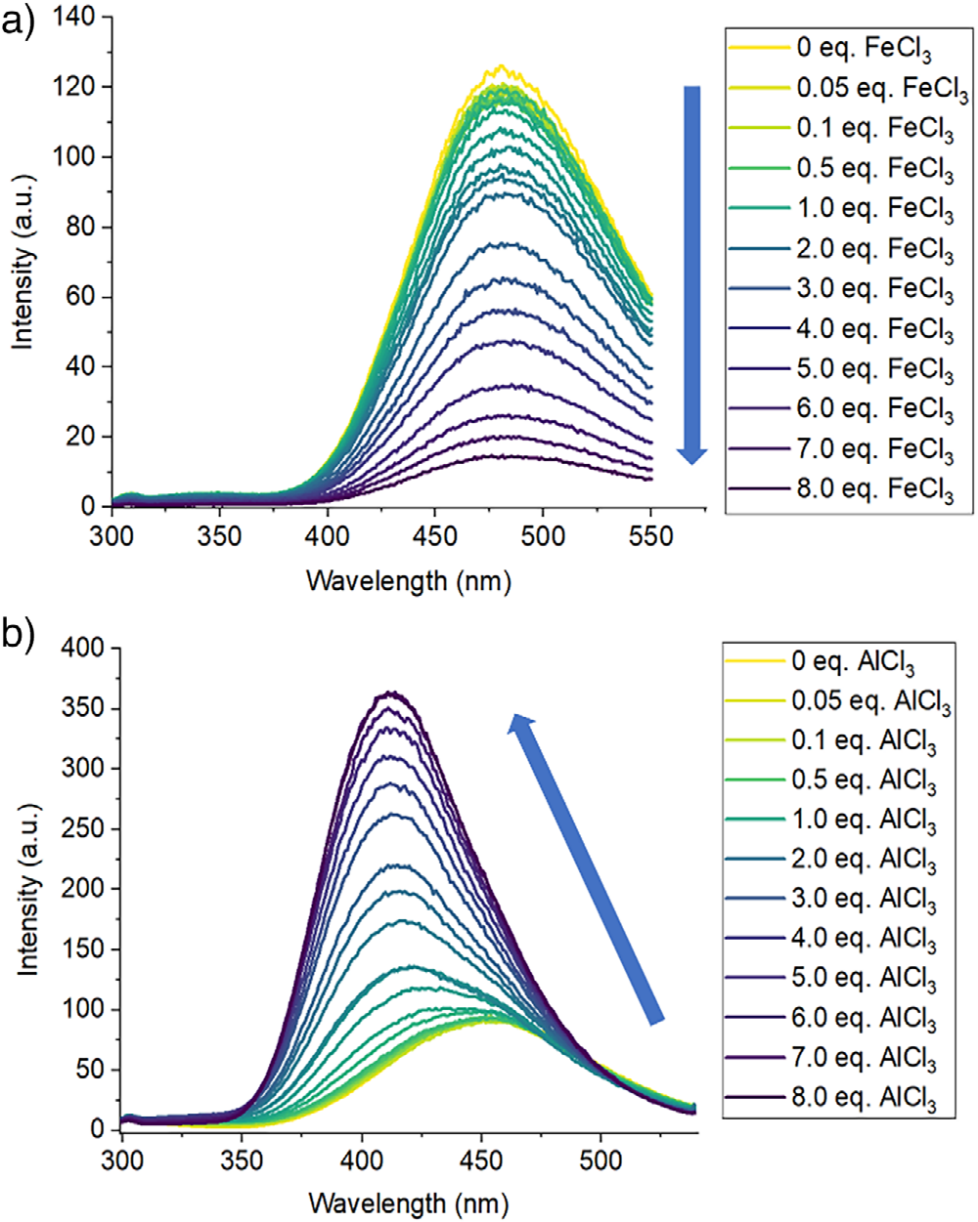
a) Fluorescence spectra for the titration of **5** (50 µM in 1:1 MeCN/H_2_O; *λ*_exc_ = 279 nm) with FeCl_3_ (0–8 equiv.). b) Fluorescence spectra for the titration of **3** (10 µM in 1:1 MeCN/H_2_O; *λ*_exc_ = 274 nm) with AlCl_3_ (0–8 equiv.).

To further investigate the interactions of **3** and **5** with metal ions, fluorescence titrations of metal ions into solutions of **3** and **5** were performed. We first examined the interactions of Fe^3+^ with **3** and **5**. Successive additions of Fe^3+^ (0–8 equiv., over 23 steps) to a solution of **5** yielded a continuous decrease in fluorescence intensity, with the emission wavelength remaining the same. (Figure 6a). When plotted for a fixed emission wavelength, a linear relationship between emission intensity and concentration of Fe^3+^ (see Figure S17) was observed. Stern–Volmer analysis revealed a linear behavior at lower Fe^3+^ concentrations ([Fe^3+^] ≤ 100 µM, *K*_SV_ = 3502 ± 333 M^−1^; see Figure S18), with an upward deviation at higher concentrations. Furthermore, no change in the absorption wavelength was observed via UV–vis spectroscopy upon increasing the concentration of Fe^3+^ (see Figure S19). Similar behavior via both fluorescence and UV–vis spectroscopy was observed upon titration of Fe^3+^ into a solution of **3** (see Supporting Information).

Given the increased fluorescence intensity observed upon the interaction of **3** with Al^3+^, we also titrated Al^3+^ (0–8 equiv., over 23 steps) into a solution of **3**. This behaved differently; a nonlinear increase in fluorescence intensity was observed, with a clear blueshift in emission wavelength at higher concentrations of Al^3+^ (411 nm versus 461 nm; Figure 6b). When plotted for a fixed emission wavelength, a sigmoidal curve shape was observed, in contrast to the linear relationship seen with Fe^3+^ (see Figure S21). This was also accompanied by a clear redshift in the main absorbance band in the UV/vis spectrum for **3** upon increasing concentrations of Al^3+^, from 274 to 296 nm (see Figure S22). These results are consistent with a binding mechanism between **3** and Al^3+^ ions, in which the corresponding **3**/Al^3+^ complex forms at higher Al^3+^ concentrations and displays increased fluorescence intensity compared to **3**, yielding a “turn‐on” response. The nonlinearity observed in this titration may also be partially rationalized by acidification of the unbuffered solvent system used, following increasing addition of AlCl_3_.

To investigate this binding further, we analyzed the stoichiometry of a possible **3**/Al^3+^ complex using Job's method of continuous variation. This displayed a clear inflection point (see Figure S24), indicative of complex formation. While we have been unable to obtain additional structural information by X‐ray crystallography, we assume that Al^3+^ ions bind in between the two adjacent oxygen sites on **3**, similarly to the structures reported by Zhang et al.^[^ ^37^ ^]^ Given the use of donor solvents, these may also be supported by donating MeCN or H_2_O ligands. By contrast, a Job plot for **3** and FeCl_3_ showed no inflection point (see Figure S23). These data are consistent with a dynamic quenching mechanism for Fe^3+^, rather than binding of the metal ion. This alternative mechanism yields a “turn‐off” response for Fe^3+^, rather than the “turn‐on” response observed for Al^3+^. Indeed, the majority of previously reported fluorescent sensors for Al^3+^ rely on a “turn‐on” response upon binding of Al^3+^ between hard donor sites,^[^ ^9^ ^]^ while a “turn‐off” response to Fe^3+^ is more common due to the paramagnetic nature of Fe^3+^.^[^ ^49^, ^50^ ^]^ However, dual‐responsive systems, in which different metal ions can be sensed by the same system via different mechanisms to produce different responses, are much scarcer. Such systems tend to be based on photo‐responsive stimuli or chemical reactions induced by the metal ion.^[^ ^51^, ^52^, ^53^ ^]^

**Figure 7:**
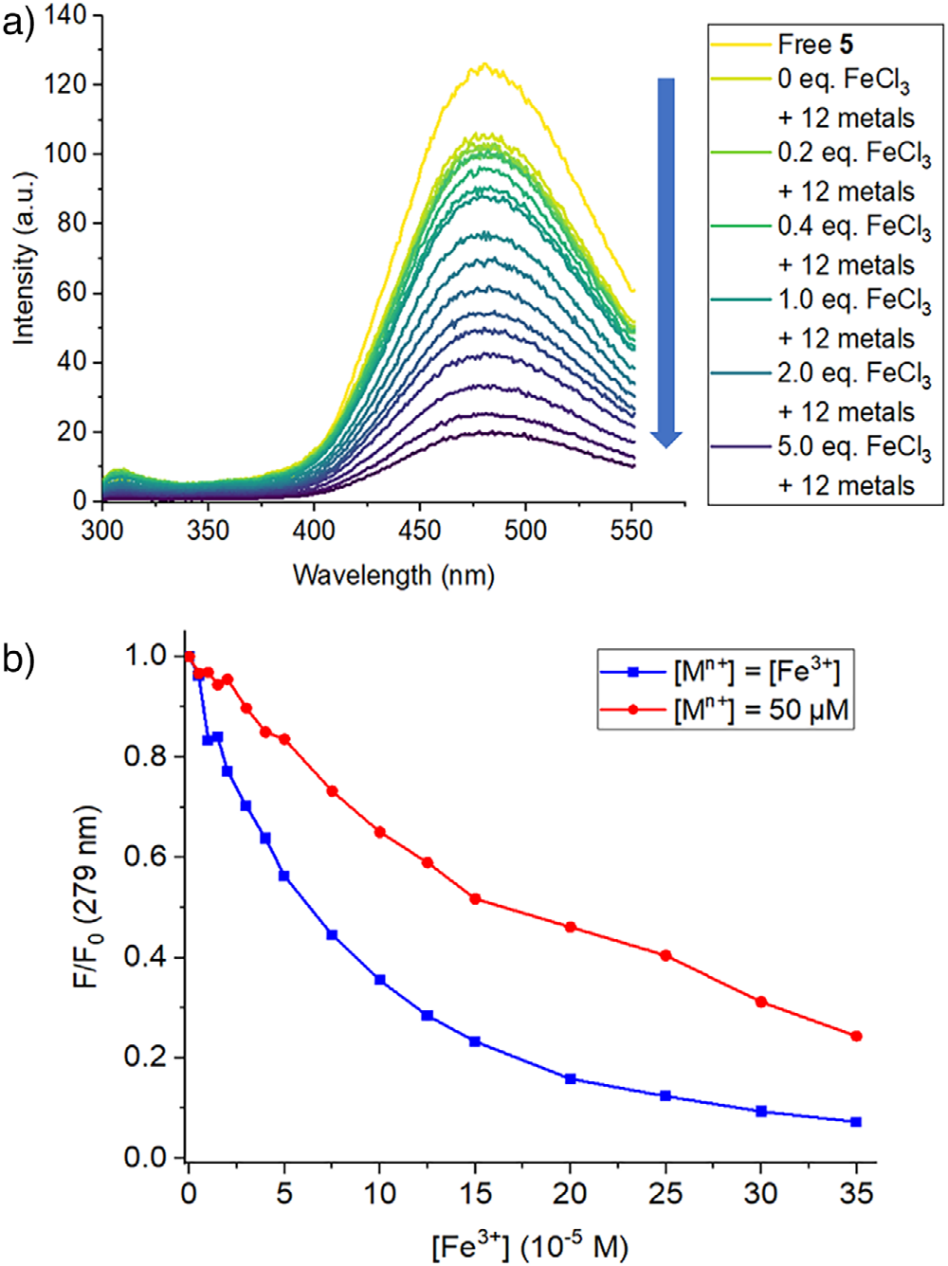
a) Fluorescence spectra for the titration of **5** (50 µM in 1:1 MeCN/H_2_O; *λ*_exc_ = 279 nm) with FeCl_3_ (0–7 equiv.) in the presence of 12 other metal ions (all 1 equiv.). b) Fluorescence intensities (*F*/*F*_0_ values) for the titration of **5** (50 µM in 1:1 MeCN/H_2_O; *λ*_exc_ = 279 nm) with FeCl_3_ (0–7 equiv.). Blue squares: concentration of 12 other metal ions is increased concurrently with Fe^3+^; Red circles: concentration of 12 other metal ions is kept at 1 equivalent.

With **5** only showing a pronounced change in fluorescence in the presence of Fe^3+^, we investigated whether **5** was able to selectively detect Fe^3+^ in a solution of competing metal ions. A mixture of 12 different metal ions (Li^+^, Na^+^, K^+^, Mg^2+^, Ca^2+^, Cr^3+^, Mn^2+^, Fe^2+^, Cu^2+^, Zn^2+^, La^3+^, Al^3+^, each 1 equiv.) was thus added to **5**, followed by titration of this solution with Fe^3+^ (Figure 7). Addition of the competing ions to **5** yielded a minor drop in fluorescence intensity (*F*/*F*_0_ = 0.85). Stepwise addition of Fe^3+^ subsequently yielded significant decreases in intensity at higher concentrations of Fe^3+^ (*F*/*F*_0_ at 1 equiv. of Fe^3+^ = 0.71; *F*/*F*_0_ at 5 equiv. of Fe^3+^ = 0.27). As a control, the same experiment was then repeated, but with the concentrations of the other 12 metal ions increased alongside the concentration of Fe^3+^ (Figure 7b). This yielded comparable decreases in intensity when compared to when the concentration of the competing ions is held at 1 equivalent (Figure 7b; *F*/*F*_0_ at 1 equiv. of [M^n+^] = 0.56; *F*/*F*_0_ at 5 equiv. of [M^n+^] = 0.12). This highlights that quenching by Fe^3+^ is the dominant factor in the fluorescence quenching of **5** and demonstrates the selectivity of fluorescence quenching of **5** with Fe^3+^, even in the presence of competing ions.

**Figure 8:**
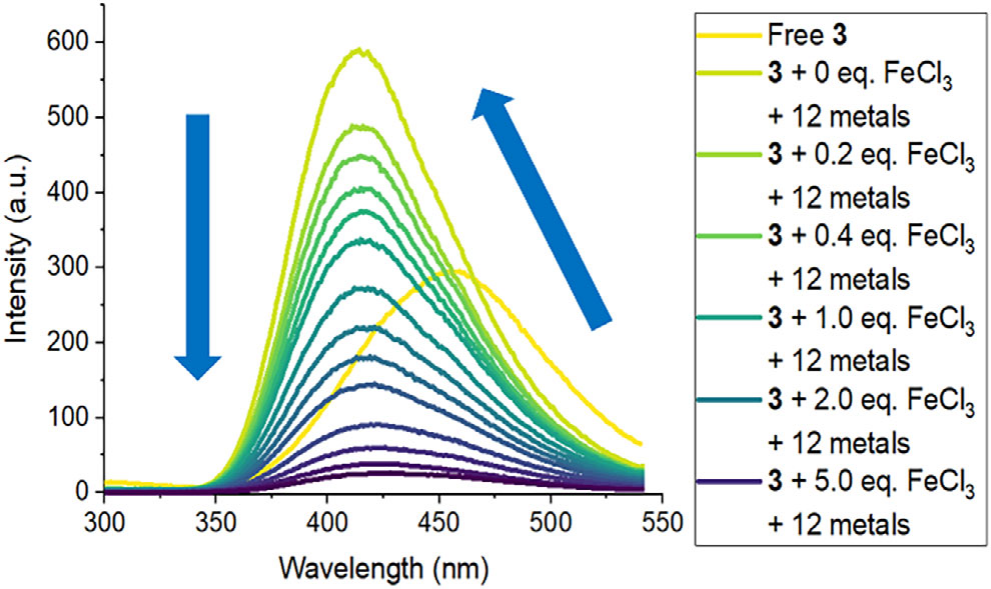
Fluorescence spectra for the titration of **3** (50 µM in 1:1 MeCN/H_2_O; *λ*_exc_ = 274 nm) with FeCl_3_ (0–7 equiv.) in the presence of 12 other metal ions (all 1 equiv.).

Given that **3** shows a response in the presence of Al^3+^ as well as Fe^3+^, we also sought to determine if **3** could also selectively detect Fe^3+^ in the presence of the same competing metal ions. A mixture of 12 competing metal ions (each 1 equiv.) was thus added to **3**, followed by titration of this solution with Fe^3+^ (Figure 8). Addition of the competing ions yielded a clear shift in emission wavelength from 461 to 411 nm and an increase in fluorescence intensity (*F*/*F*_0_ = 1.98). This indicates the formation of the **3**/Al^3+^ complex that displays enhanced fluorescence intensity, with partial quenching of this complex by the other metal ions present. Stepwise addition of Fe^3+^ subsequently gave significant decreases in fluorescence intensity (*F*/*F*_0_ at 1 equiv. of Fe^3+^ = 0.57; *F*/*F*_0_ at 5 equiv. of Fe^3+^ = 0.097). This demonstrates that **3** is also able to act as a selective fluorescent sensor for Fe^3+^. However, in the presence of Al^3+^, this occurs via the initial formation of a **3**/Al^3+^ complex, with the fluorescence of this new species subsequently subject to dynamic quenching by Fe^3+^.

The limits of detection of **3** and **5** for Fe^3+^ were subsequently calculated, both in the presence and absence of competing metal ions (see Section S2.9). The significant quenching effect of Fe^3+^ on **5** allows for detection limits in the submicromolar range. A detection limit of 0.39 µM was calculated in the absence of competing ions and of 0.48 µM in the presence of competing ions. The increased fluorescence intensity of **3** relative to **5** allows for even lower limits of detection; a detection limit of 0.13 µM was calculated in the absence of competing ions and of 0.16 µM in the presence of competing ions. These values are in line with the detection limits of other pyrazole‐based probes for Fe^3+^.^[^ ^25^ ^]^ Furthermore, to our knowledge, the Stokes shifts of **3** and **5** in MeCN/H_2_O (1:1) are the largest reported Stokes shifts to date for a fluorescent phosphorus‐based metal sensor.

## Conclusion

In conclusion, we have reported the synthesis of two new 3‐hydroxy‐1*H*‐pyrazol‐4‐yl)phosphine oxides via the hydrolysis of appropriately substituted phosphorus‐based heterocycles. A one‐pot methodology was developed to allow higher yields of the target products directly from air‐stable azophosphine–boranes. The proposed mechanism was supported by computational and experimental studies. These pyrazolyl phosphine oxides are an interesting new class of dual‐responsive fluorescent sensor for metal ions. Both sensors demonstrate significant “turn‐off” responses to Fe^3+^ with good selectivity, large Stokes shifts, and submicromolar limits of detection. The fluorescence quenching with Fe^3+^ is via a dynamic quenching mechanism and does not require complex formation. In contrast, a “turn‐on” response in the presence of Al^3+^ was observed, with pyrazolyl phosphine oxide **3** exhibiting a particularly significant enhancement in fluorescence, and this was shown to occur via a complexation process. We anticipate that this framework will open new avenues in the exploration of novel phosphorus‐based fluorophores and their applications in metal sensing.

## Supporting Information

The data associated with this manuscript are available at https://doi.org/10.25500/edata.bham.00001222. The authors have cited additional references within the Supporting Information.^[^ ^54^, ^55^, ^56^, ^57^, ^58^, ^59^, ^60^, ^61^, ^62^ ^]^

## Conflict of Interests

The authors declare no conflict of interest.

## Supporting information



Supporting Information

Supporting Information

Supporting Information

Supporting Information

Supporting Information

Supporting Information

## Data Availability

The data that support the findings of this study are openly available in [University of Birmingham eData Repository] at [https://edata.bham.ac.uk/1222], reference number [1222].
