# Supplementary material for: Dual‐Responsive Phosphorus‐Based Fluorescent Sensors: Synthesis and Selective Metal Sensing of Pyrazolyl Phosphine Oxides
Source: Angew Chem Int Ed Engl. 2025 Apr 7;64(23):e202501421. doi: 10.1002/anie.202501421 (PMC12124430; doi:10.1002/anie.202501421)
Supplement: Supplementary file 1 — Supporting Information [file ANIE-64-e202501421-s003.pdf]

Supporting Information for

Dual-responsive phosphorus-based fluorescent sensors: synthesis and selective metal sensing of pyrazolyl phosphine oxides

Ethan D. E. Calder, Isobel R. J. Hawes, Andrew R. Jupp

Contents

S1. Synthetic Details 2
S1.1. General Synthetic Information 2
S1.2. Synthesis of azophosphine-borane complexes (Method A) 4
S1.3. Synthesis of azophosphines (Method B) 6
S1.4. Synthesis of five-membered N/P-based heterocycles (Method C) 8
S1.5. Synthesis of pyrazolyl-phosphine oxides (Method D) 9
S1.6. Synthesis of **7** (MesN(NH_2)C(CO_2Et)=C(CH_3)(P(=O)(^{i}Pr_2))) 12
S1.7. Comparison of "step-wise" vs. "one-pot" synthesis of **3** 13
S1.8. Attempted reaction of **2** with H_2O 14
S1.9. Attempted reactions of **1** with other E-H (E = Si, O, N) containing substrates 15
S2. Photophysical properties of pyrazolyl-phosphine oxides 16
S2.1. General Information 16
S2.2. UV/Vis and Fluorescence Spectra for pyrazolyl-phosphine oxides 17
S2.3. Metal-ion sensing with pyrazolyl-phosphine oxides 19
S2.4. Titrations of **3** and **5** with $FeCl_3$ 24
S2.5. Titrations of **3** with $AlCl_3$ 28
S2.6. Job Plots for **3** with $FeCl_3$ and $AlCl_3$ 29
S2.7. Selectivity studies for **3** with $FeCl_3$ 31
S2.8. Selectivity studies for **5** with $FeCl_3$ 33
S2.9. Determination of detection limits for **3** and **5** with $FeCl_3$ 35
S3. Characterisation Data 37
S3.1. NMR Spectra 37
S3.2. UV/Vis Spectra for **4** and **7** 53
S3.3. Mass Spectra 54
S3.4. Attempts to characterise a complex between **3** and Al^{3+} with mass spectrometry ... 58
S3.5. IR Spectra 59
S4. Crystallographic Details 61
S4.1. General Crystallographic Information 61

S4.2. Tables of Crystallographic Data 62
S5. Computational Details 65
S5.1. General Computational Information 65
S5.2. Pertinent Energies and NBO Data on Structures 66
S5.3. TD-DFT Calculations on pyrazolyl-phosphine oxides 69
S5.4. Cartesian coordinates for optimised structures 70
S6. References 108

S1. Synthetic Details

S1.1. General Synthetic Information

Unless otherwise noted, all synthetic manipulations were carried out in an MBraun ECO glovebox under an inert atmosphere of dry, oxygen-free N_2, with water and oxygen levels maintained at < 0.1 ppm, or performed on the Schlenk line (using dry, oxygen-free N_2 as the inert gas) using standard Schlenk line procedures. All glassware and Teflon-coated stirrer bars were dried in a 180 °C oven overnight prior to use, unless otherwise stated. All molecular sieves are 3 Å and purchased from VWR chemicals, and were activated by heating at 400 °C under vacuum prior to use. Unless otherwise stated, degassing refers to three freeze-pump-thaw cycles.

Room temperature (RT) refers to reactions where no thermostatic control was applied and the temperature was recorded as 20–25 °C. Unless otherwise stated, overnight reactions refer to a period of 16 hours. Thin layer chromatography (TLC) analysis was performed using Machery-Nagel aluminium-backed silica plates. Spots were visualised by the quenching of ultraviolet light. All flash column chromatography was performed using Fluorochem 60 silica gel (particle size 40–63 μm) with a column of appropriate size.

Commercial reagents were purchased, suitably stored as specified by the supplier, and used as received, unless otherwise stated, from Sigma-Aldrich (*sec*-butyllithium, 1.42 M in cyclohexane; borane-di(tert-butyl)phosphine complex, 94%; pyrrolidine, anhydrous, > 99.5%, stored in an air-tight ampoule; borane dimethylsulfide complex; diisobutylaluminium hydride solution, 1.0 M in toluene; diethyl acetylenedicarboxylate, 97%), Fisher Scientific (sodium hydroxide, white pellets; magnesium sulfate, dried; tetrafluoroboric acid, ca. 50% w/w aqueous solution), Acros Organics (chlorodiisopropylphosphine, 96%), or Alfa Aesar (ethyl 2-butynoate, 98%; sodium nitrite, 98%; 2,4,6-trimethylaniline, 98%; *p*-anisidine, 99%). Solvents were purchased and used as received, unless otherwise stated, from Sigma-Aldrich (hexane, puriss, p.a., ACS reagent, reag. Ph. Eur.,

99+ (GC), degassed and stored in air-tight ampoules over 3 Å molecular sieves; diethyl ether, ACS reagent, 99.8%; acetonitrile, gradient grade, > 99.9%; chloroform-d_3, 99.8 atom % D, dried by overnight reaction with CaH_2, degassed and stored in an air-tight ampoule over 3 Å molecular sieves), Fisher Scientific (dichloromethane (DCM), 99.8% HPLC grade), or VWR Chemicals (ethyl acetate, ≥99%, GPR RECTAPUR). Toluene was obtained from the laboratory solvent purification system, degassed, and stored in air-tight ampoules over 3 Å molecular sieves. THF was obtained from the laboratory solvent purification system, degassed, dried over Na/benzophenone, and stored in air-tight ampoules over 3 Å molecular sieves. Deionised water for synthesis (not for analytical purposes) was obtained from an Elga DV35 Purelab. Mesitylene diazonium tetrafluoroborate ($[MesN_2][BF_4]$), *para*-methoxyphenyl diazonium tetrafluoroborate ($[p$-$OMe(C_6H_4)N_2][BF_4]$), and borane di(iso-propyl)phosphine complex ($H^iPr_2P.BH_3$) were synthesised in accordance with previously reported literature.[1]

Unless otherwise stated, all NMR spectra used for characterisation were collected at 298 K on a Bruker 500 MHz AV_NEO Advance NMR Spectrometer or a Bruker 400 MHz AV_NEO Advance NMR Spectrometer. Chemical shifts are reported in parts per million (ppm), coupling constants (J) are reported in Hz. ^1H and $^{13}C\{^1H\}$ NMR spectra were referenced internally to the most upfield solvent peak; ^{31}P and $^{31}P\{^1H\}$ NMR spectra are externally referenced to 85% H_3PO_4; $^{11}B\{^1H\}$ NMR spectra are externally referenced to $BF_3.OEt_2$. All shifts and coupling constants are given to one or two decimal places.

UV/Vis absorption spectra for products **4** and **7** were collected on an Agilent Cary60 UV/Vis Spectrometer. The samples were prepared as 5 x 10^{-5} M solutions in toluene and irradiated in a quartz glass High Precision Cell cuvette (10 x 10 mm) from Hellma Analytics. Air-sensitive UV-Vis samples were collected using a sealable cuvette, with solutions made up in the glovebox. The wavelength of maximum absorption (λ_{max}) is reported in nm, along with the extinction coefficient in mol.dm^{-3}.cm^{-1}. All other UV/Vis absorption spectra are discussed separately in section S2.

All IR spectra were collected on a Perkin Elmer Spectrum Two FT-IR spectrometer using attenuated total reflection (ATR) sampling technique. Absorption maxima are reported in wavenumbers (cm^{-1}).

All mass spectra were collected on a Waters Xevo G2-XS TOF mass spectrometer using electrospray ionisation (ESI) positive ion mode. Samples were dissolved either in dry dichloromethane or acetonitrile, and directly injected into the ESI ionisation chamber via a 250 µL glass syringe. High resolution mass spectra were obtained using the same method, and are reported to four decimal places.

All elemental analysis samples were collected on a CE Instruments EA1110 elemental analyser.

Unless otherwise stated, single-crystal X-ray diffraction data were collected at 100 K on a Rigaku SuperNova diffractometer using an Atlas detector, using Cu-Kα radiation (λ = 1.54184). The data collections for these species were driven and processed and absorption corrections were applied using face indexing with a Gaussian absorption correction in CrysAlisPro.[2] Using OLEX2,[3] the structures were solved using ShelXT,[4] and were refined by a full-matrix least-squares procedure on F^2 in ShelX programmes.[5] All non-hydrogen atoms were refined with anisotropic displacement parameters. Further details relating to crystal structures can be found in section S4.

S1.2. Synthesis of azophosphine-borane complexes (Method A)

This procedure is based on the previously reported literature.[6] A 25 mL Schlenk flask was charged with $HR_2P.BH_3$ (R = ^{i}Pr, ^{t}Bu; 1 equiv.), and freshly distilled THF was added to create a 0.7 M solution of $HR_2P.BH_3$ in THF. This solution is cooled to –78 °C, and a 1.4 M cyclohexane solution of *sec*-butyllithium (1 equiv.) was added dropwise, resulting in a colour change from colourless to pale yellow. The solution was allowed to warm to room temperature over one hour, and then stirred for a further three hours at room temperature; after this time, a colour change back to a colourless/off-white solution was observed. This solution was then added dropwise to a separate 50 mL Schlenk flask containing a slurry of $[ArN_2]^+[BF_4]^-$ (Ar = Mes, *p*-OMe(C_6H_4); 1 equiv.) in freshly distilled THF (0.25 M) at –78°C, resulting in an immediate colour change. The reaction was left to warm slowly to room temperature over the course of one hour. The volatiles were removed in vacuo, with the materials now being treated as air-stable. The product was then isolated by column chromatography. Removal of the volatiles *in vacuo* yielded the product as an intensely coloured solid or oil. The air-stable, solid products were stored in vials, away from direct light, in the freezer.

Synthesis of $MesN_2P(^tBu)_2.BH_3$

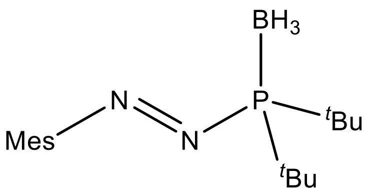

$MesN_2P(^tBu)_2.BH_3$ was synthesised from commercially available precursor $H^tBu_2P.BH_3$ (0.43 g, 2.68 mmol) according to method A, with purification by column chromatography (eluent = 95:5 hexane:Et_2O, with the product appearing as a purple spot with R_f = 0.35 in this eluent system), and the product yielded as a purple solid (488 mg, 1.61 mmol, 60% yield). ^{1}H (400.1 MHz, $CDCl_3$, 298 K): δ 6.96 (s, 2H; H_{Ar} (Mes)), δ 2.47 (s, 6H; *o*-CH_3 (Mes)), δ 2.34 (s, 3H; *p*-CH_3 (Mes)), δ 1.41 (d, $^3J_{H\text{–}P}$ = 12 Hz, 18H; ^{t}Bu), δ 0.56 (br. quart., 3H; BH_3). $^{31}P\{^1H\}$ (162.0 MHz, $CDCl_3$): δ 108.5 (br. quart.). $^{11}B\{^1H\}$ (128.4 MHz, $CDCl_3$): δ - 42.5 (d, $^1J_{B\text{–}P}$ = 51 Hz). Obtained data in accord with reported literature.[6]

Synthesis of $MesN_2P(^iPr)_2.BH_3$

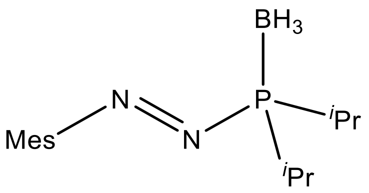

$MesN_2P(^iPr)_2.BH_3$ was synthesised from precursor $H^iPr_2P.BH_3$ (0.402 g, 3.04 mmol) according to method A, with purification by column chromatography (eluent = 97:3 hexane:Et_2O), with the product appearing as a purple spot with R_f = 0.38 in this eluent system; the residual starting material ($H^iPr_2P.BH_3$) had an Rf = 0.30, and could be identified by TLC by staining in $KMnO_4$), with the product yielded as a purple oil (0.321 g, 1.16 mmol, 38 % yield). ^{1}H (400.1 MHz, $CDCl_3$, 298 K): δ 6.95 (2H, br. doub., $^6J_{H\text{-}P}$ = 0.6 Hz; *m*-ArH), δ 2.52 (2H, m; $P(CH(CH_3)_2)_2$), δ 2.40 (6H, s; *o*-CH_3), δ 2.33 (3H, s; *p*-CH_3), δ 1.32 (12H, m; $CH(CH_3)_2$), δ 0.53 (3H, br. quart., $^1J_{B\text{-}H}$ = 95.4 Hz; BH_3). $^{31}P\{^1H\}$ (162.0 MHz, $CDCl_3$, 298 K): δ 102.4 (br. quart). $^{11}B\{^1H\}$ (128.4 MHz, $CDCl_3$, 298 K): δ -43.6 (d, $^1J_{B\text{-}P}$ = 51.3 Hz). Obtained data in accord with reported literature.[1]

Synthesis of *p*-OMe(C_6H_4)$N_2P(^iPr)_2.BH_3$ (4)

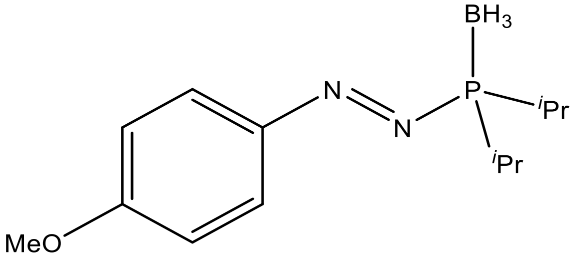

p-OMe(C_6H_4)$N_2P(^iPr)_2.BH_3$ was synthesised from precursor $H^iPr_2P.BH_3$ (0.385 g, 2.92 mmol) according to method A, with purification by column chromatography (eluent = 1:2 hexane:dichloromethane, with the product appearing as a purple spot with R_f = 0.25 in this eluent system), and the product yielded as an orange solid (0.206 g, 0.774 mmol, 27 % yield). Single crystals of **4** were grown by slow evaporation of a $CDCl_3$ solution of **4**. $H^iPr_2P.BH_3$ was also synthesised in accordance with reported literature.[1] ^{1}H (400.1 MHz, $CDCl_3$, 298 K): δ 7.82 (2H, d, $^3J_{H\text{-}H}$ = 9.0 Hz; *o*-Ar*H*), δ 6.98 (2H, d, $^3J_{H\text{-}H}$ = 9.1 Hz; *m*-Ar*H*), δ 3.90 (3H, s; O-C*H*$_3$), δ 2.52 (2H, m; P(C*H*($CH_3)_2)_2$), δ 1.31 (6H, dd, $^2J_{H\text{-}P}$ = 14.2 Hz, $^3J_{H\text{-}H}$ = 7.3 Hz; CH(C*H*$_3)_2$), δ 1.27 (6H, dd, $^2J_{H\text{-}P}$ = 13.4 Hz, $^3J_{H\text{-}H}$ = 7.1 Hz; CH(C*H*$_3)_2$), δ 0.53 (3H, br. quart., $^1J_{B\text{-}H}$ = 103.6 Hz; B*H*$_3$). ^{13}C{^{1}H} (125.7 MHz, $CDCl_3$, 298 K): δ 163.8 (s; *p*-C_{Ar} (Mes)), δ 149.8 (d, $^3J_{C\text{-}P}$ = 37.1 Hz; *i*-C_{Ar}(Mes)), δ 125.0 (s; *o*-C_{Ar} (Mes)), δ 114.3 (s; *m*-C_{Ar} (Mes)), δ 55.9 (s; O-CH_3), δ 23.6 (d, $^1J_{C\text{-}P}$ = 31.0 Hz; *C*H($CH_3)_2$), δ 16.7-16.8 (m; CH(*C*H$_3)_2$). ^{31}P{^{1}H} (202.4 MHz, $CDCl_3$, 298 K): δ 99.5 (br. quart). ^{31}P (162.0 MHz, $CDCl_3$, 298 K): δ 99.5 (br. s). ^{11}B{^{1}H} (160.4 MHz, $CDCl_3$, 298 K): δ -44.2 (d, $^1J_{B\text{-}P}$ = 52.9 Hz). UV-Vis Spectroscopy: (5 x 10^{-5}M in toluene): 335.01 nm, 0.9014 absorption; 476.98 nm, 0.0102 absorption. ESI-HRMS m/z for $C_{13}H_{23}N_2OPB$ $[M-\mathrm{H}]^+$: calcd. 264.1677, 265.1643, 266.1673; found 264.1677, 265.1643, 266.1726. Additional peaks: $[MesN_2P^iPr_2]^+$: found 253.15; $[MesN_2P^iPr_2.BH_3 + H]^+$: found 266.1834, 267.1800, 268.1830; found 266.1726, 267.1797, 268.1828. IR: ν_{max} (cm–1) 2970.8 (m), 2367.5 (m; B-H), 2336.9 (m; B-H), 2313.8 (m; B-H), 1600.7 (m; N=N)

S1.3. Synthesis of azophosphines (Method B)

This procedure is based on the previously reported literature.[6] The relevant azophosphine-borane $MesN_2PR_2.BH_3$ (R = ^{t}Bu, ^{i}Pr; 1 equiv.) was added to a 50 mL Schlenk flask and dissolved in toluene, creating a 0.05 M solution of azophosphine-borane in toluene. To this, pyrrolidine was added (10 equiv.), and the mixture stirred at room temperature overnight (16 hours), which gave 100%

conversion to $MesN_2PR_2$ by crude $^{31}P\{^1H\}$ NMR spectroscopy. Excess pyrrolidine was removed *in vacuo* and the resulting oil filtered through a short silica plug in the glovebox (eluent = 90:10 hexane:Et_2O) to remove the pyrrolidine.BH_3 adduct. Removal of the eluent in vacuo obtained the target deprotected $MesN_2PR_2$ product as an intensely coloured solid or oil. The air-sensitive products were stored in vials in the glovebox freezer.

Synthesis of MesN$_2$P(^{i}Pr)$_2$

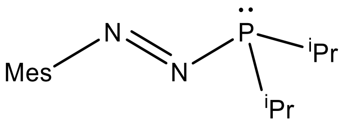

$MesN_2P(^iPr)_2$ was synthesised from precursor $MesN_2P(^iPr)_2.BH_3$ (167 mg, 0.60 mmol) according to method B. After overnight stirring, a colour change from purple to red was observed. The product was isolated as a red oil (116 mg, 0.44 mmol, 74 % yield). Removal of the residual pyrrolidine-borane adduct was determined by $^{11}B\{^1H\}$ NMR spectroscopy, which showed no peaks. ^1H (400.1 MHz, tol-d_8, 298 K): δ 6.69 (2H, s; *m*-Ar*H*), δ 2.33 (6H, s; *o*-C*H*$_3$), δ 2.19 (2H, septet of doublets, $^3J_{H\text{-}H}$ = 7.1 Hz, $^2J_{H\text{-}P}$ = 1.2 Hz; P(C*H*(CH$_3$)$_2$)$_2$), δ 2.10 (3H, s; *p*-C*H*$_3$), δ 1.22 (12H, m; CH(C*H*$_3$)$_2$). $^{31}P\{^1H\}$ (162.0 MHz, tol-d_8, 298 K): δ 106.9 (m). Obtained data in accord with reported literature.[1]

Synthesis of MesN$_2$P(^{t}Bu)$_2$

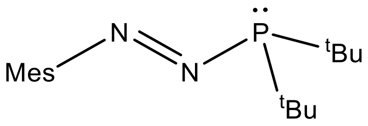

$MesN_2P(^tBu)_2$ was synthesised from precursor $MesN_2P(^tBu)_2.BH_3$ (169 mg, 0.55 mmol) according to method B. After overnight stirring, a colour change from purple to red was observed. The product was isolated as a red oil (127 mg, 0.43 mmol, 79 % yield). Removal of the residual pyrrolidine-borane adduct was determined by $^{11}B\{^1H\}$ NMR spectroscopy, which showed no peaks. ^1H (400.1 MHz, tol-d_8, 298 K): δ 6.71 (m, 2H; *m*-H_{Ar} (Mes)), δ 2.38 (s, 6H; *o*-CH_3 (Mes)), δ 2.10 (s, 3H; *p*-CH_3(Mes)), δ 1.32 (d, $^3J_{H\text{–}P}$ = 11 Hz, 18H; ^{t}Bu). $^{31}P\{^1H\}$ (162.0 MHz, tol-d_8): δ 118.3 (s). Obtained data in accord with reported literature.[6]

S1.4. Synthesis of five-membered N/P-based heterocycles (Method C)

In the glovebox, the relevant azophosphine ($MesN_2P^tBu_2$, or $MesN_2P^iPr_2$; 1 equiv.) was added to a 25 mL Schlenk flask and dissolved in toluene, creating a 0.05 M solution of azophosphine in toluene. Diethyl acetylenedicarboxylate ($C_2(CO_2Et)_2$; 1 equiv)) was then added to this solution, giving an immediate colour change to pale yellow and 100% conversion of the azophosphine by crude $^{31}P\{^1H\}$ NMR spectroscopy. Removal of the solvent *in vacuo* yielded the heterocyclic products as beige/pale yellow coloured solids.

Synthesis of $MesN_2P(^iPr)_2C_2(CO_2Et)_2$ (1)

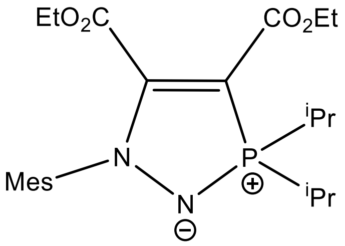

Five-membered heterocycle **1** was synthesised from precursor $MesN_2P(^iPr)_2$ (82 mg, 0.30 mmol) according to method C. The product was isolated as an air-sensitive beige/pale-yellow coloured solid (91 mg, 0.22 mmol, 72 % yield). ^1H (400.1 MHz, tol-d_8, 298 K): δ 6.62 (2H, s; m-Ar*H*), δ 4.09 (2H, q, $^3J_{H\text{-}H}$ = 7.01 Hz; $CO_2CH_2CH_3$), δ 3.85 (2H, q, $^3J_{H\text{-}H}$ = 7.08 Hz; $CO_2CH_2CH_3$), δ 2.40 (6H, s; o-CH_3), δ 2.22 (2H, m; $CH(CH_3)_2$), δ 2.02 (3H, s; p-CH_3), δ 1.19 (6H; dd, $^3J_{H\text{-}P}$ = 17.6 Hz, $^3J_{H\text{-}H}$ = 7.23 Hz; $CH(CH_3)_2$), δ 1.12 (6H; dd, $^3J_{H\text{-}P}$ = 16.1 Hz, $^3J_{H\text{-}H}$ = 7.18 Hz; $CH(CH_3)_2$), δ 1.03 (3H, t, $^3J_{H\text{-}H}$ = 7.17 Hz; $CO_2CH_2CH_3$), δ 0.75 (3H, t, $^3J_{H\text{-}H}$ = 7.23 Hz; $CO_2CH_2CH_3$). $^{31}P\{^1H\}$ (162.0 MHz, tol-d_8, 298 K): δ 74.9 (s). Obtained data in accord with reported literature.[1]

Synthesis of $MesN_2P(^tBu_2)_2C_2(CO_2Et)_2$ (2)

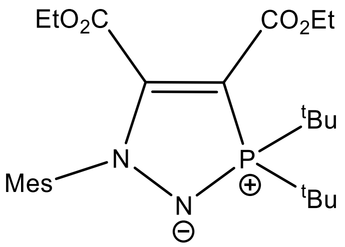

Five-membered heterocycle **2** was synthesised from precursor $MesN_2P(^tBu)_2$ (117 mg, 0.40 mmol) according to method C. The product was isolated as an air-stable beige/pale-yellow

coloured solid (153 mg, 0.34 mmol, 86 % yield). ^{1}H (400.1 MHz, d_8-toluene, 298 K): δ 6.62 (2H, br. doub., $^{6}J_{H\text{-}P}$ = 0.6 Hz; *m*-Ar*H*), δ 4.10 (2H, q, $^{3}J_{H\text{-}H}$ = 7.1 Hz; $CO_2CH_2CH_3$), δ 3.83 (2H, q, $^{3}J_{H\text{-}H}$ = 7.1 Hz; $CO_2CH_2CH_3$), δ 2.42 (6H, s; *o*-CH_3), δ 2.02 (3H, s; *p*-CH_3), δ 1.34 (18H, d, $^{3}J_{H\text{-}P}$ = 15.0 Hz; $P(C(CH_3)_3)_2$), δ 1.04 (3H, t, $^{3}J_{H\text{-}H}$ = 7.1 Hz; $CO_2CH_2CH_3$), δ 0.73 (3H, t, $^{3}J_{H\text{-}H}$ = 7.1 Hz; $CO_2CH_2CH_3$). $^{31}P\{^{1}H\}$ (162.0 MHz, d_8-toluene, 298 K): δ 77.5 (s). Obtained data in accord with reported literature.[1]

S1.5. Synthesis of pyrazolyl-phosphine oxides (Method D)

A 50 mL Schlenk flask was charged with the relevant azophosphine-borane ($ArN_2P(^{i}Pr)_2.BH_3$; Ar = Mes, *p*-OMe(C_6H_4)), and dissolved in toluene, creating a 0.05 M solution of azophosphine-borane in toluene. 10 equiv. of pyrrolidine were then added to the reaction mixture. This solution was left stirring at room temperature overnight (16 hours). The solvent was then removed in vacuo, with further drying in vacuo for an hour to ensure removal of the excess pyrrolidine from the reaction vessel. The remaining material was re-dissolved in THF, creating a 0.05 M solution of azophosphine-borane in THF, and diethyl acetylenedicarboxylate ($(C(CO_2Et))_2$; 1 equiv.) was added to the reaction vessel. Degassed water (100 equiv.) was then added directly into the reaction mixture, with subsequent stirring at room temperature for 4 hours. The solvent was removed *in vacuo*, and the crude product purified by running through a short silica plug. The product fractions were collected and the solvent then removed *in vacuo*, with further drying under vacuum overnight, to yield the product as an air- and water-stable solids.

Synthesis of $MesN_2C(OH)C(P(^{i}Pr_2))C(CO_2Et)$ (3)

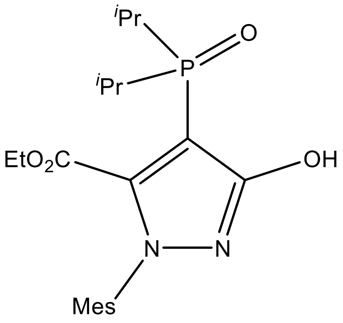

*Procedure for synthesis of **3**, Method D:*

Pyrazolyl-phosphine oxide **3** was synthesised from precursor $MesN_2P(^{i}Pr)_2.BH_3$ (350 mg, 1.26 mmol) according to method D. After overnight stirring with pyrrolidine, a colour change from purple to red was observed. After reaction with $(C(CO_2Et))_2$, a colour change from red to pale

yellow was observed immediately. After addition of degassed water and stirring for 4 hours, a colour change from yellow to a colourless solution was observed, the crude product. The crude product was purified by elution through a short silica plug, eluting with 50:50 hexane:EtOAc (R_f of **3** = 0.42, visible as a fluorescent blue spot under a short-wave UV lamp). The product fractions were collected and the solvent then removed *in vacuo*, with further drying under vacuum overnight, to yield the product as an air-stable, colourless solid (331 mg, 0.82 mmol, 65% yield). Single crystals of **3** were grown either by slow evaporation of a concentrated *n*-hexane solution of **3** (yielding **3α**), or by of slow evaporation of a solution of **3** in THF:hexane (approximately 1:5 v/v) (yielding **3β**). See section S4.1 for further details.

*Procedure for synthesis of **3**, from isolated heterocycle **1**:*

Alternatively, pyrazolyl-phosphine oxide **3** could also be synthesised directly from five-membered heterocycle **1**. In the glovebox, five-membered heterocycle **1** (78 mg, 0.18 mmol) was dissolved in 3 mL of THF in a Schlenk flask. This flask was then cycled onto the Schlenk line, and charged with 0.33 mL of degassed water (18 mmol; 100 equiv.). This solution was left stirring at room temperature for 4 hours, yielding the crude product. The crude product was purified by elution through a short silica plug, eluting with 50:50 hexane:EtOAc (R_f of **3** = 0.42, visible as a fluorescent blue spot under a short-wave UV lamp). The product fractions were collected and the solvent then removed *in vacuo*, with further drying under vacuum overnight, to yield the product as an air-stable, colourless solid (53 mg, 0.13 mmol, 72% yield).

^{1}H (500.1 MHz, $CDCl_3$, 298 K): δ 11.1 (1H, s; O-*H*), δ 6.90 (2H, s; m-Ar*H*), δ 4.04 (2H, q, $^3J_{H\text{-H}}$ = 7.06 Hz; $CO_2CH_2CH_3$), δ 2.51 (2H, m; C*H*(CH_3)$_2$), δ 2.31 (3H, s; p-C*H*$_3$), δ 1.95 (6H, s; o-C*H*$_3$), δ 1.30 (6H, dd, $^3J_{H\text{-P}}$ = 16.2 Hz, $^3J_{H\text{-H}}$ = 7.13 Hz; CH(C*H*$_3$)$_2$), δ 1.13 (6H, dd, $^3J_{H\text{-P}}$ = 17.2 Hz, $^3J_{H\text{-H}}$ = 7.14 Hz; CH(C*H*$_3$)$_2$), δ 0.91 (3H, t, $^3J_{H\text{-H}}$ = 7.13 Hz; $CO_2CH_2CH_3$). ^{13}C{^{1}H} (125.8 MHz, $CDCl_3$, 298 K): δ 168.0 (d, $^2J_{C\text{-P}}$ = 3.3 Hz; *C*-OH), δ 158.7 (s, *C*=O), δ 139.1 (s, *p*-C_{Ar}(Mes)), δ 136.9 (s, *i*-C_{Ar}(Mes)), δ 135.3 (s, *o*-C_{Ar} (Mes)), δ 134.3 (d, $^2J_{C\text{-P}}$ = 13.4 Hz; *C*-C=O), δ 128.6 (s, *m*-C_{Ar} (Mes)), δ 91.3 (d, $^1J_{C\text{-P}}$ = 99.9 Hz; P-C_{Ar}), δ 61.8 (s, $CO_2CH_2CH_3$), δ 27.4 (d, $^1J_{C\text{-P}}$ = 69.4 Hz; P*C*(CH_3)$_2$), δ 21.3 (s, *p*-*C*H_3(Mes)), δ 17.1 (s, *o*-CH_3(Mes)), δ 16.8 (d, $^2J_{C\text{-P}}$ = 2.9 Hz; PC(CH_3)$_2$, δ 15.3 (d, $^2J_{C\text{-P}}$ = 3.2 Hz; PC(*C*H_3)$_2$), δ 13.5 (s, $CO_2CH_2CH_3$). ^{31}P{^{1}H} (202.4 MHz, $CDCl_3$, 298 K): δ 62.3 (s). ^{31}P (202.4 MHz, $CDCl_3$, 298 K): δ 62.3 (m). ESI-HRMS m/z for $C_{21}H_{31}O_4N_2P$ $[M + \mathrm{H}]^+$: calcd. 407.2100, 408.2132, 409.2159; found 407.2102, 408.2129, 409.2157. IR: ν_{max} (cm^{-1}) 2964.8 (m), 2925.0 (m), 2871.1 (m), 1708.6 (m; C=O). Elemental Analysis Found (Calculated) for $C_{21}H_{31}O_4N_2P$: C, 62.00(62.06); H, 7.57(7.69); N, 6.65(6.89).

Synthesis of *p*-OMe(C_6H_4)N_2C(OH)C(P($^iPr_2$))C(CO_2Et) (5)

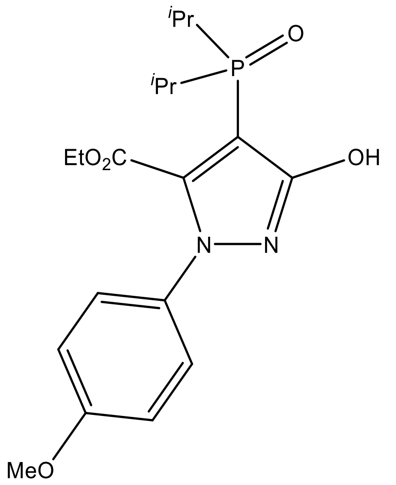

Pyrazolyl-phosphine oxide **5** was synthesised from precursor *p*-OMe(C_6H_4)N_2P(^iPr)$_2$.BH_3 (**4**; 152 mg, 0.57 mmol) according to method D. After overnight stirring with pyrrolidine, a colour change from orange to red was observed. After reaction with (C(CO_2Et))$_2$, a colour change from red to yellow was observed immediately. After addition of degassed water and stirring for 4 hours, a colour change from yellow to a colourless solution was observed. The solvent was removed *in vacuo*, and the crude product was then run through a short silica plug, eluting with 100% EtOAc (R_f of **5** = 0.50, visible as a fluorescent blue spot under a UV lamp). The product fractions were collected and the solvent then removed *in vacuo*, with further drying under vacuum overnight, to yield the product as an air-stable, off-white solid (126 mg, 0.32 mmol, 56% yield). Single crystals of **5** were grown by slow evaporation of a concentrated *n*-hexane solution of **5**. ^{1}H (400.1 MHz, $CDCl_3$, 298 K): 11.1 (1H, s; O*H*), 7.28 (2H, d, $^3J_{H-H}$ = 8.9 Hz; o-Ar-*H*), 6.92 (2H, d, $^3J_{H-H}$ = 8.9 Hz; m-Ar-*H*), 4.12 (2H, q, $^3J_{H-H}$ = 7.0 Hz; C(=O)OC*H*$_2$CH$_3$), 3.85 (3H, s; ArOC*H*$_3$), 2.48 (2H, m; PC*H*(CH$_3$)$_2$), 1.29 (6H, dd, $^3J_{H-P}$ = 16.1 Hz, $^3J_{H-H}$ = 7.1; PCH(C*H*$_3$)$_2$), 1.14 (6H, dd, $^3J_{H-P}$ = 17.3 Hz, $^3J_{H-H}$ = 7.2; PCH(C*H*$_3$)$_2$), 1.05 (3H, t, $^3J_{H-H}$ = 7.2 Hz; C(=O)OCH$_2$C*H*$_3$)). ^{13}C{^{1}H} (125.7 MHz, $CDCl_3$, 298 K): δ 167.6 (d, $^2J_{C-P}$ = 3.3 Hz; *C*-OH), δ 159.9 (s, *p-C*$_{Ar}$), δ 159.4 (s, *C*(=O)), δ 133.5 (s, *i*-C$_{Ar}$), δ 133.3 (d, $^2J_{C-P}$ = 13.6 Hz; C(=O)-*C*-C-P(=O)), δ 127.1 (s, *o*-C$_{Ar}$), δ 113.9 (s, *m*-C$_{Ar}$), δ 92.6 (d, $^1J_{C-P}$ = 99.4 Hz; P-C$_{Ar}$), δ 62.1 (s, O*C*H$_2$CH$_3$), δ 55.7 (s, O-*C*H$_3$), δ 27.7 (d, $^1J_{C-P}$ = 69.2 Hz; P*C*(CH$_3$)$_2$) δ 16.6 (d, $^2J_{C-P}$ = 3.3 Hz; PC(*C*H$_3$)$_2$), δ 15.2 (d, $^2J_{C-P}$ = 3.3 Hz; PC(*C*H$_3$)$_2$), δ 13.7 (s, CO$_2$CH$_2$*C*H$_3$). ^{31}P{^{1}H} (202.4 MHz, $CDCl_3$, 298 K): δ 62.5 (s). ^{31}P (202.4 MHz, $CDCl_3$, 298 K): δ 62.5 (m). ESI-HRMS m/z for $C_{19}H_{27}N_2O_5P$, [M + H]$^+$: calcd. 395.1736, 396.1768, 397.1793; found 395.1736, 396.1768, 397.1793. IR: ν_{max} (cm^{-1}) 2926.8 (m), 1720.8 (m; C=O). Elemental Analysis Found (Calculated) for $C_{19}H_{27}O_5N_2P$: C, 58.17(57.86); H, 7.12(6.90); N, 7.08(7.10).

S1.6. Synthesis of **7**
$(MesN(NH_2)C(CO_2Et)=C(CH_3)(P(=O)(^iPr_2)))$

In the glovebox, azophosphine $MesN_2P^iPr_2$ (60 mg, 0.23 mmol, 1 equiv) was dissolved in 2.5 mL of toluene in a Schlenk flask. Ethyl 2-butynoate (80 µL; 0.68 mmol; 3 equivs) is then added to this solution *via* micropipette, and subsequently left stirring for 3 days in the glovebox at room temperature. After this time, a colour change from red to orangish-yellow was observed. The flask was cycled onto the Schlenk line, and charged with degassed water (41 µL; 2.3 mmol; 10 equiv.). This was left stirring for two hours, after which time a colour change to pale yellow/off-white was observed, with the solvent removed *in vacuo* to yield an off-white oil. This crude product was then run through a short silica plug, eluting with 100% EtOAc (R_f of **7** = 0.37, visible as a UV-active spot). The product fractions are collected and the solvent is then removed *in vacuo*, with further drying under vacuum overnight, to yield the product as an air-stable colourless oil (42 mg, 0.11 mmol, 47% yield). Single crystals of **7** were grown by slow evaporation of a concentrated *n*-hexane solution of **7**. ^1H (400.1 MHz, $CDCl_3$, 298 K): δ 6.76 (2H, s; m-Ar*H*), δ 5.85 (2H, br. s; NH_2), δ 3.82 (2H, q, $^3J_{H\text{-}H}$ = 7.2 Hz; $CO_2CH_2CH_3$), δ 2.32 (6H, s; o-CH_3), δ 2.25 (2H, m; $CH(CH_3)_2$), δ 2.20 (3H, s; p-CH_3), δ 1.63 (3H, d, $^3J_{H\text{-}P}$ = 10.9 Hz; C=C-CH_3), δ 1.30 (6H, dd, $^3J_{H\text{-}P}$ = 14.8 Hz, $^3J_{H\text{-}H}$ = 7.0 Hz; $CH(CH_3)_2$), δ 1.26 (6H, dd, $^3J_{H\text{-}P}$ = 15.5 Hz, $^3J_{H\text{-}H}$ = 7.3 Hz; $CH(CH_3)_2$), δ 0.89 (3H, t, $^3J_{H\text{-}H}$ = 7.2 Hz; $CO_2CH_2CH_3$). $^{13}C\{^1H\}$ (100.6 MHz, $CDCl_3$, 298 K): δ 165.7 (d, $^3J_{C\text{-}P}$ = 14.7 Hz; *C*=O), δ 155.9 (s; N-*C*=C-P), δ 140.9 (s, *i*-C_{Ar}(Mes)), δ 137.4 (s, *o*-C_{Ar} (Mes)), δ 137.2 (s, *p*-C_{Ar}(Mes)), δ 129.1 (s, *m*-C_{Ar} (Mes)), δ 86.2 (d, $^1J_{C\text{-}P}$ = 88.2 Hz; N-C=*C*-P), δ 61.1 (s, $CO_2CH_2CH_3$), δ 26.6 (d, $^1J_{C\text{-}P}$ = 68.5 Hz; $PCH(CH_3)_2$), δ 21.1 (s, *p*-CH_3(Mes)), δ 18.5 (s, *o*-CH_3(Mes)), δ 17.4 (d, $^2J_{C\text{-}P}$ = 2.6 Hz; $PCH(CH_3)_2$), δ 16.6 (d, $^2J_{C\text{-}P}$ = 10.1 Hz; C=C-CH_3), δ 16.2 (d, $^2J_{C\text{-}P}$ = 2.8 Hz; $PCH(CH_3)_2$), δ 13.6 (s, $CO_2CH_2CH_3$). $^{31}P\{^1H\}$ (162.0 MHz, $CDCl_3$, 298 K): δ 57.2 (s). ^{31}P (162.0 MHz, $CDCl_3$, 298 K): δ 57.2 (m). ESI-HRMS m/z for $C_{21}H_{35}O_3N_2P$ $[M + H]^+$: calcd 395.2464, 396.2496, 397.2524; found 395.2456, 396.2485, 397.2519. UV-Vis Spectroscopy, 5×10^{-5} M in toluene: 317.02 nm, 0.4018 absorption. IR: ν_{max} (cm^{-1}) 2960.8 (m), 2923.5 (m), 2872.1 (m), 1730.2 (m; C=O).

S1.7. Comparison of “step-wise” vs. “one-pot” synthesis of **3**

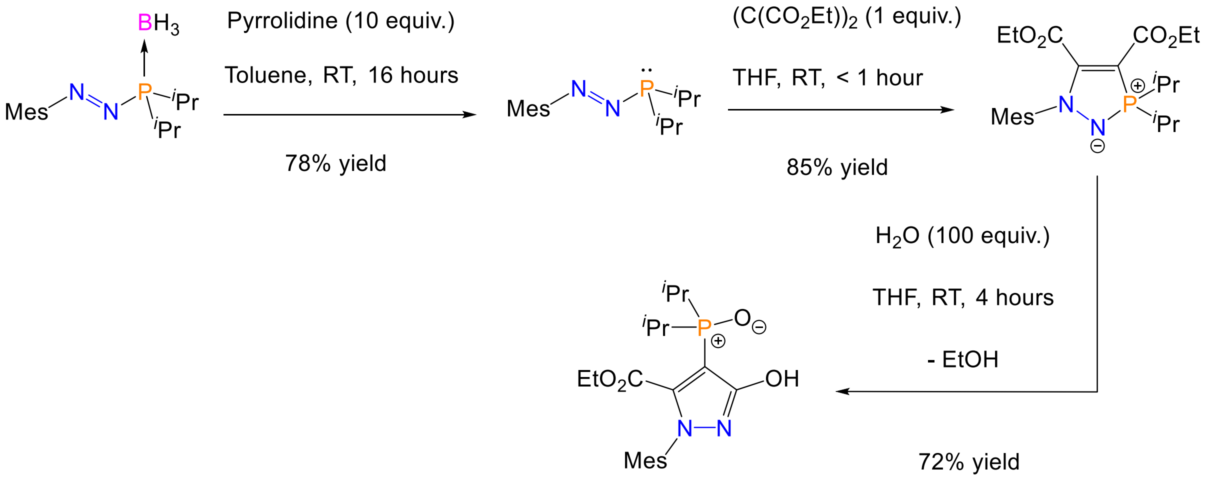

3 **Total over three steps = 48% yield**

*Figure S1. ‘Step-wise’ procedure for the synthesis of **3** from MesN$_2$P^{i}Pr$_2$.BH$_3$.*

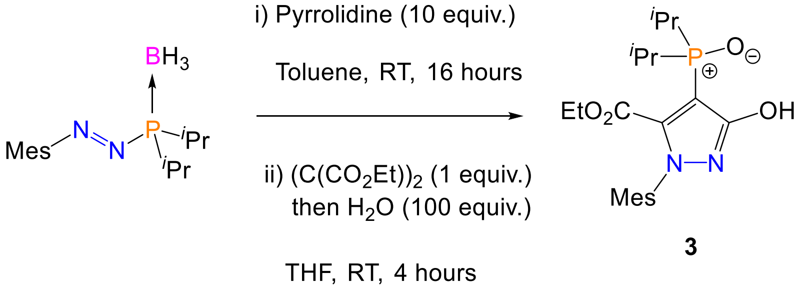

3

'One-pot' procedure = 65% yield

*Figure S2. ‘One-pot’ procedure for the synthesis of **3** from MesN$_2$P^{i}Pr$_2$.BH$_3$.*

For the synthesis of **3** from MesN$_2$P^{i}Pr$_2$.BH$_3$, the procedure could be performed in a ‘step-wise’ manner with full characterisation of each intermediate, outlined as Method D in Section S1.5. Over three individual steps, this gave an overall yield of 48% (individual yields for each step are shown above). Including purification of each intermediate and product, the procedure takes around three days to perform.

Alternatively, **3** could also be synthesised from MesN$_2$P^{i}Pr2.BH$_3$ via a ‘one-pot’ procedure, in which the intermediate products are not isolated nor characterised. This increases the yield from 48% to 65%, and reduces the length of the procedure to no more than two days.

Due to the increased yield and time-efficiency of the ‘one-pot’ procedure over the ‘step-wise’ procedure, only the ‘one-pot’ procedure was used when synthesising **5**. The analogous intermediates were not isolated nor characterised.

S1.8. Attempted reaction of **2** with H_2O

20.0 mg of **2** ($MesN_2P(^tBu_2)_2C_2(CO_2Et)_2$; 0.0432 mmol, 1 equiv.) was dissolved in 2 mL of the solvent of choice (see below) in a round-bottomed flask, in air. 78.0 µL of deionised H_2O (4.32 mmol, 100 equiv.) was then added to this solution via micropipette. The reaction was subsequently left stirring for 2 hours, before the solvent was removed *in vacuo* and the sample analysed by NMR spectroscopy. The temperature was then increased in 20°C increments and the reaction repeated, with stirring for two hours, each time analysed by NMR spectroscopy, until the refluxing temperature of the solvent was reached.

Results:

When THF was used as the solvent, no reaction was seen at any temperature, with only unreacted **2** observed by both ^1H and $^{31}P\{^1H\}$ NMR spectroscopy.

When acetonitrile was used as the solvent, no reaction was seen at any temperature, with only unreacted **2** observed by both ^1H and $^{31}P\{^1H\}$ NMR spectroscopy.

When DMF was used as the solvent, no reaction was seen below 120°C. When the reaction was heated to reflux, NMR showed consumption of **2**, and growth of several new peaks in the $^{31}P\{^1H\}$ NMR spectrum, none of which could be assigned as formation of the analogous pyrazolyl-phosphine oxide; instead these were assigned as degradation of **2** at these elevated temperatures. Furthermore, no luminescence was observed in the sample.

S1.9. Attempted reactions of **1** with other E-H (E = Si, O, N) containing substrates

In the glovebox, 15.0 mg of **1** ($MesN_2P(^iPr_2)_2C_2(CO_2Et)_2$; 0.0345 mmol, 1 equiv.) was dissolved in 1 mL of toluene in a Schlenk flask. 10 equiv. of the desired reagent was then added to the reaction mixture, either as a solid or via micropipette (triethylsilane (55 μL); ethanol (19.5 μL); aniline (31.4 μL); 1,1,1,3,3,3-hexafluoro-2-propanol (36.3 μL); 4-*tert*-butylaniline (55.0 μL); 2,3,4,5,6-pentafluoroaniline (63.2 mg); 4-nitroaniline (47.7 mg)). The reactions were left stirring for two days at 60°C, before the solvent was removed in vacuo and the material analysed by both ^1H and $^{31}P\{^1H\}$ NMR spectroscopy. In all cases, no reaction was observed; NMR spectra showed only known shifts for the starting materials.

Reaction with NH_3:

A J-Young NMR tube was charged with a solution of 10.0 mg of **1** ($MesN_2P(^iPr_2)_2C_2(CO_2Et)_2$; 0.0230 mmol) in 0.6 mL of d_8-THF. This J-Young NMR tube was then degassed on the Schlenk line via two freeze-pump-thaw cycles, and while under static vacuum, backfilled with an atmosphere of NH_3. This sample was then monitored by ^1H and $^{31}P\{^1H\}$ NMR spectroscopy for two days, after which time no reaction was observed. The J-Young NMR tube was then placed in a 60°C sand bath for two further days; no reaction was observed after this time by either ^1H, $^{31}P\{^1H\}$ or ^{31}P NMR spectroscopy, with only starting materials present. A mass spectrum, taken from an aliquot of the reaction mixture, also showed only **1** to be present.

S2. Photophysical properties of pyrazolyl-phosphine oxides

S2.1. General Information

All UV/Vis absorption spectra were collected on an Agilent Cary60 UV/Vis Spectrometer, and samples irradiated in a quartz glass High Precision Cell cuvette (10 x 10 mm) from Hellma Analytics. Absorption wavelengths are reported in nm. Before running all samples, a background reading was run (with blank solvent, and no sample present), with the values from this background readings subsequently subtracted from the actual sample. All solvents used were of spectrometric grade.

All emission spectra were collected on a Cary Eclipse Fluorescence Spectrophotometer, using a Xenon flash lamp (80 Hz) as the light source, and samples irradiated in a synthetic quartz glass Micro Fluorescence cuvette (10 x 10 mm) from Thorlabs. Unless otherwise stated, the excitation and emission slit widths were kept at 5 nm and the photon multiplier voltage at 600 V. Excitation wavelengths were varied depending on the solvent and compound used; see further details below. Absolute fluorescence quantum yields were determined with an Edinburgh FLS1000 Photoluminesence Spectrometer, calibrated with an integrating sphere system, and samples irradiated in a synthetic quartz glass Micro Fluorescence cuvette (10 x 10 mm) from Thorlabs. All solvents used were of spectrometric grade.

Commercial reagents were purchased, suitably stored as specified by the supplier, and used as received, unless otherwise stated, from Sigma-Aldrich (zinc chloride anhydrous, > 98%; calcium chloride anhydrous, > 98%; sodium chloride, > 99%; potassium chloride anhydrous, > 99%; manganese (II) chloride tetrahydrate, > 98%; chromium (III) chloride hexahydrate, > 98%), ThermoScientific (copper (II) chloride anhydrous, 99%; iron (II) chloride anhydrous, 99.5%, lanthanum (III) chloride heptahydrate, 99%; lithium chloride anhydrous, 99%), Honeywell (aluminium chloride hexahydrate, 99%), Fluorochem (magnesium chloride anhydrous, 99%) or Alfa Aesar (iron (III) chloride anhydrous, 98%).

S2.2. UV/Vis and Fluorescence Spectra for pyrazolyl-phosphine oxides

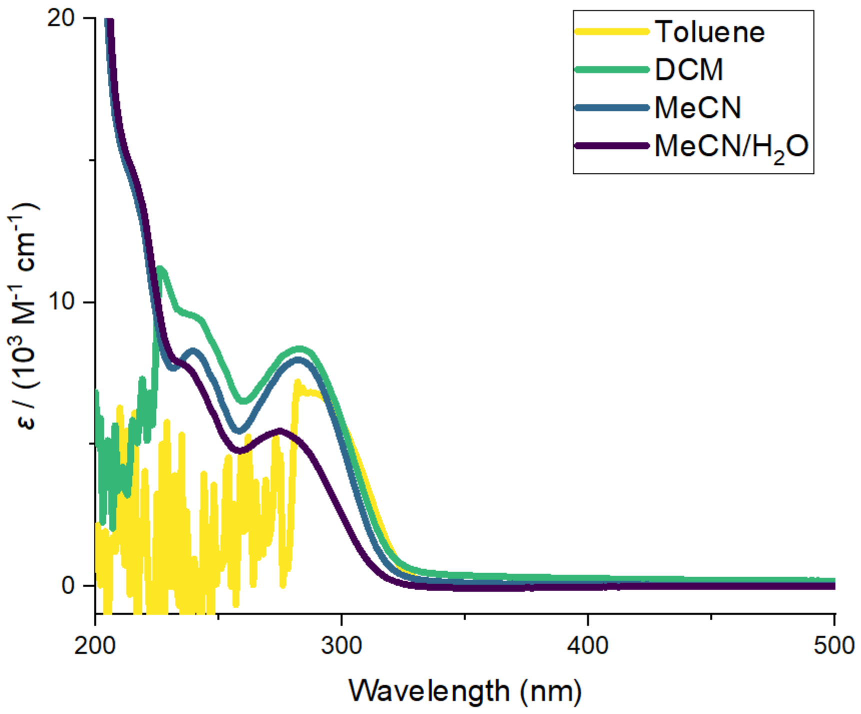

*Figure S3. UV/Vis spectra of **3** (50 μM, solvent for each sample shown above).*

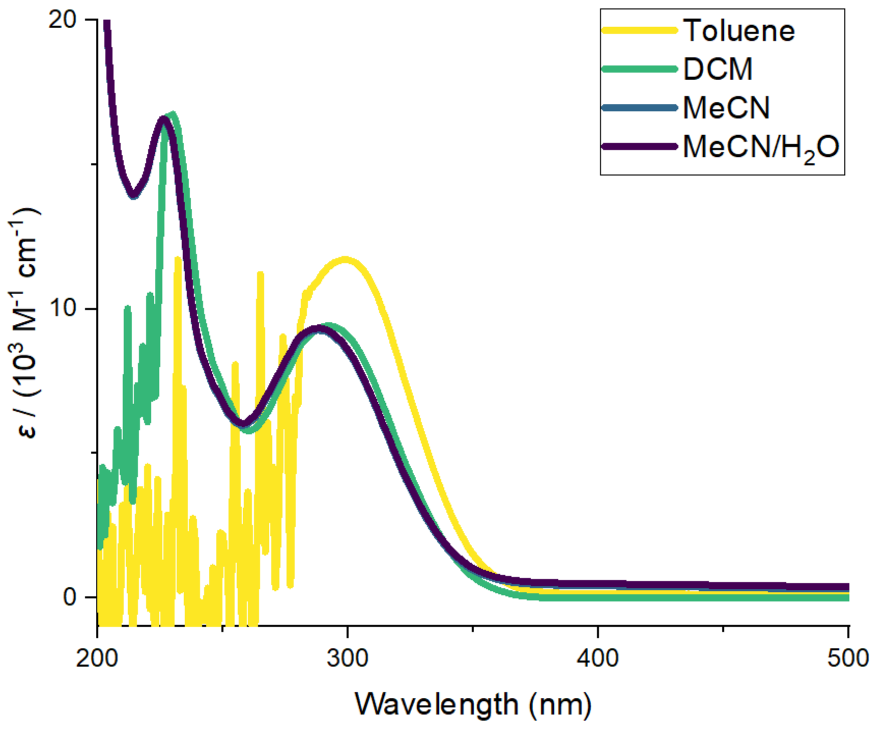

*Figure S4. UV/Vis spectra of **5** (50 μM, solvent for each sample shown above).*

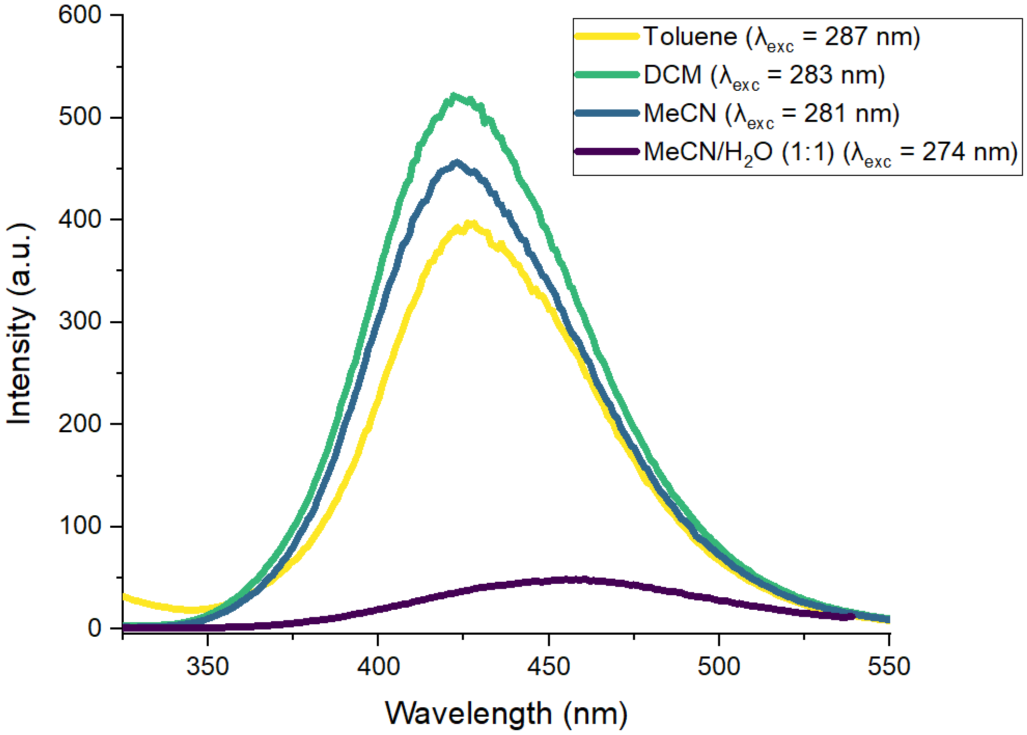

*Figure S5. Fluorescence spectra of **3** (5 μM, solvent and excitation wavelength for each sample shown above).*

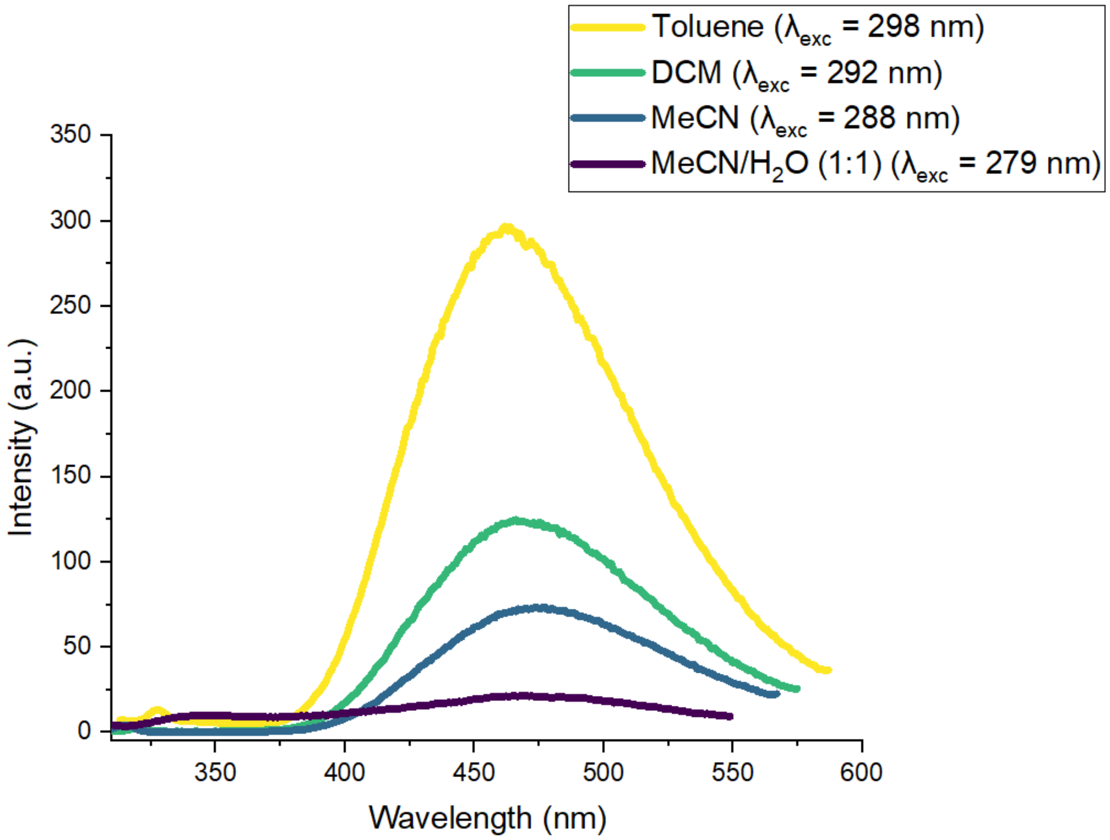

*Figure S6. Fluorescence spectra of **5** (5 μM, solvent and excitation wavelength for each sample shown above).*

*Table S1. Photophysical properties of pyrazolyl-phosphine oxides **3** and **5**.*

Compound	Solvent	λ_{abs} [A] (nm)	ε[A] ($10^3.M^{-1}.cm^{-1}$)	λ_{em}(B) (nm)	Stokes shift (nm)	Maximum fluorescence intensity(B) (a.u.)	Φ_F[C]
**3**	Toluene	287	6.84	426	139	397.3	0.89
**3**	DCM	283	8.37	422	139	521.8	0.40
**3**	MeCN	281	7.96	423	142	456.7	0.24
**3**	MeCN/H_2O (1:1)	274	5.46	461	187	48.8	0.12
**5**	Toluene	298	11.7	462	164	297.1	0.36
**5**	DCM	292	9.42	466	174	125.0	0.089
**5**	MeCN	288	9.26	470	182	73.0	0.043
**5**	MeCN/H_2O (1:1)	279	8.83	468	189	21.7	0.029

[A] = Taken at 50 μM.

[B] = Taken at 5 μM. For each sample, $\lambda_{abs} = \lambda_{exc}$.

[C] = Taken at 50 μM. For each sample, $\lambda_{abs} = \lambda_{exc}$.

S2.3. Metal-ion sensing with pyrazolyl-phosphine oxides

*Table S2. F/F_0 ratios for **3** (50 μM) in the presence of various metal ions (5 equiv.) in MeCN/H_2O (1:1). λ_{exc} = 274 nm.*

Metal ion[A]	Fluorescence intensity [a.u] in the presence of M^{n+} (5 equiv.)	F/F_0 ratio[B]	Standard Deviation[B]
Free **3** (0 eq. M^{n+})	369 *(50 μM)*<br>86.0 *(10 μM)*	1.00<br>1.00	0.015<br>0.057
Li^+	375	1.02	0.011
Na^+	372	1.00	0.015
K^+	374	1.00	0.016
Mg^{2+}	373	1.01	0.011
Ca^{2+}	366	1.00	0.032
Fe^{2+} [C]	248	0.721	0.043
Fe^{3+}	8.06	0.0276	0.0089
Cr^{3+}	373	1.01	0.0040
Mn^{2+}	362	0.968	0.030
Cu^{2+}	253	0.657	0.023
Zn^{2+}	417	1.08	0.030
La^{3+}	365	1.09	0.089
Al^{3+} [D]	315	3.71	0.10

[A] = All metals used as chlorides, i.e. LiCl, NaCl etc. Concentration of metal ions is 250 μM unless otherwise stated.

[B] = F/F_0 ratios and standard deviations are calculated across three independent measurements.

[C] = Anhydrous $FeCl_2$ was stored in the glovebox when not in use. For measurements, a sample of $FeCl_2$ was removed from the glovebox and made up to the appropriate concentration in the chosen solvent. The measurement was then performed immediately.

[D] = Concentration of Al^{3+} = 50 μM (5 equiv.). Concentration of **3** = 10 μM.

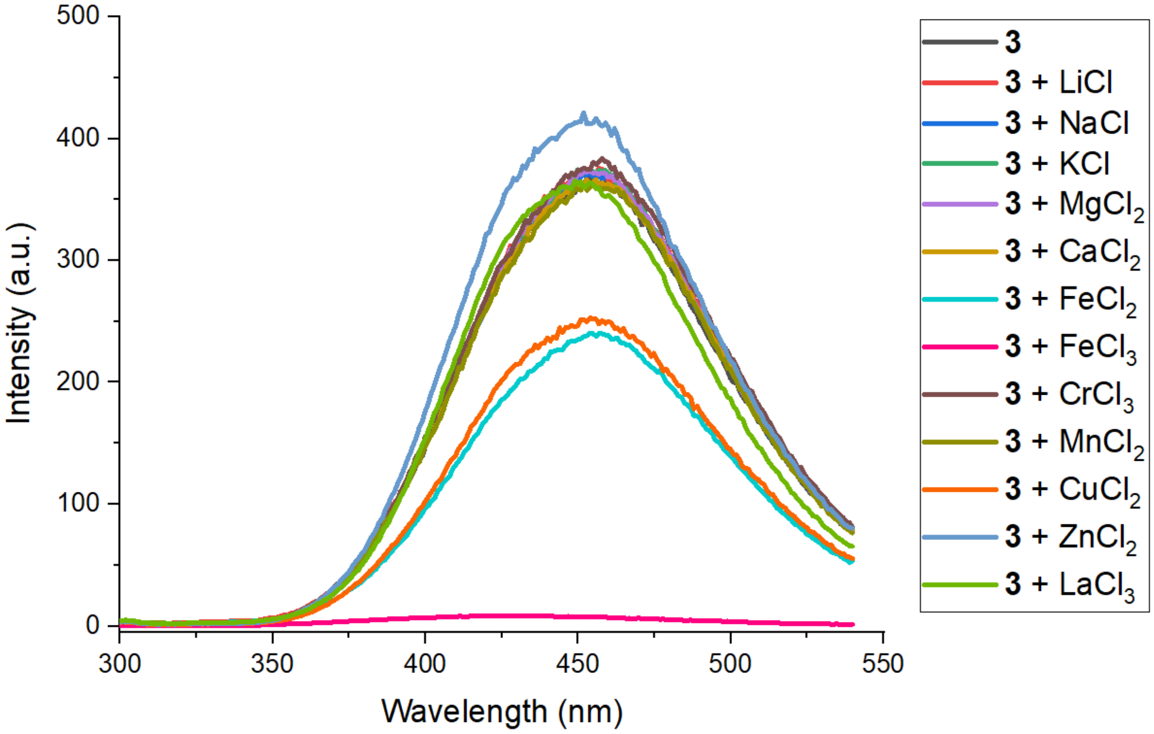

*Figure S7. Fluorescence spectra of **3** (50 μM), in MeCN/H_2O (1:1), in the presence of various metal salts (5 equiv., 250 μM). λ_{exc} = 274 nm.*

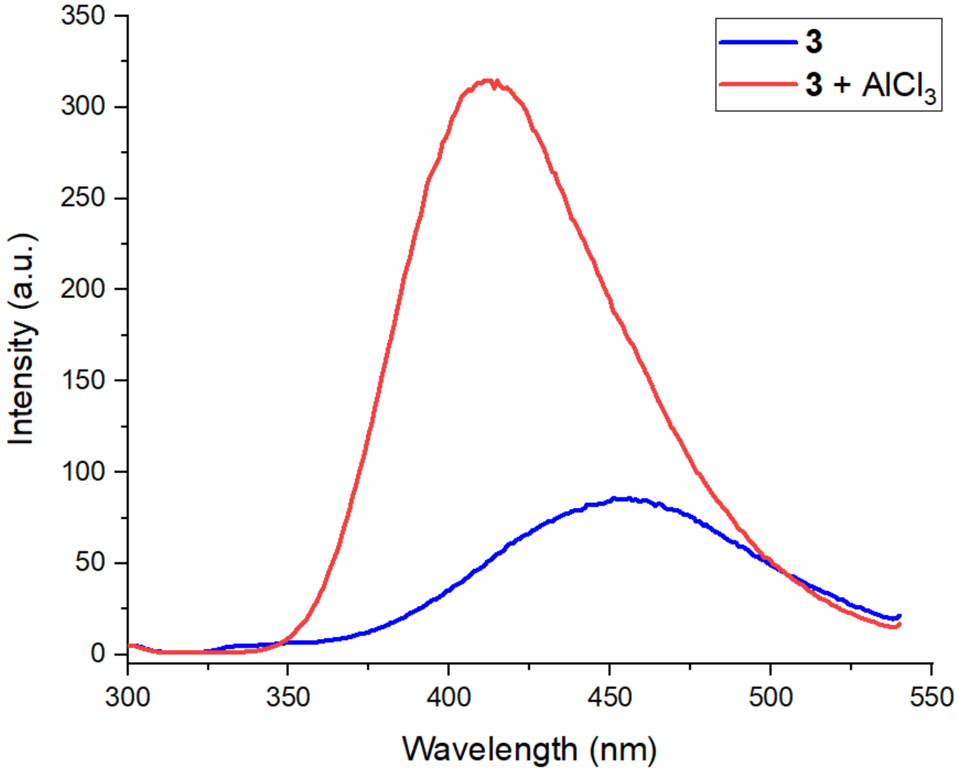

*Figure S8. Fluorescence spectra of **3** (10 µM), in MeCN/H_2O (1:1), in the presence of $AlCl_3$ (5 equiv., 50 µM). λ_{exc} = 274 nm.*

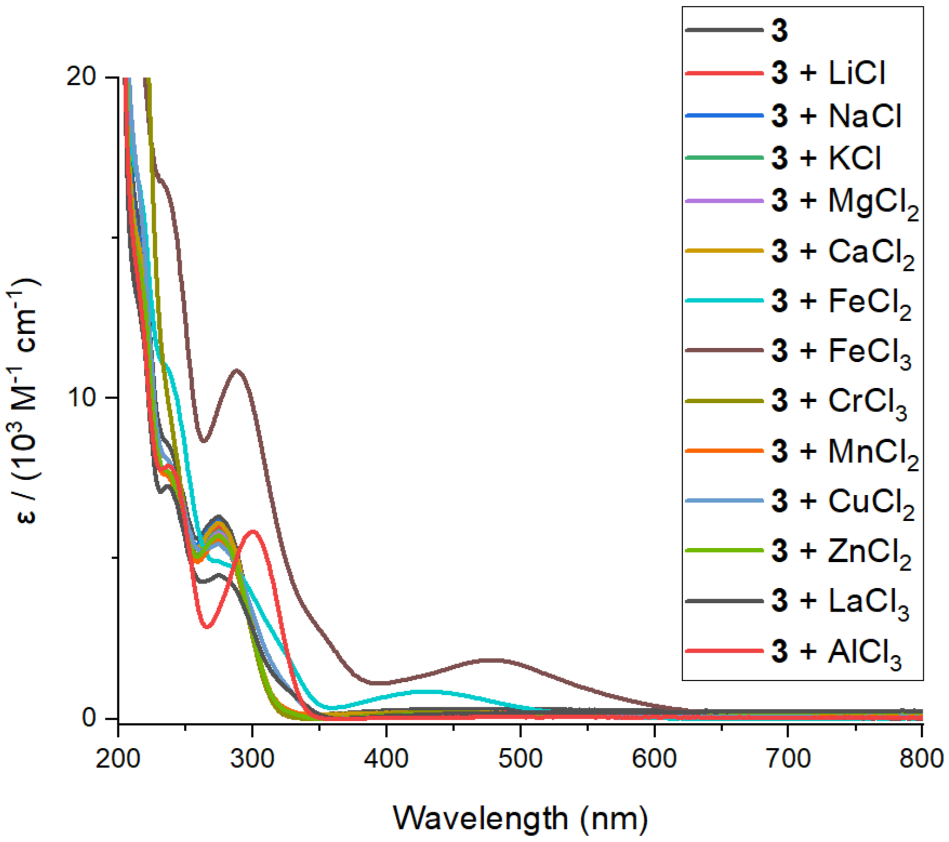

*Figure S9. UV/Vis spectra of **3** (50 µM), in MeCN/H_2O (1:1), in the presence of various metal salts (5 equiv., 250 µM).*

*Table S3. F/F_0 ratios for **5** (50 μM) in the presence of various metal ions (5 equiv.) in MeCN/H_2O (1:1). λ_{exc} = 279 nm.*

**Metal ion[A]**	**Fluorescence intensity [a.u] in the presence of M^{n+} (5 equiv.)**	**F/F_0 ratio[B]**	**Standard Deviation[B]**
Free **5** (0 eq. M^{n+})	145	1.00	0.026
Li^+	144	0.984	0.013
Na^+	145	0.999	0.041
K^+	139	0.957	0.012
Mg^{2+}	139	0.961	0.012
Ca^{2+}	145	0.989	0.013
Fe^{2+} [C]	112	0.778	0.030
Fe^{3+}	13.9	0.0727	0.020
Cr^{3+}	151	1.02	0.0078
Mn^{2+}	139	0.935	0.0062
Cu^{2+}	106	0.717	0.010
Zn^{2+}	143	0.971	0.016
La^{3+}	144	0.987	0.011
Al^{3+}	199	1.37	0.069

[A] = All metals used as chlorides, i.e. LiCl, NaCl etc. Concentration of metal ions is 250 μM unless otherwise stated.

[B] = F/F_0 ratios and standard deviations are calculated across three independent measurements.

[C] = Anhydrous $FeCl_2$ was stored in the glovebox when not in use. For measurements, a sample of $FeCl_2$ was removed from the glovebox and made up to the appropriate concentration in the chosen solvent. The measurement was then performed immediately.

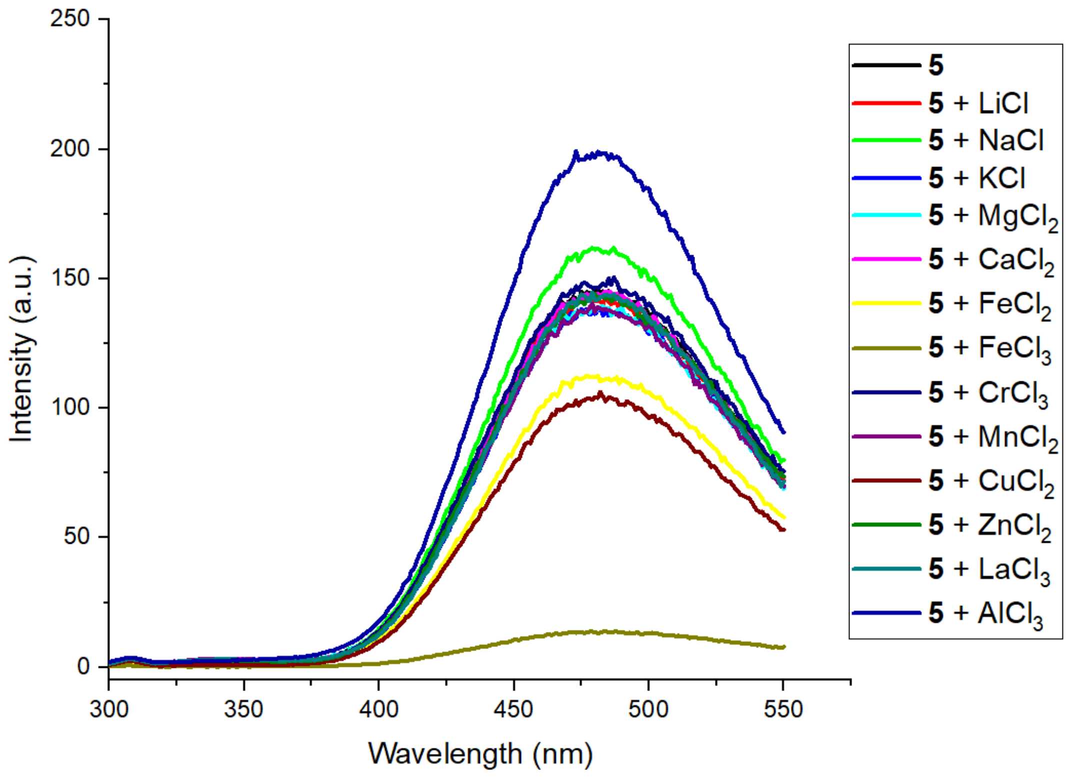

Figure S10. Fluorescence spectra of ***5*** *(50 µM), in MeCN/H_2O (1:1), in the presence of various metal salts (5 equiv., 250 µM).* λ_{exc} *= 279 nm.*

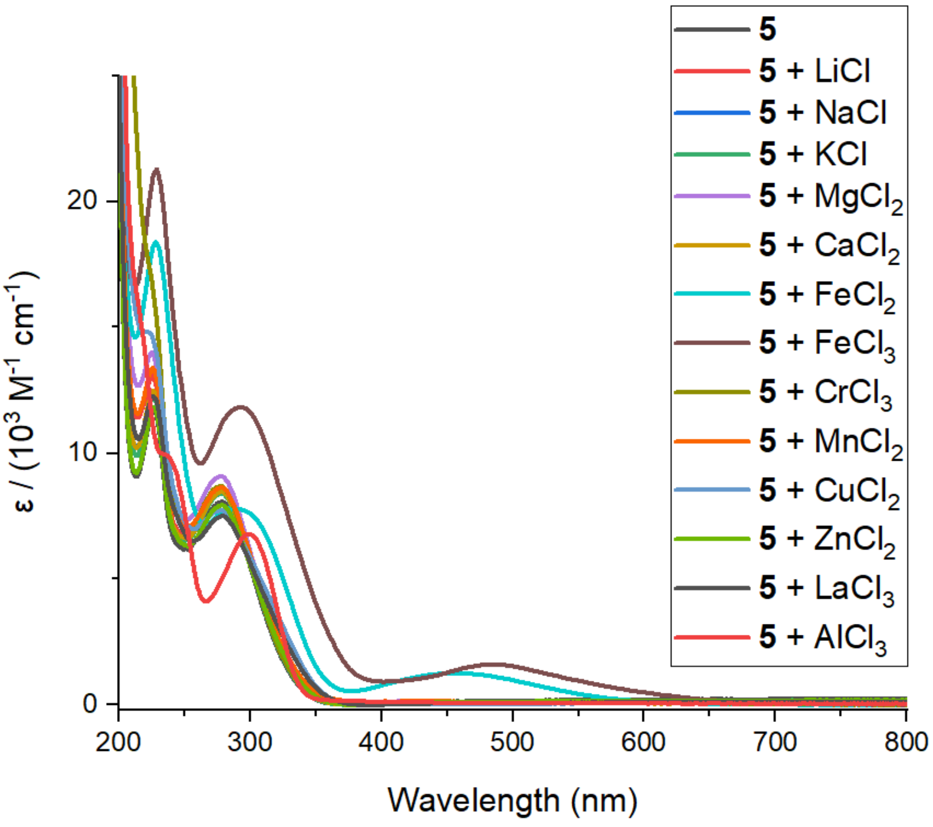

Figure S11. UV/Vis spectra of ***5*** *(50 µM), in MeCN/H_2O (1:1), in the presence of various metal salts (5 equiv., 250 µM).*

S2.4. Titrations of **3** and **5** with $FeCl_3$

*Titration of **3** with $FeCl_3$ via fluorescence spectroscopy:*

The initial concentration of **3** was 50 µM in MeCN/H_2O (1:1). Solutions of $FeCl_3$ in the same solvent system were then added to the solution of **3**, such that the concentration of **3** remained 50 µM, while the concentration of $FeCl_3$ is increased from 0 µM to 400 µM in 23 steps.

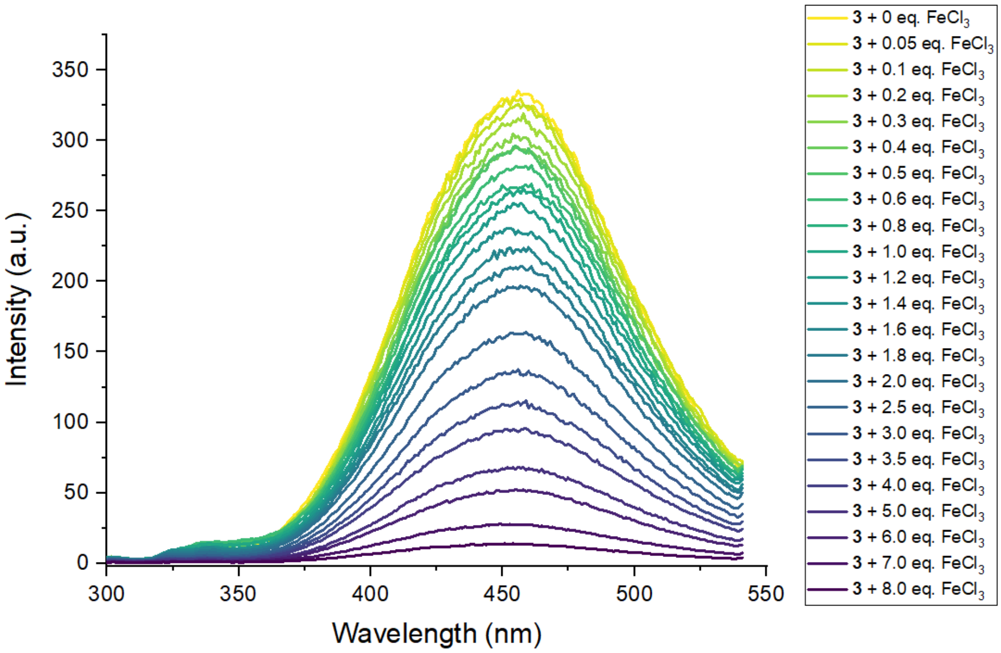

*Figure S12. Fluorescence spectra of **3** (50 µM), in MeCN/H_2O (1:1), with $FeCl_3$ (0 - 8 equiv.). λ_{exc} = 274 nm.*

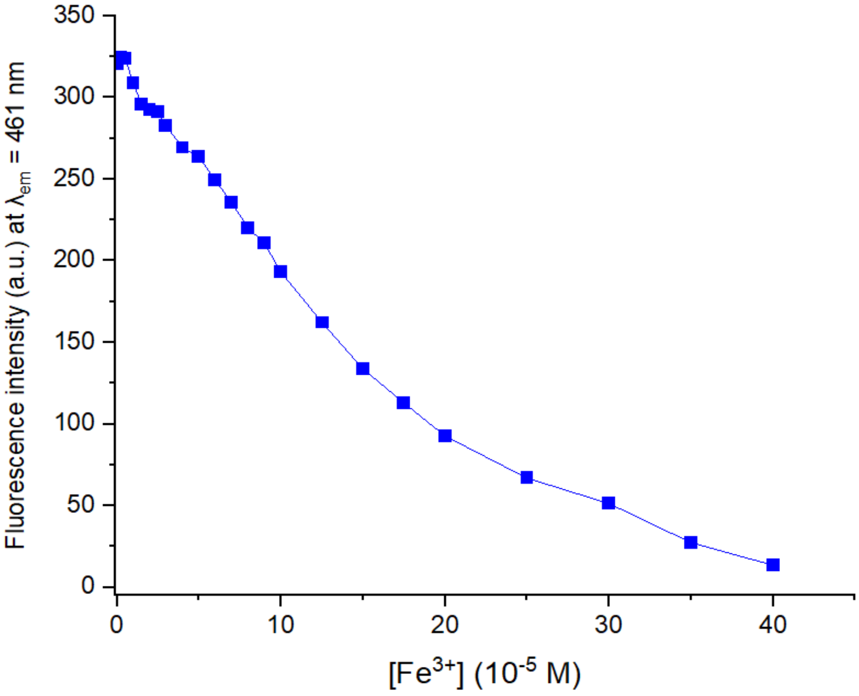

*Figure S13. Fluorescence intensities (a.u.) of **3** (50 µM), in MeCN/H_2O (1:1), with $FeCl_3$ (0 - 8 equiv.). For all data points, λ_{exc} = 274 nm, λ_{em} = 461 nm.*

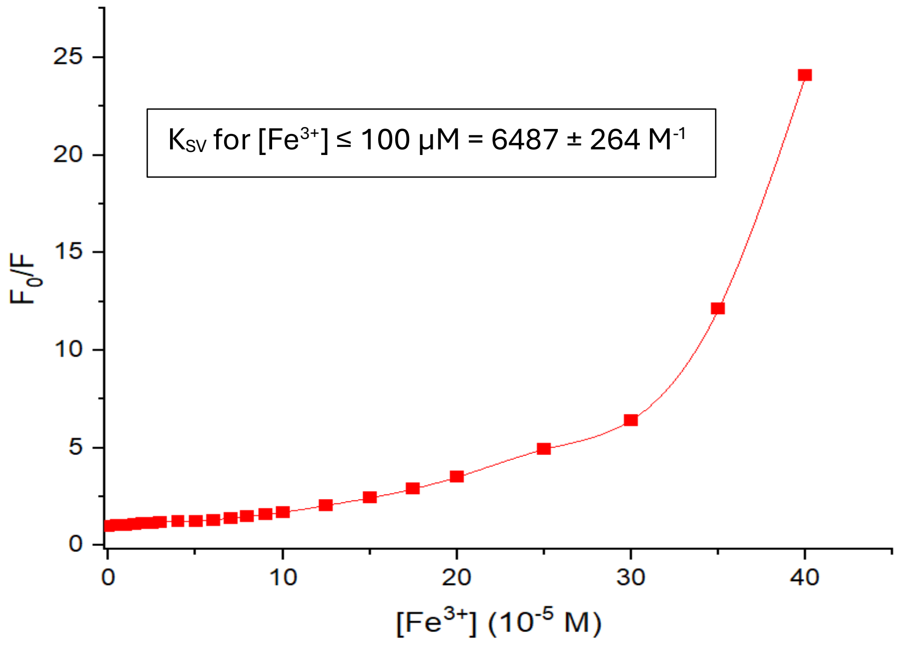

*Figure S14. Stern-Volmer plot of **3** (50 µM), in MeCN/H_2O (1:1), with $FeCl_3$ (0 - 8 equiv.). For all data points, λ_{exc} = 274 nm, λ_{em} = 461 nm. K_{SV} was calculated based on the linear section of the plot (≤ 100 µM).*

*Titration of **3** with $FeCl_3$ via UV/Vis spectroscopy:*

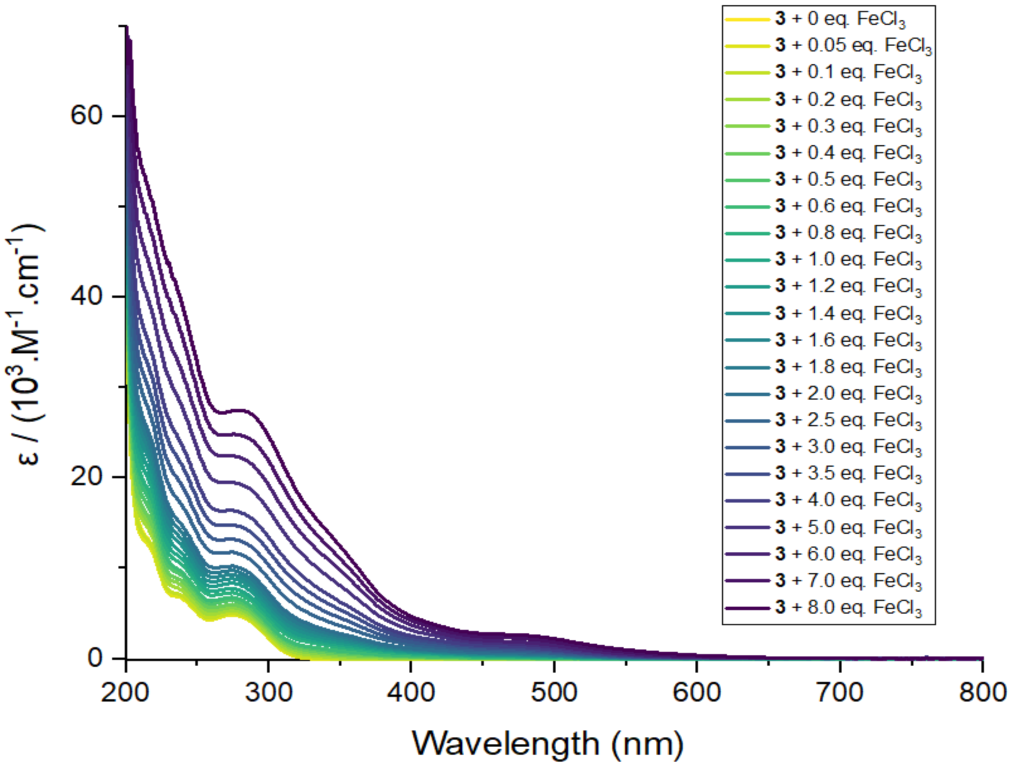

*Figure S15. UV/Vis spectra of **3** (50 µM), in MeCN/H_2O (1:1), with $FeCl_3$ (0 - 8 equiv.).*

*Titration of **5** with $FeCl_3$ via fluorescence spectroscopy:*

The initial concentration of **5** was 50 μM in MeCN/H_2O (1:1). Solutions of $FeCl_3$ in the same solvent system were then added to the solution of **5**, such that the concentration of **5** remained 50 μM, while the concentration of $FeCl_3$ is increased from 0 μM to 400 μM in 23 steps.

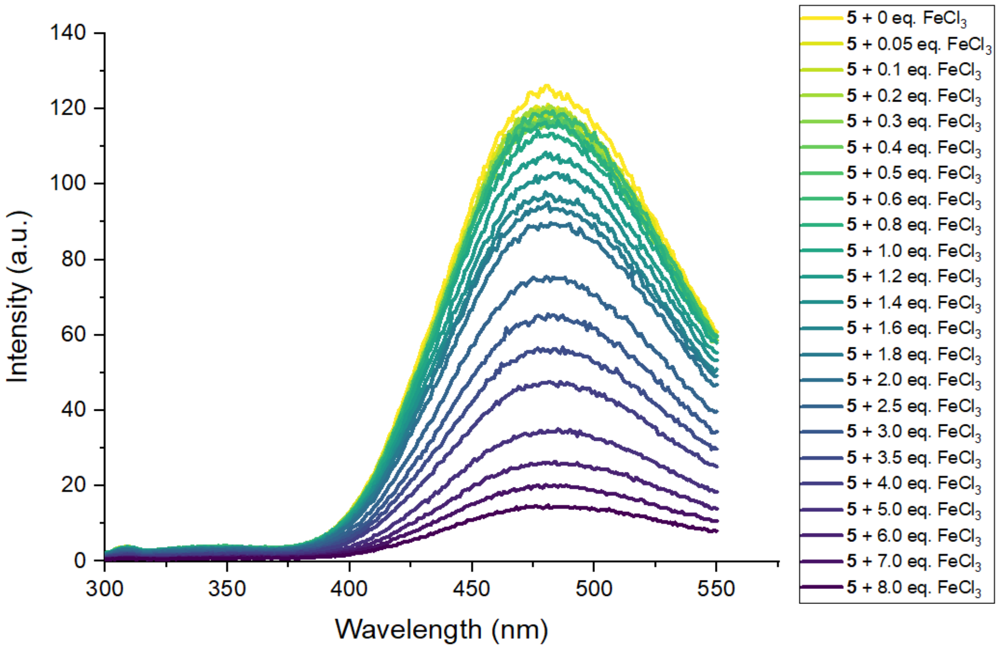

*Figure S16. Fluorescence spectra of **5** (50 μM), in MeCN/H_2O (1:1), with $FeCl_3$ (0 - 8 equiv.). λ_{exc} = 279 nm.*

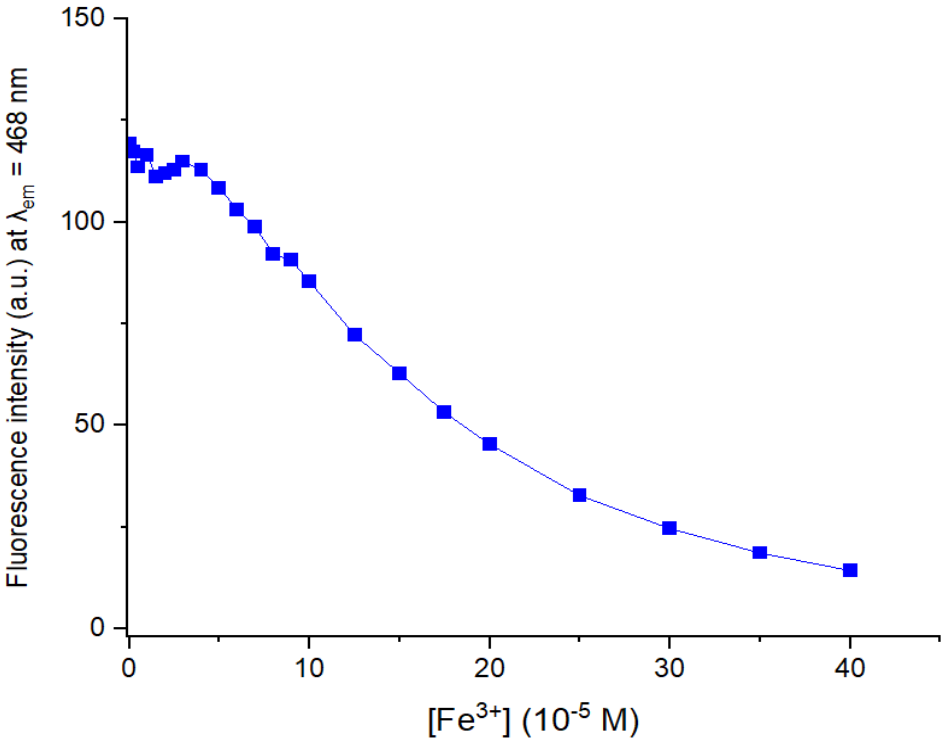

*Figure S17. Fluorescence intensities (a.u.) of **5** (50 μM), in MeCN/H_2O (1:1), with $FeCl_3$ (0 - 8 equiv.). For all data points, λ_{exc} = 279 nm, λ_{em} = 468 nm.*

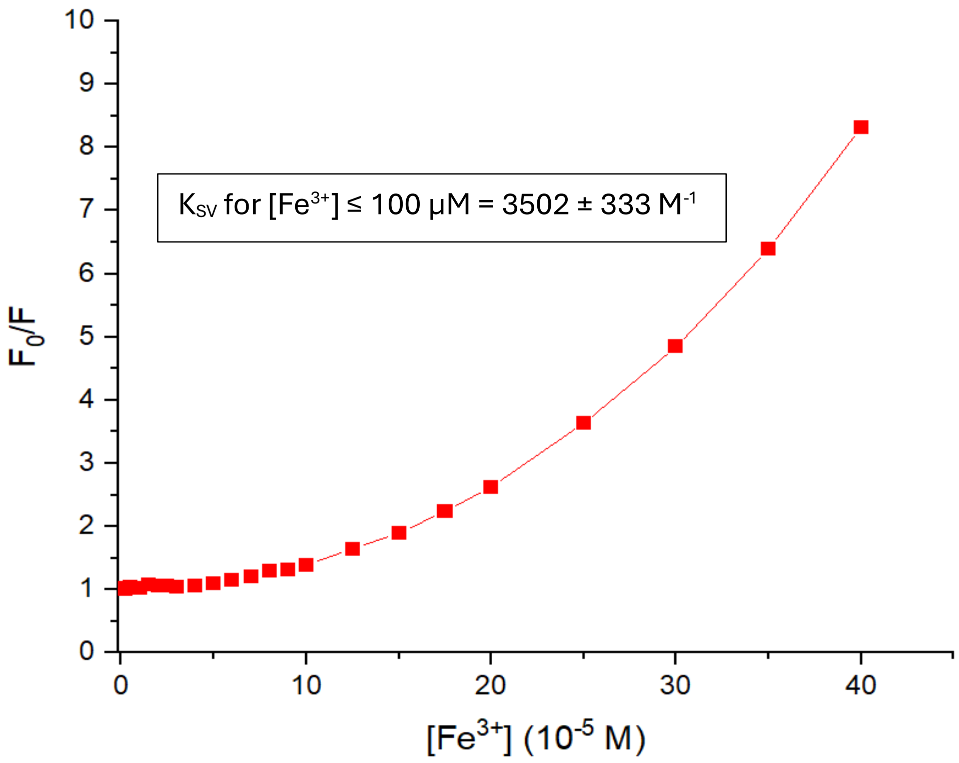

*Figure S18. Stern-Volmer plot of **5** (50 µM), in MeCN/H_2O (1:1), with $FeCl_3$ (0 - 8 equiv.). For all data points, λ_{exc} = 279 nm, λ_{em} = 468 nm. K_{SV} was calculated based on the linear section of the plot (≤ 100 µM).*

*Titration of **5** with $FeCl_3$ via UV/Vis spectroscopy:*

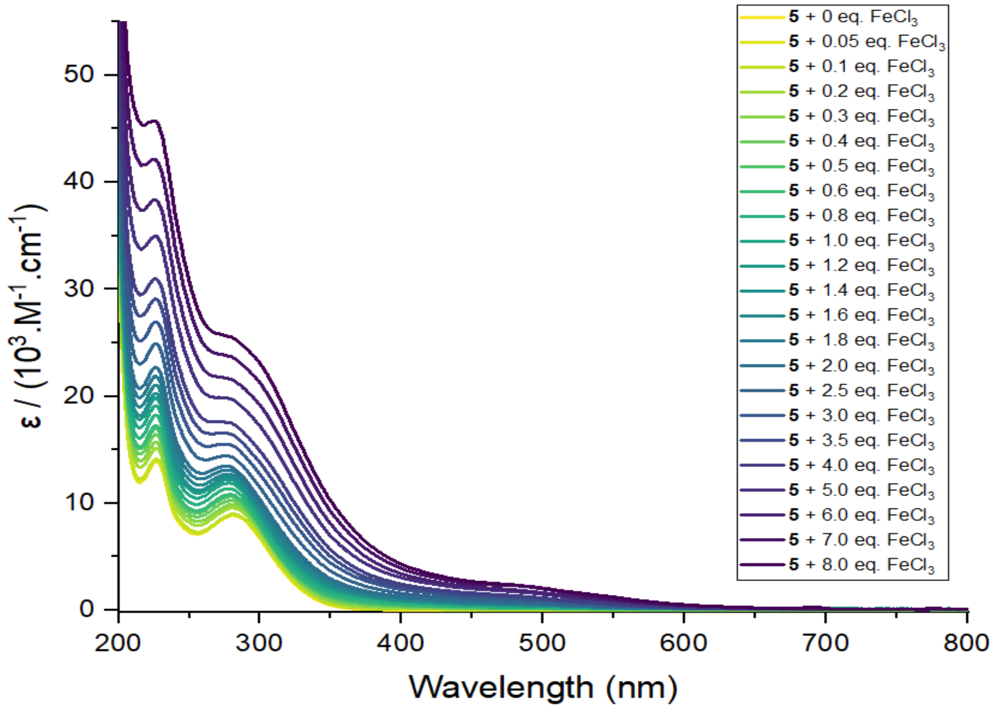

*Figure S19. UV/Vis spectra of **5** (50 µM), in MeCN/H_2O (1:1), with $FeCl_3$ (0 - 8 equiv.).*

S2.5. Titrations of **3** with $AlCl_3$

*Titration of **3** with $AlCl_3$ via fluorescence spectroscopy:*

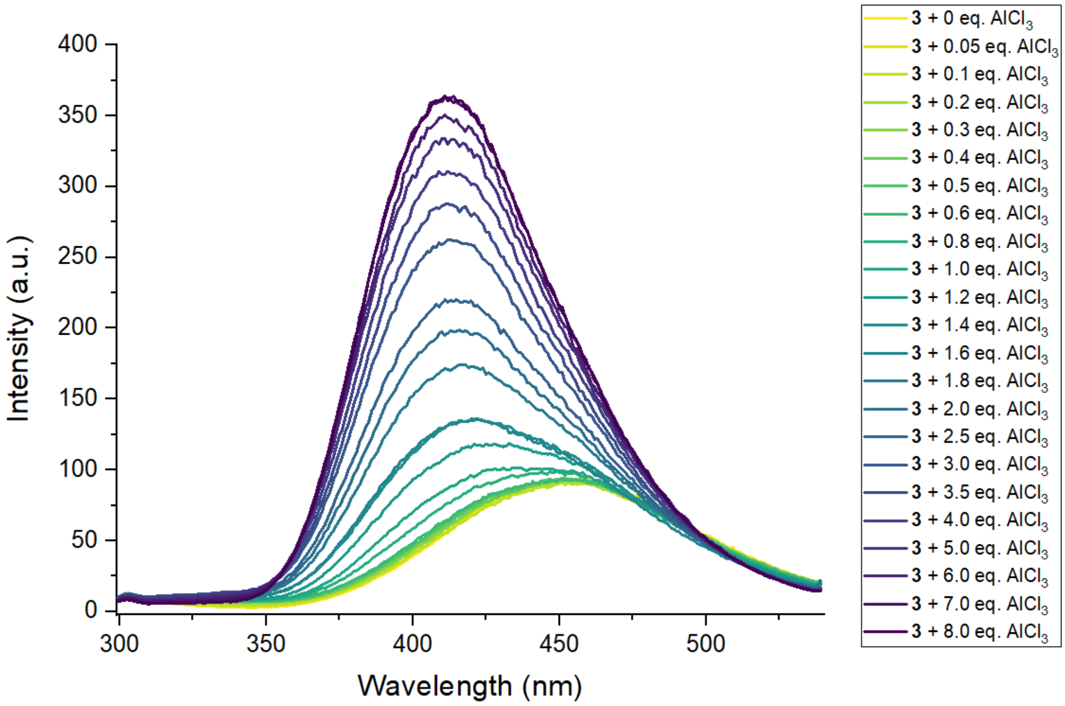

*Figure S20. Fluorescence spectra of **3** (10 μM), in MeCN/H_2O (1:1), with $AlCl_3$ (0 - 8 equiv.). λ_{exc} = 274 nm.*

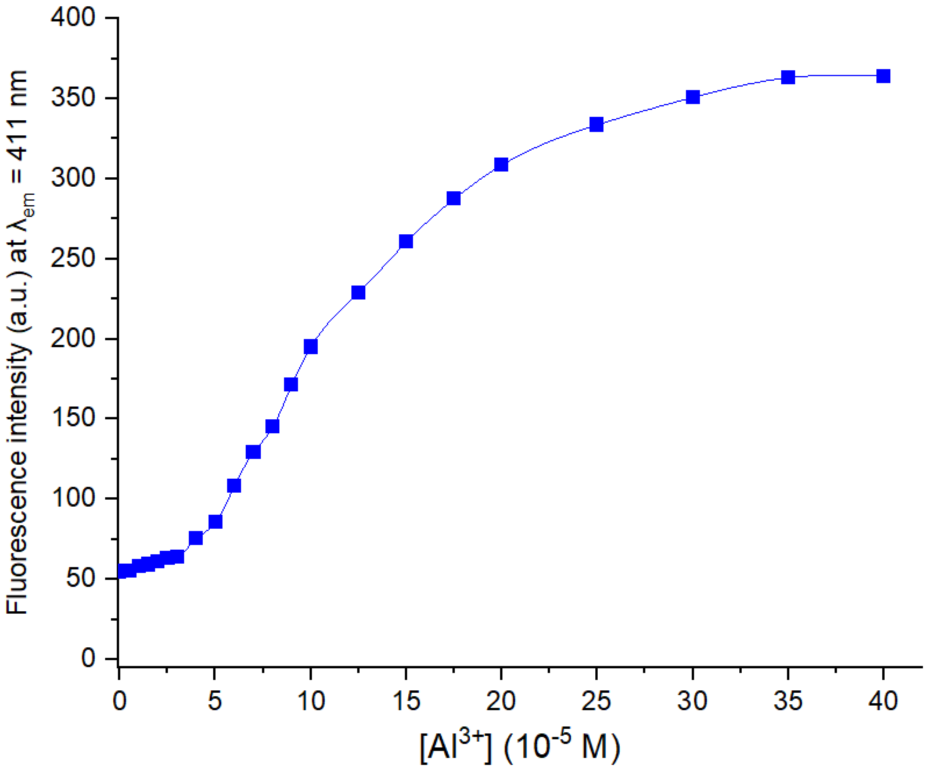

*Figure S21. Fluorescence intensities (a.u.) of **3** (10 μM), in MeCN/H_2O (1:1), with $AlCl_3$ (0 - 8 equiv.). For all data points, λ_{exc} = 274 nm, λ_{em} = 411 nm.*

*Titration of **3** with $AlCl_3$ via UV/Vis spectroscopy:*

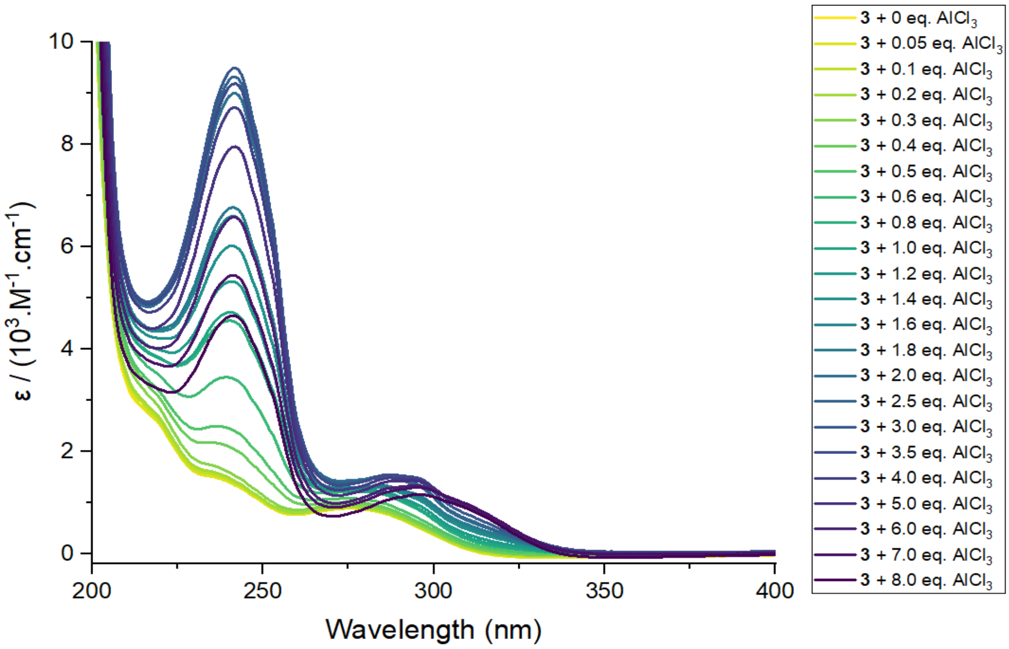

*Figure S22. UV/Vis spectra of **3** (50 μM), in MeCN/H_2O (1:1), with $AlCl_3$ (0 - 8 equiv.).*

S2.6. Job Plots for **3** with $FeCl_3$ and $AlCl_3$

*Job Plot of **3** with $FeCl_3$ via fluorescence spectroscopy*

A series of samples containing varying proportions of **3** and $FeCl_3$ in MeCN/H_2O (1:1) were prepared. The total concentration of **3** and $FeCl_3$ were kept at 50 μM for all measurements. For all data points shown below, λ_{exc} = 274 nm, λ_{em} = 461 nm.

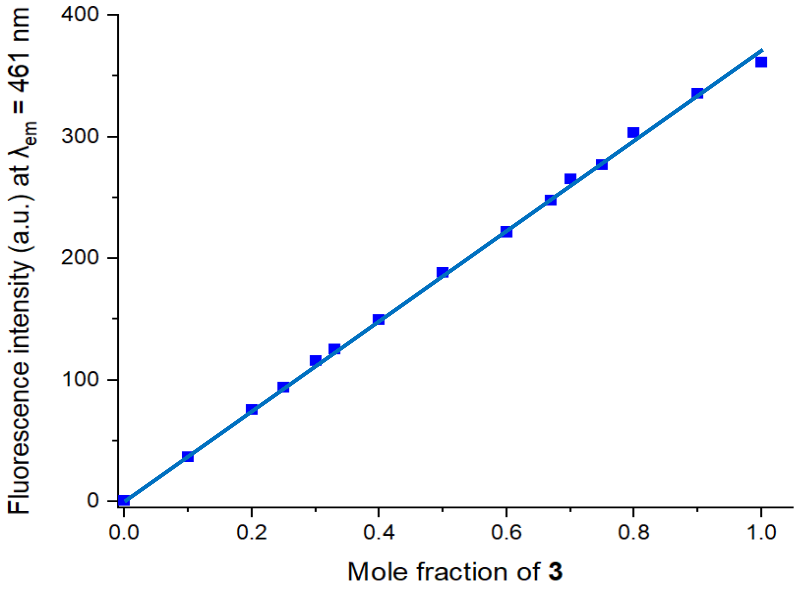

*Figure S23. Job plot for varying mole fractions of **3** and $FeCl_3$. Total concentration for all readings was kept at 50 μM.*

*Job Plot of **3** with $AlCl_3$ via fluorescence spectroscopy*

A series of samples containing varying proportions of **3** and $AlCl_3$ in $MeCN/H_2O$ (1:1) were prepared. The total concentration of **3** and $AlCl_3$ were kept at 50 μM for all measurements. For all data points shown below, λ_{exc} = 274 nm, λ_{em} = 411 nm.

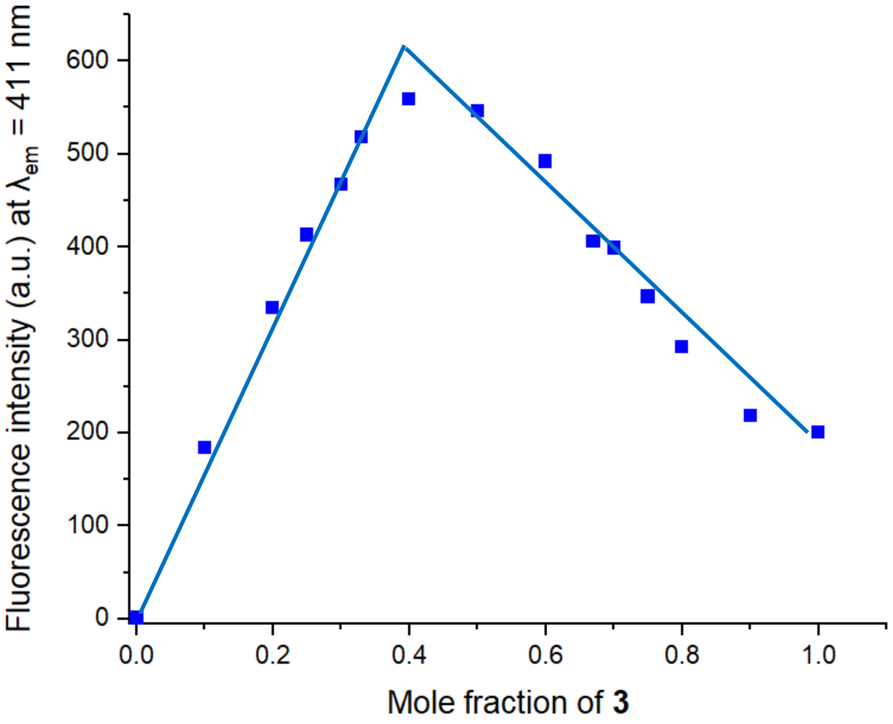

*Figure S24. Job plot for varying mole fractions of **3** and $AlCl_3$. Total concentration for all readings was kept at 50 μM.*

S2.7. Selectivity studies for **3** with $FeCl_3$

*Titration of **3** with $FeCl_3$ via fluorescence spectroscopy in the presence of 1 equiv. of other metal ions*

The initial concentration of **3** was 50 μM in MeCN/H_2O (1:1). Solutions of 12 different metal salt solutions (all 1 equiv.; Li^+, Na^+, K^+, Mg^{2+}, Ca^{2+}, Cr^{3+}, Mn^{2+}, Fe^{2+}, Cu^{2+}, Zn^{2+}, Al^{3+}, La^{3+}) were then added so that the overall concentration of **3** and the 12 metals in solution were 50 μM. Solutions of $FeCl_3$ in the same solvent system were then added to the solution of **3**, such that the concentration of **3** and the 12 other metals remained 50 μM, while the concentration of $FeCl_3$ is increased from 0 μM to 350 μM in 14 steps.

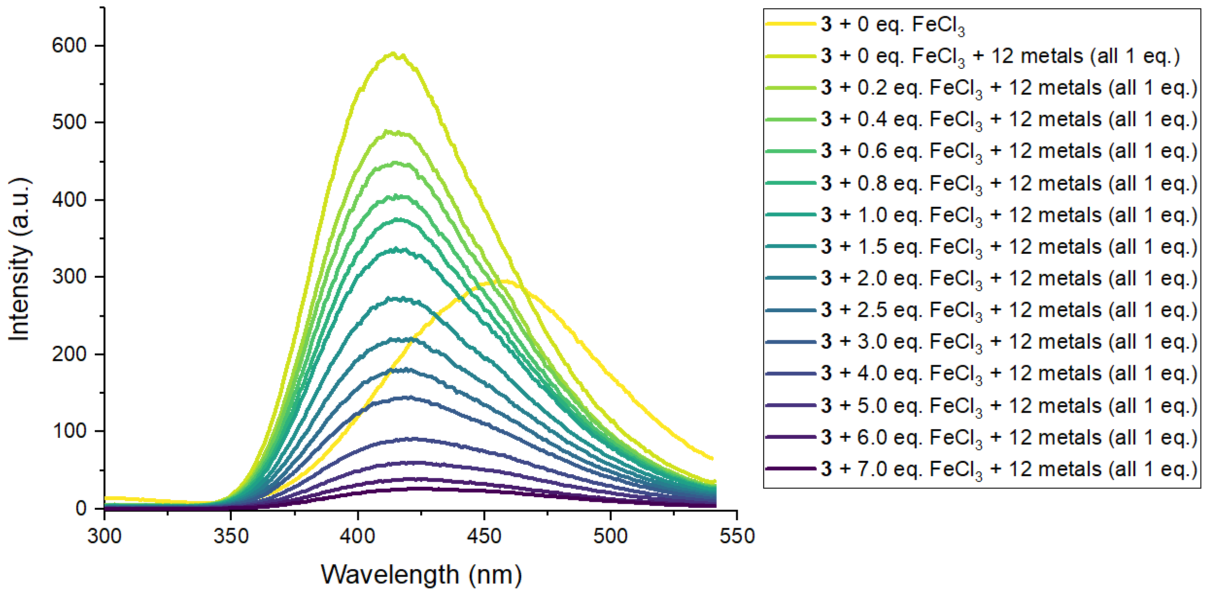

*Figure S25. Fluorescence spectra of **3** (50 μM), in MeCN/H_2O (1:1), with 1 equiv. of other 12 metal salts, and $FeCl_3$ (0 - 7 equiv.). λ_{exc} = 274 nm.*

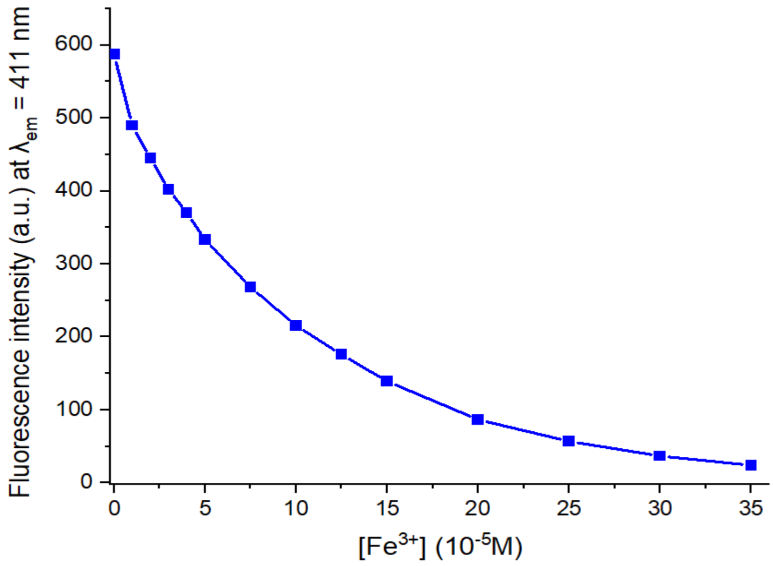

*Figure S26. Fluorescence intensities (a.u.) of **3** (50 μM), in MeCN/H_2O (1:1), with 1 equiv. of other 12 metal salts, and $FeCl_3$ (0 - 7 equiv.). For all data points, λ_{exc} = 274 nm, λ_{em} = 411 nm.*

*Titration of **3** with $FeCl_3$ via fluorescence spectroscopy with increasing concentrations of other metal ions*

The initial concentration of **3** was 50 µM in MeCN/H_2O (1:1). Solutions of 13 different metal salt solutions (Li^+, Na^+, K^+, Mg^{2+}, Ca^{2+}, Cr^{3+}, Mn^{2+}, Fe^{2+}, Cu^{2+}, Zn^{2+}, Al^{3+}, La^{3+}, **and** Fe^{3+}) in the same solvent system were then added to the solution of **3**, such that the concentration of **3** remained 50 µM, while the concentration of all other metals is increased from 0 µM to 350 µM in 14 steps.

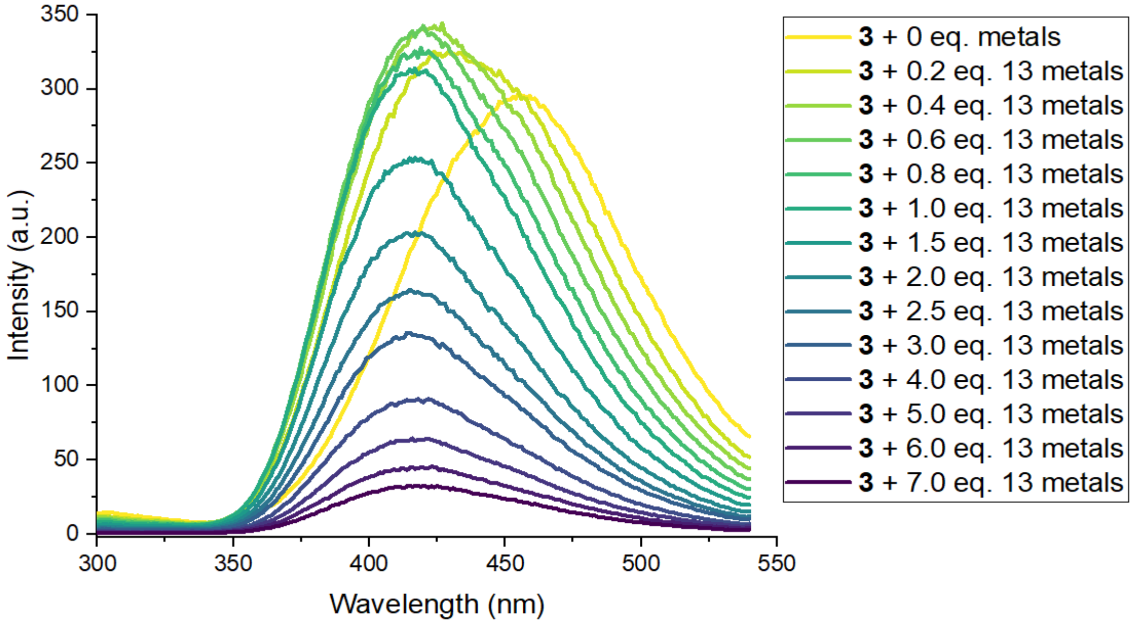

*Figure S27. Fluorescence spectra of **3** (50 µM), in MeCN/H_2O (1:1), with increasing concentrations of 13 metal salts (0 - 7 equiv.). λ_{exc} = 274 nm.*

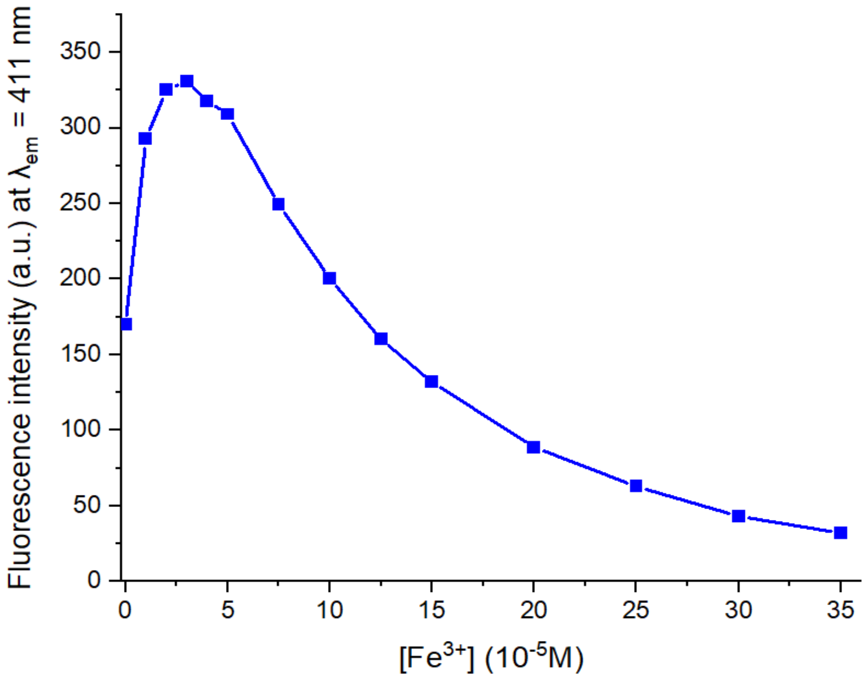

*Figure S28. Fluorescence intensities (a.u.) of **3** (50 µM), in MeCN/H_2O (1:1), with increasing concentrations of 13 metal salts (0 - 7 equiv.). For all data points, λ_{exc} = 274 nm, λ_{em} = 461 nm.*

S2.8. Selectivity studies for **5** with $FeCl_3$

*Titration of **5** with $FeCl_3$ via fluorescence spectroscopy in the presence of 1 equiv. of other metal ions*

The initial concentration of **5** was 50 μM in MeCN/H_2O (1:1). Solutions of 12 different metal salt solutions (all 1 equiv.; Li^+, Na^+, K^+, Mg^{2+}, Ca^{2+}, Cr^{3+}, Mn^{2+}, Fe^{2+}, Cu^{2+}, Zn^{2+}, Al^{3+}, La^{3+}) were then added so that the overall concentration of **5** and the 12 metals in solution were 50 μM. Solutions of $FeCl_3$ in the same solvent system were then added to the solution of **5**, such that the concentration of **5** and the 12 other metals remained 50 μM, while the concentration of $FeCl_3$ is increased from 0 μM to 350 μM in 16 steps.

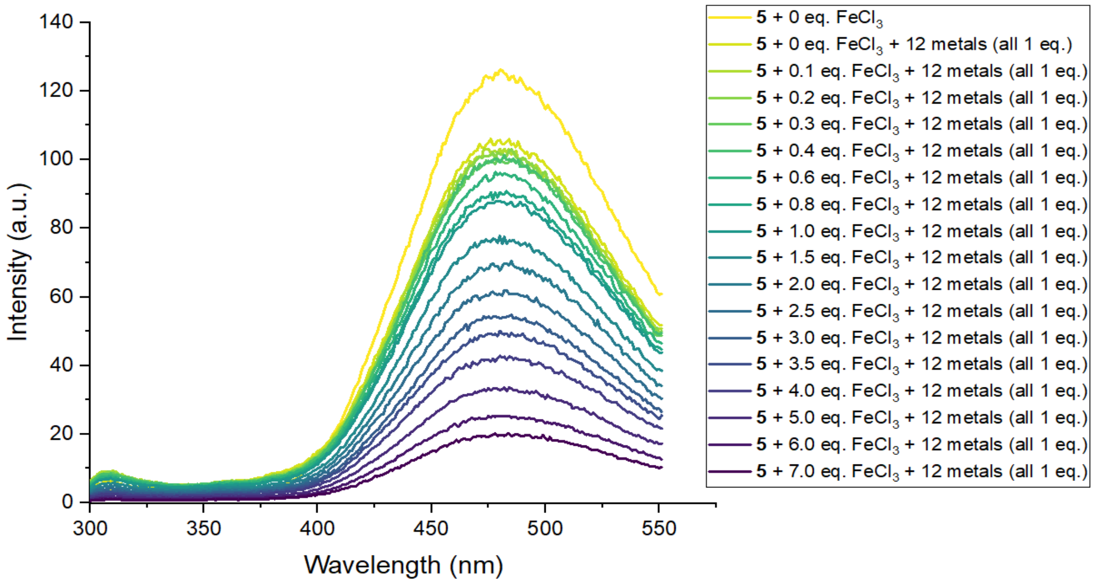

*Figure S29. Fluorescence spectra of **5** (50 μM), in MeCN/H_2O (1:1), with 1 equiv. of other 12 metal salts, and $FeCl_3$ (0 - 7 equiv.). λ_{exc} = 279 nm.*

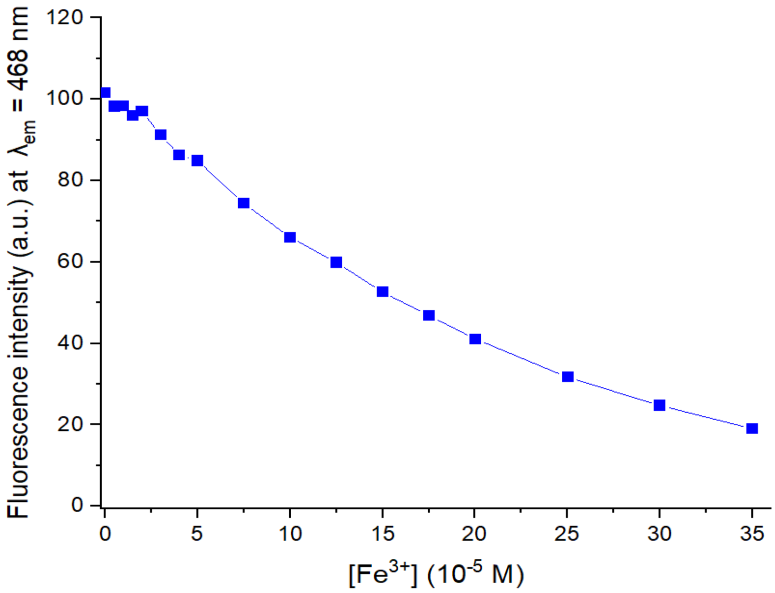

*Figure S30. Fluorescence intensities (a.u.) of **5** (50 μM), in MeCN/H_2O (1:1), with 1 equiv. of other 12 metal salts, and $FeCl_3$ (0 - 7 equiv.). For all data points, λ_{exc} = 279 nm, λ_{em} = 468 nm.*

*Titration of **5** with $FeCl_3$ via fluorescence spectroscopy with increasing concentrations of other metal ions*

The initial concentration of **5** was 50 μM in MeCN/H_2O (1:1). Solutions of 13 different metal salt solutions (Li^+, Na^+, K^+, Mg^{2+}, Ca^{2+}, Cr^{3+}, Mn^{2+}, Fe^{2+}, Cu^{2+}, Zn^{2+}, Al^{3+}, La^{3+}, **and** Fe^{3+}) in the same solvent system were then added to the solution of **5**, such that the concentration of **5** remained 50 μM, while the concentration of all other metals is increased from 0 μM to 350 μM in 15 steps.

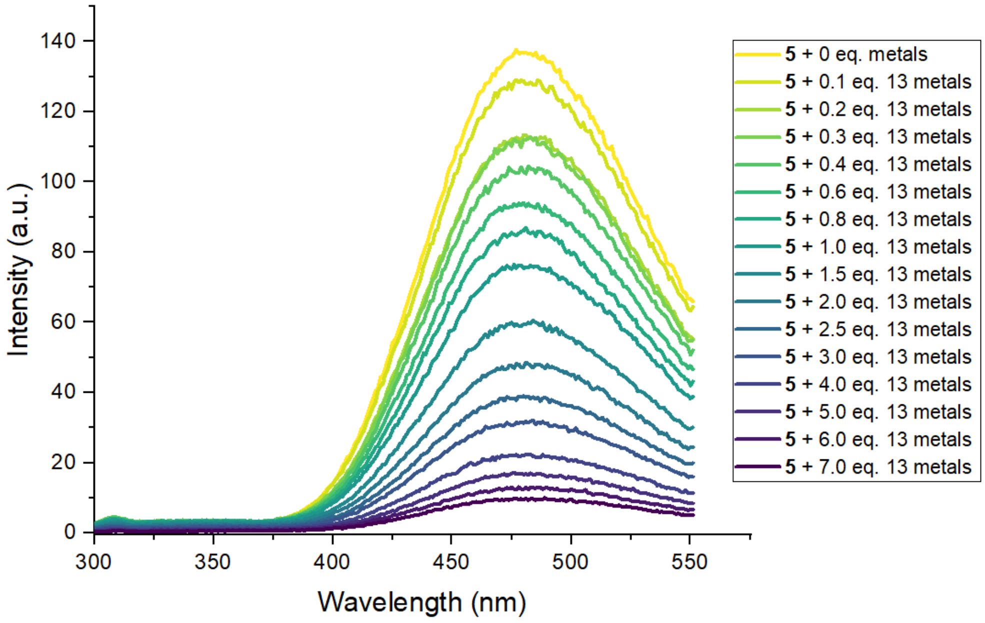

*Figure S31. Fluorescence spectra of **5** (50 μM), in MeCN/H_2O (1:1), with increasing concentrations of 13 metal salts (0 - 7 equiv.). λ_{exc} = 279 nm.*

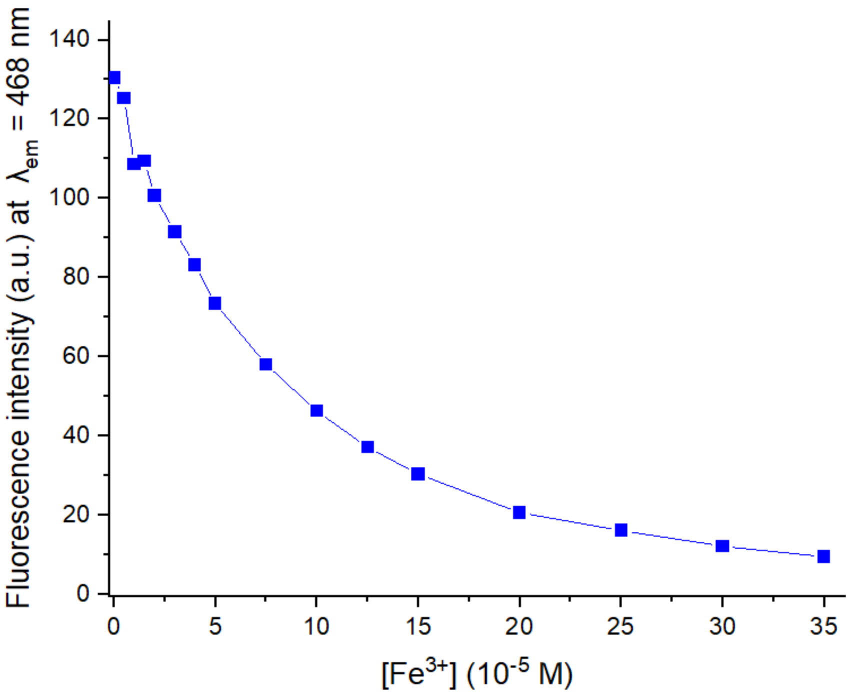

*Figure S32. Fluorescence intensities (a.u.) of **5** (50 μM), in MeCN/H_2O (1:1), with increasing concentrations of 13 metal salts (0 - 7 equiv.). For all data points, λ_{exc} = 279 nm, λ_{em} = 468 nm.*

S2.9. Determination of detection limits for **3** and **5** with $FeCl_3$

*Limit of detection of $FeCl_3$ with **5** in the absence of other metal ions:*

The detection limit was calculated from the sample standard deviation of the blank (σ) and the slope (S) of the calibration curve for **5**/$FeCl_3$ (see Figure S16). Five independent readings were performed for the determination σ. of The slope of the curve was taken from the linear section of the curve, for values of $[Fe^{3+}] \leq 200$ μM.

*DL = 3*σ/ S*

Reading 1	Reading 2	Reading 3	Reading 4	Reading 5	Mean	Sample standard deviation
141.20	149.95	140.88	148.81	144.10	144.99	4.22

S = -3.7481 ± 0.14449 x10^{-5} M

Therefore, detection limit = 0.338 ± 0.013 μM

*Limit of detection of $FeCl_3$ with **5** in the presence of other metal ions:*

The detection limit was calculated from the sample standard deviation of the blank (σ) and the slope (S) of the calibration curve for **5**/$FeCl_3$ in the presence of 12 other metal ions (all 1 equiv.; see Figure S28). Three independent readings were performed for the determination σ. The slope of the curve was taken from the linear section of the curve, for values of $[Fe^{3+}] \leq 200$ μM.

*DL = 3*σ/ S*

Reading 1	Reading 2	Reading 3	Mean	Sample standard deviation
101.59	110.94	108.99	107.17	4.93

S = -3.11618 ± 0.07853 x10^{-5} M

Therefore, detection limit = 0.475 ± 0.012 μM

*Limit of detection of $FeCl_3$ with **3** in the absence of other metal ions:*

The detection limit was calculated from the sample standard deviation of the blank (σ) and the slope (S) of the calibration curve for **3**/$FeCl_3$ (see Figure S13). Four independent readings were performed for the determination σ. of The slope of the curve was taken from the linear section of the curve, for values of [Fe^{3+}] $\leq$ 200 μM.

*DL = 3*σ/ S*

Reading 1	Reading 2	Reading 3	Reading 4	Mean	Sample standard deviation
371.07	368.51	379.38	367.73	371.67	5.34

S = -11.99543 ± 0.22442 x10^{-5} M

Therefore, detection limit = 0.134 ± 0.003 μM

*Limit of detection of $FeCl_3$ with **3** in the presence of other metal ions:*

The detection limit was calculated from the sample standard deviation of the blank (σ) and the slope (S) of the calibration curve for **3**/$FeCl_3$ in the presence of 12 other metal ions (all 1 equiv.; see Figure S24). Three independent readings were performed for the determination σ. The slope of the curve was taken from the linear section of the curve, for values of [Fe^{3+}] $\leq$ 50 μM.

*DL = 3*σ/ S*

Reading 1	Reading 2	Reading 3	Mean	Sample standard deviation
591.05	621.27	575.73	596.02	23.17

S = - 44.77498 ± 5.30371 x10^{-5} M

Therefore, detection limit = 0.155 ± 0.021 μM

S3. Characterisation Data

S3.1. NMR Spectra

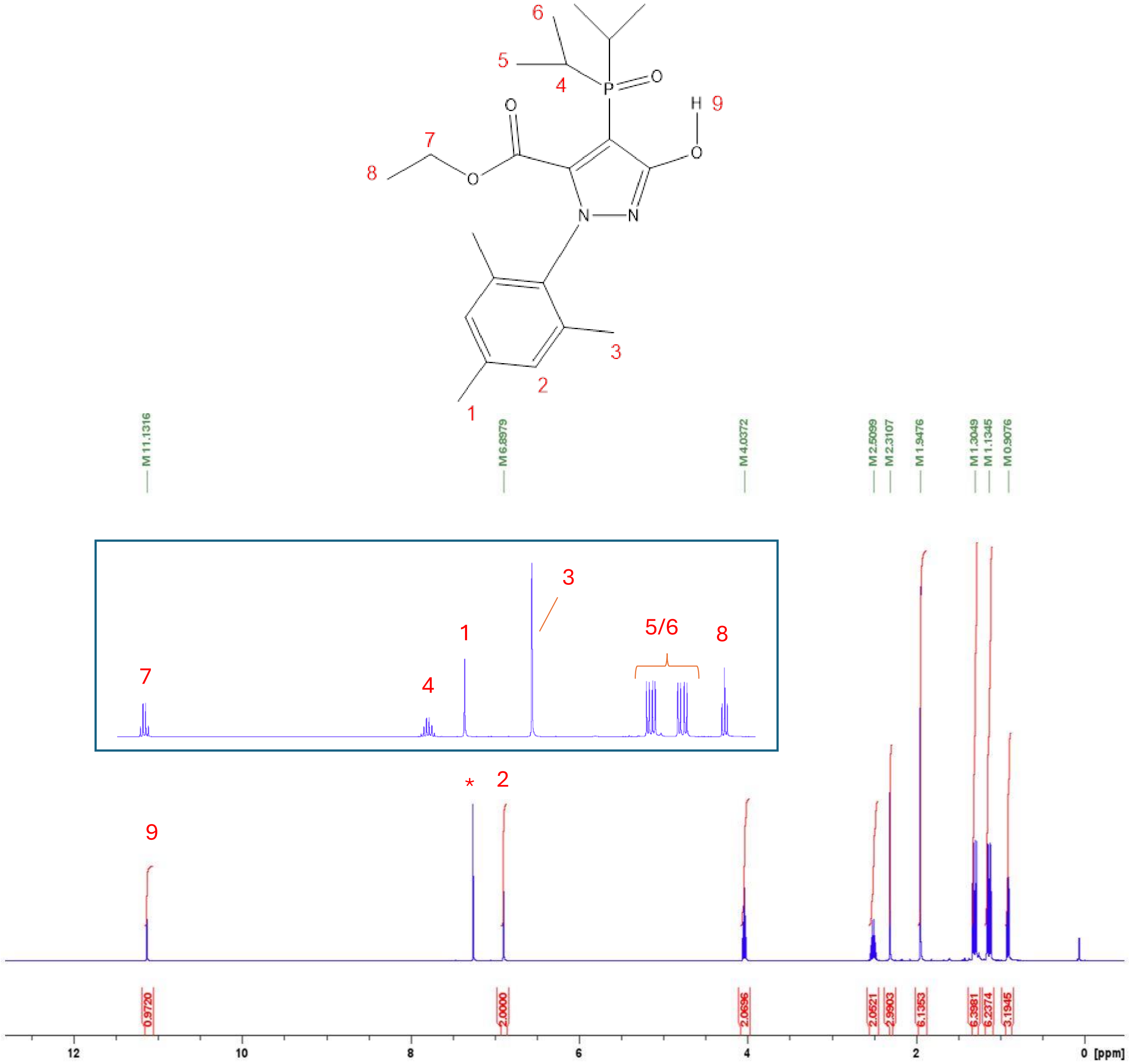

*Figure S33. ^{1}H NMR spectrum of **3** in $CDCl_3$. The spectrum is calibrated to the residual $CHCl_3$ peak at 7.26 ppm, marked with *.*

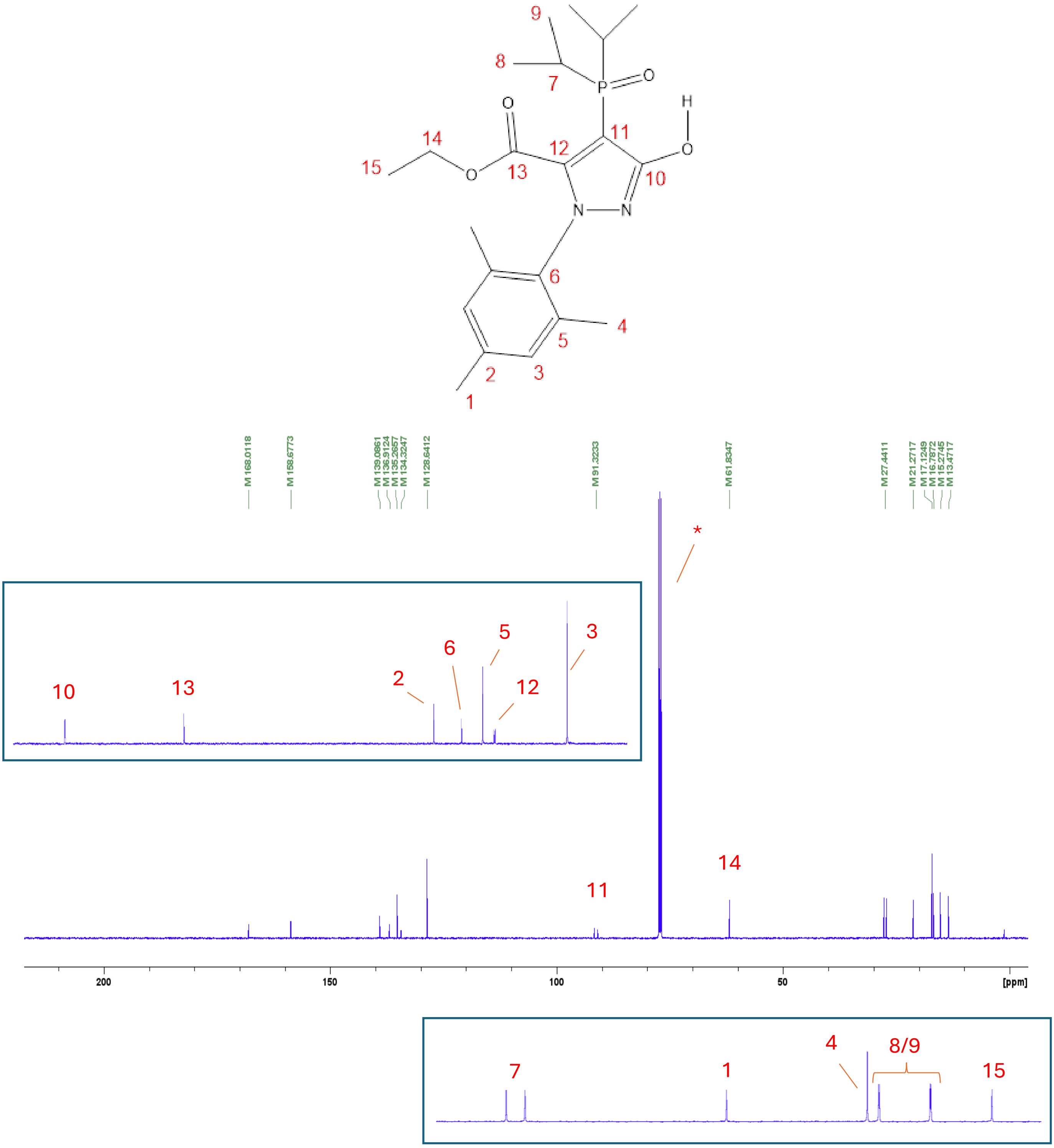

*Figure S34. $^{13}C\{^{1}H\}$ NMR spectrum of **3** in $CDCl_3$. * = $CDCl_3$ peak, 77.16 ppm, to which the spectrum is calibrated.*

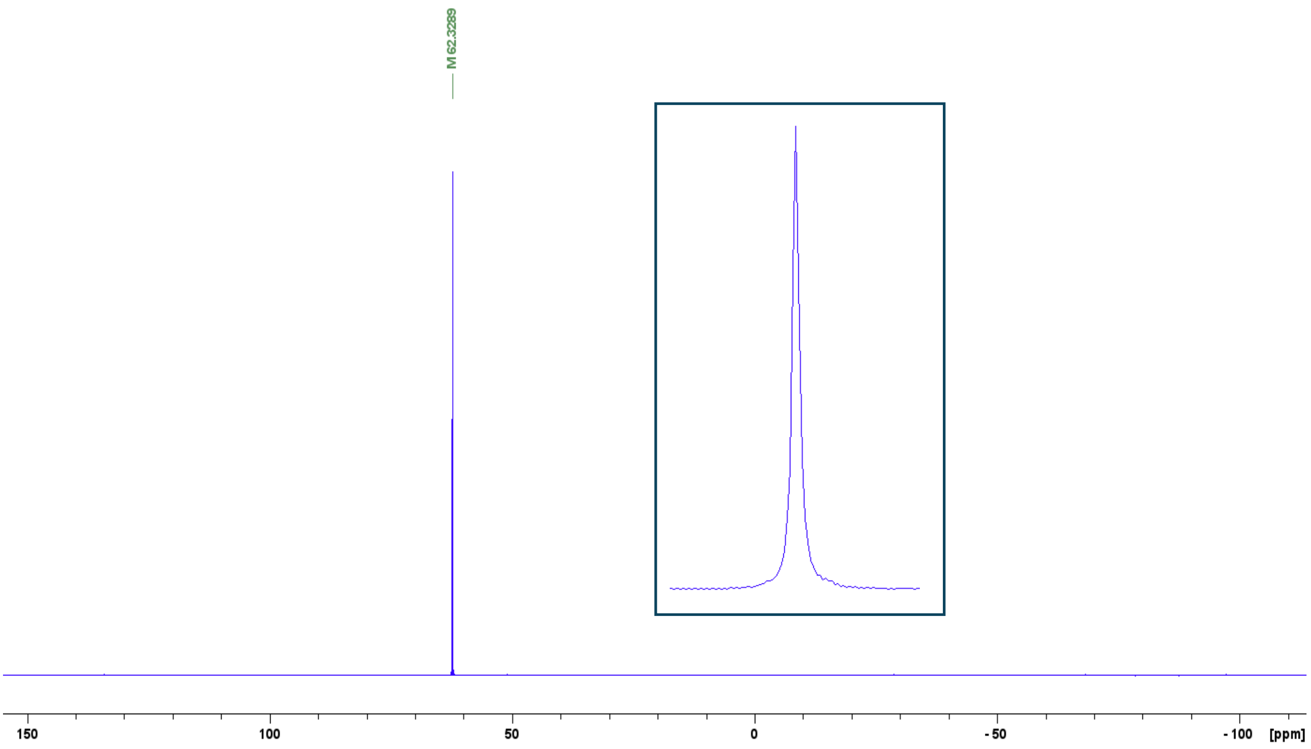

Figure S35. $^{31}P\{^{1}H\}$ *NMR spectrum of* ***3*** *in* $CDCl_3$.

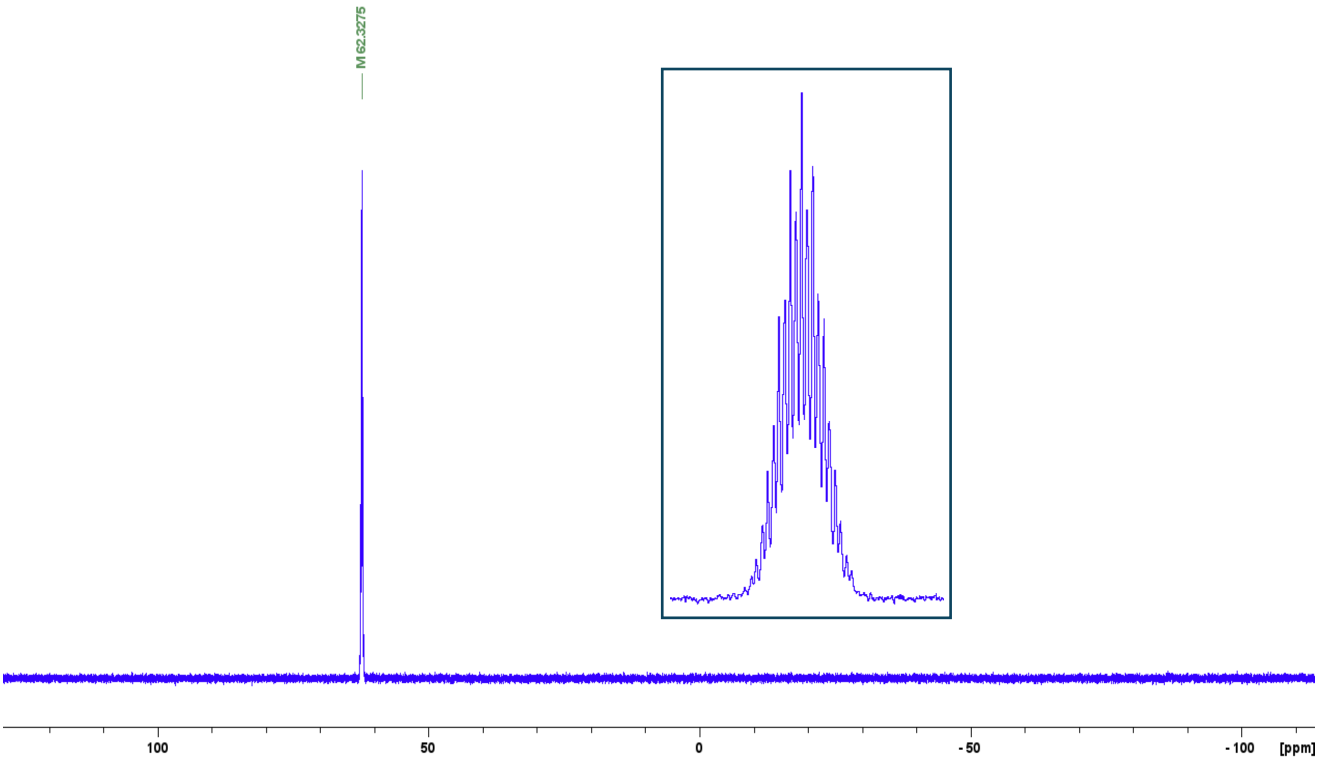

Figure S36. ^{31}P *NMR spectrum of* ***3*** *in* $CDCl_3$*. Figure inside the blue box is zoomed in to show the splittings of the peak.*

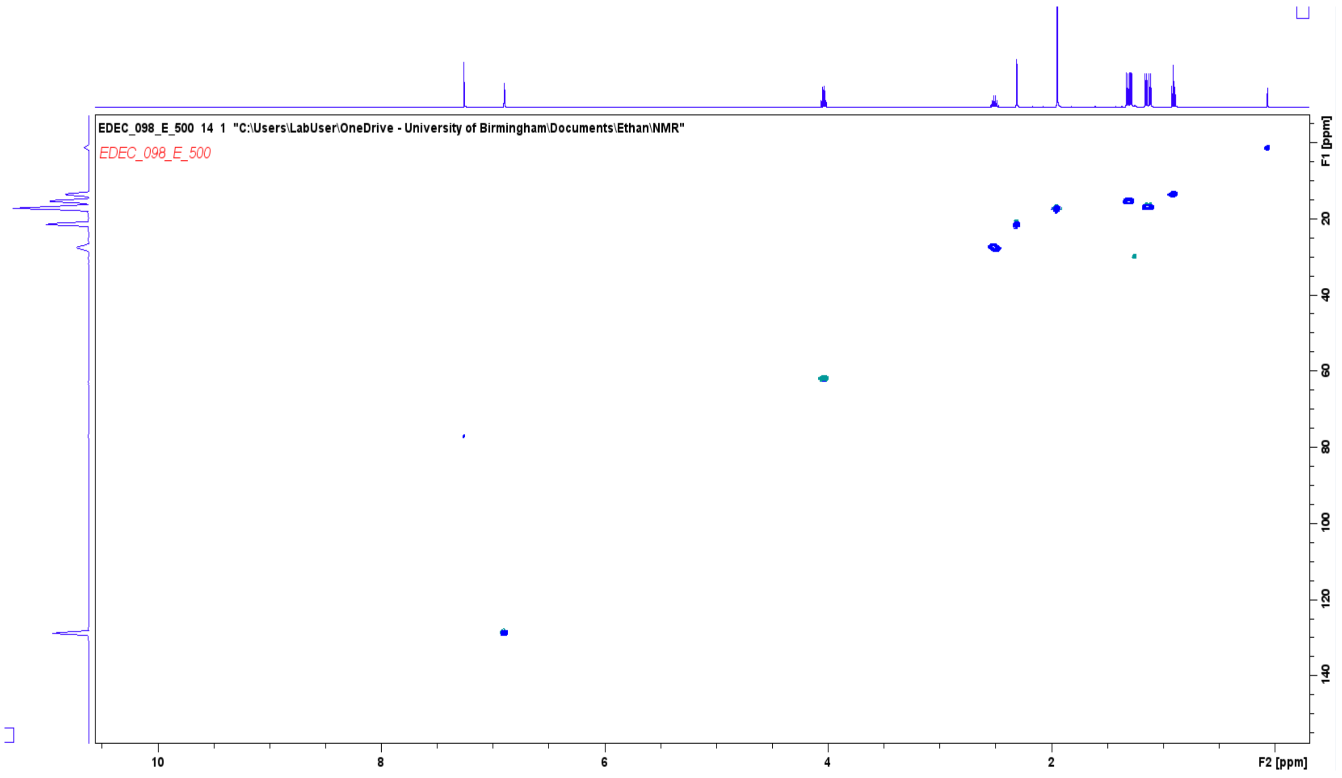

*Figure S37. 2D ^{1}H-^{13}C HSQC NMR spectrum of **3** in $CDCl_3$.*

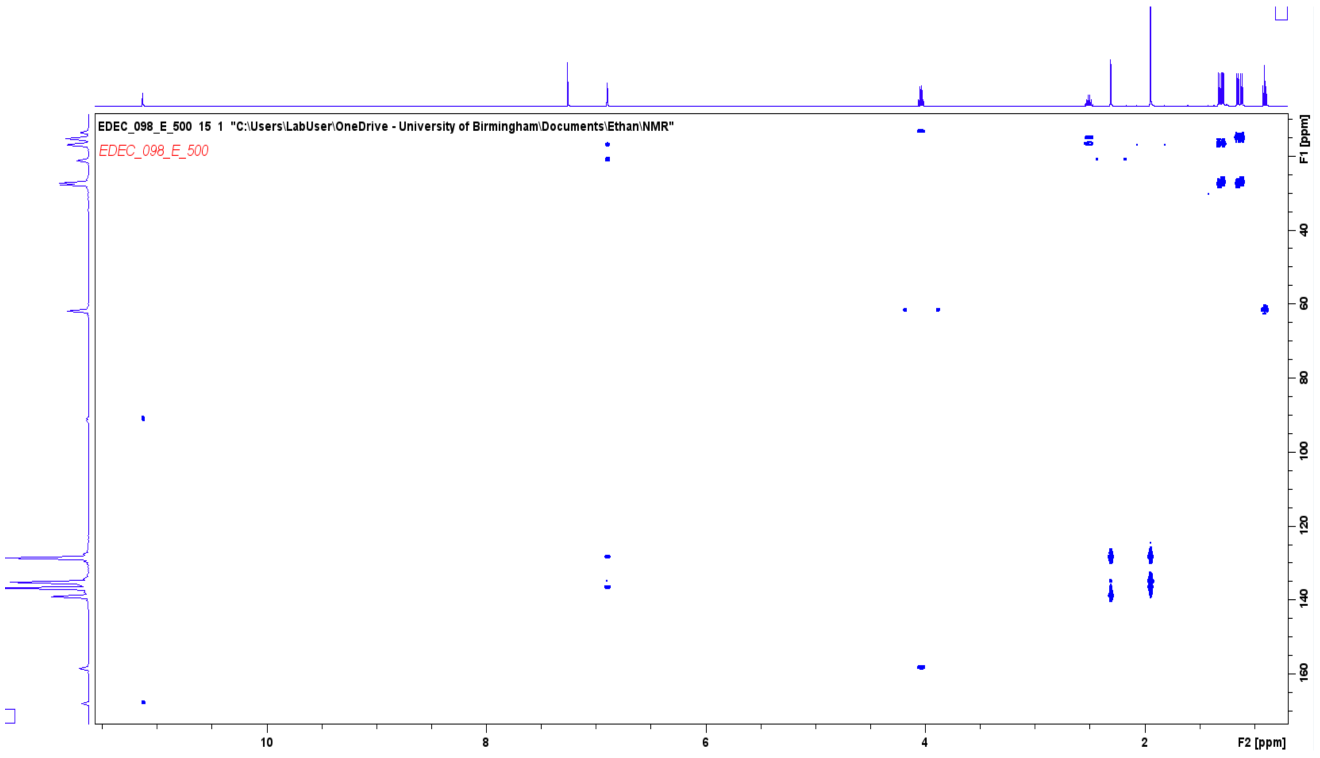

*Figure S38. 2D ^{1}H-^{13}C HMBC NMR spectrum of **3** in $CDCl_3$.*

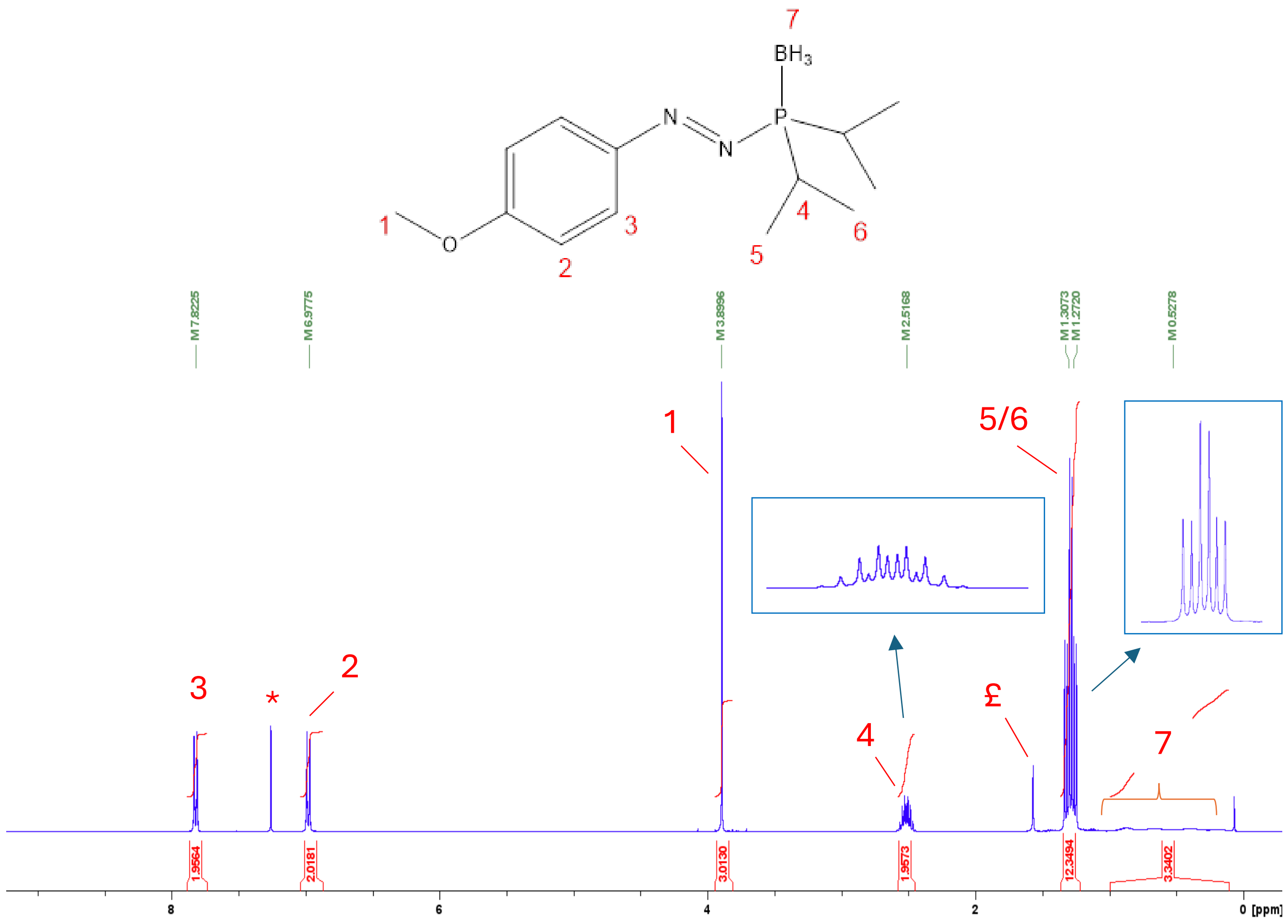

Figure S39. [1]*H NMR spectrum of* ***4*** *in* $CDCl_3$*. * = residual* $CHCl_3$*, 7.26 ppm, to which the spectrum is calibrated; £ = residual* H_2O *from the non-dried NMR solvent, 1.56 ppm. Figures inside the blue boxes are zoomed in to show the splittings of the multiplets.*

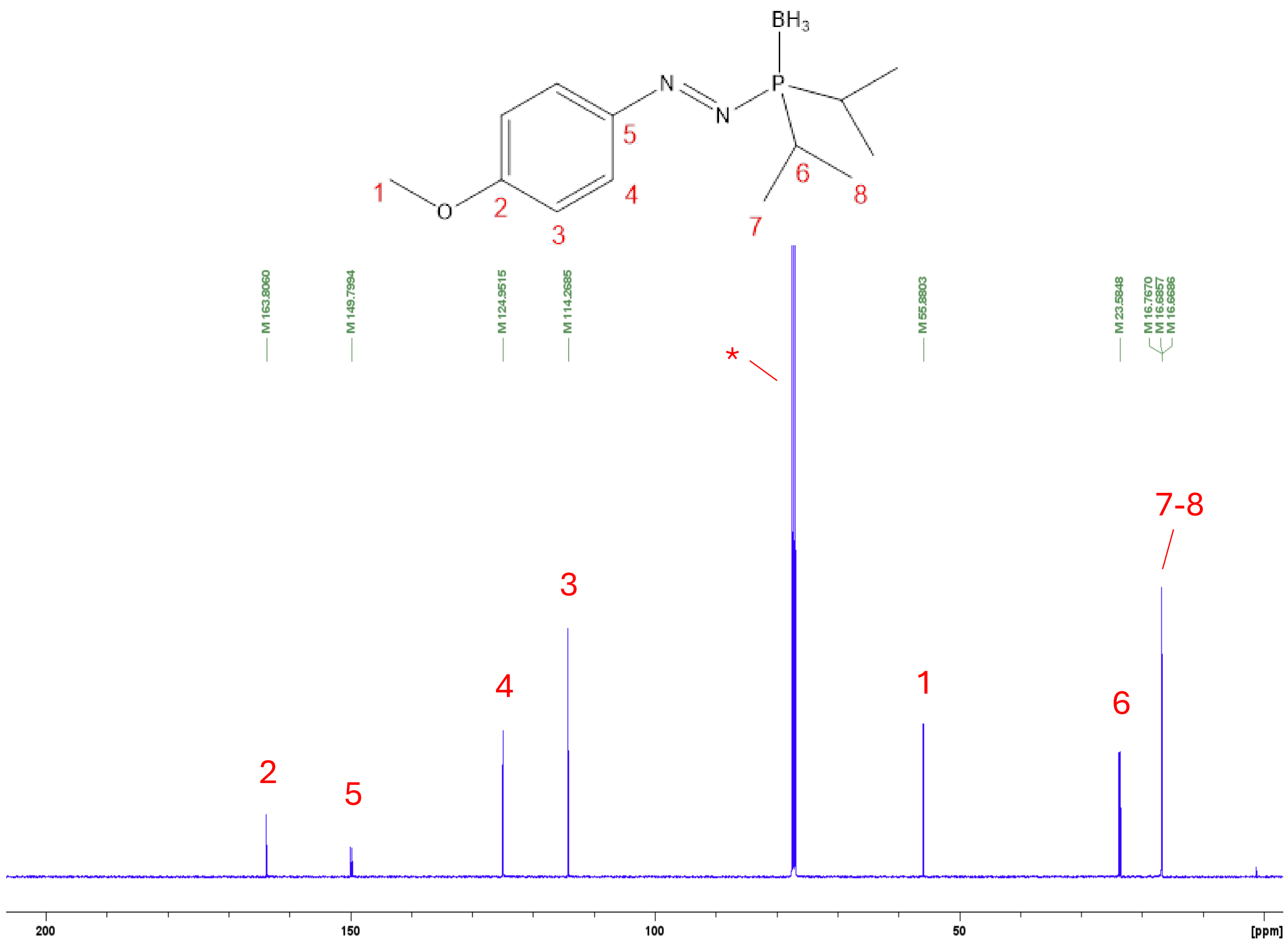

*Figure S40. $^{13}C\{^1H\}$ NMR spectrum of **4** in $CDCl_3$. * = $CDCl_3$ peak, 77.16 ppm, to which the spectrum is calibrated.*

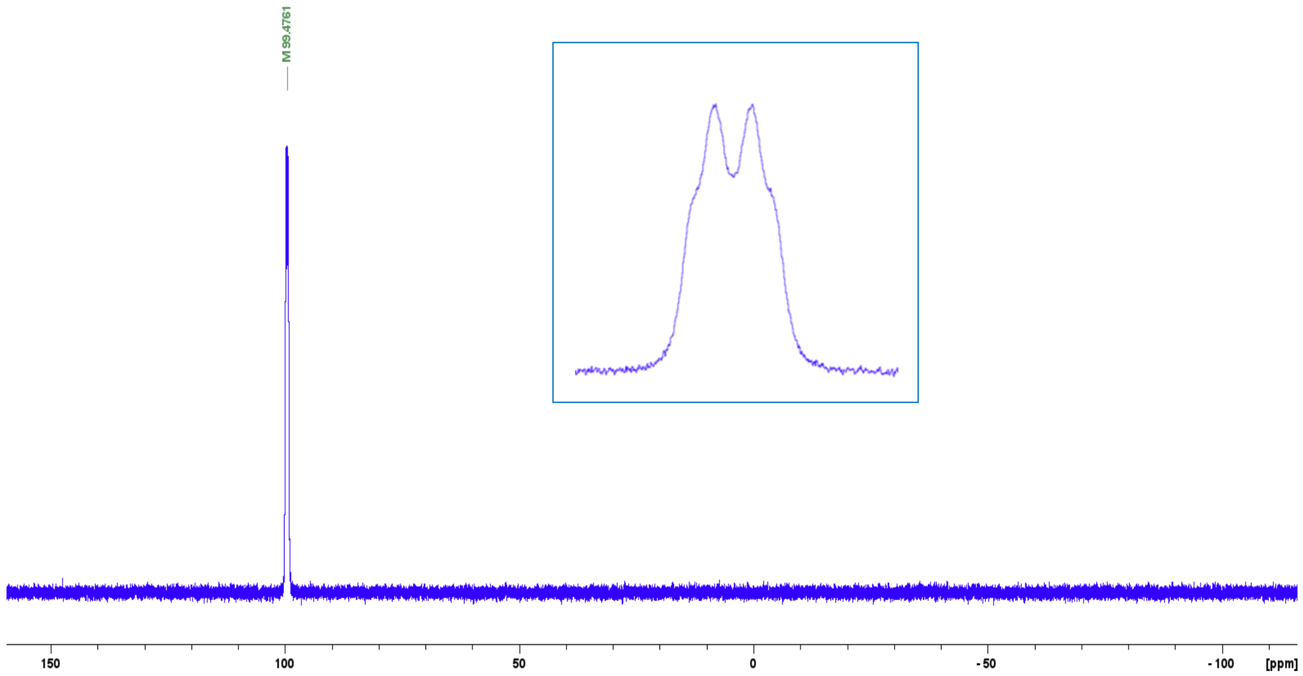

*Figure S41. $^{31}P\{^{1}H\}$ NMR spectrum of **4** in $CDCl_3$. Figure inside the blue box is zoomed in to show the splittings of the peak.*

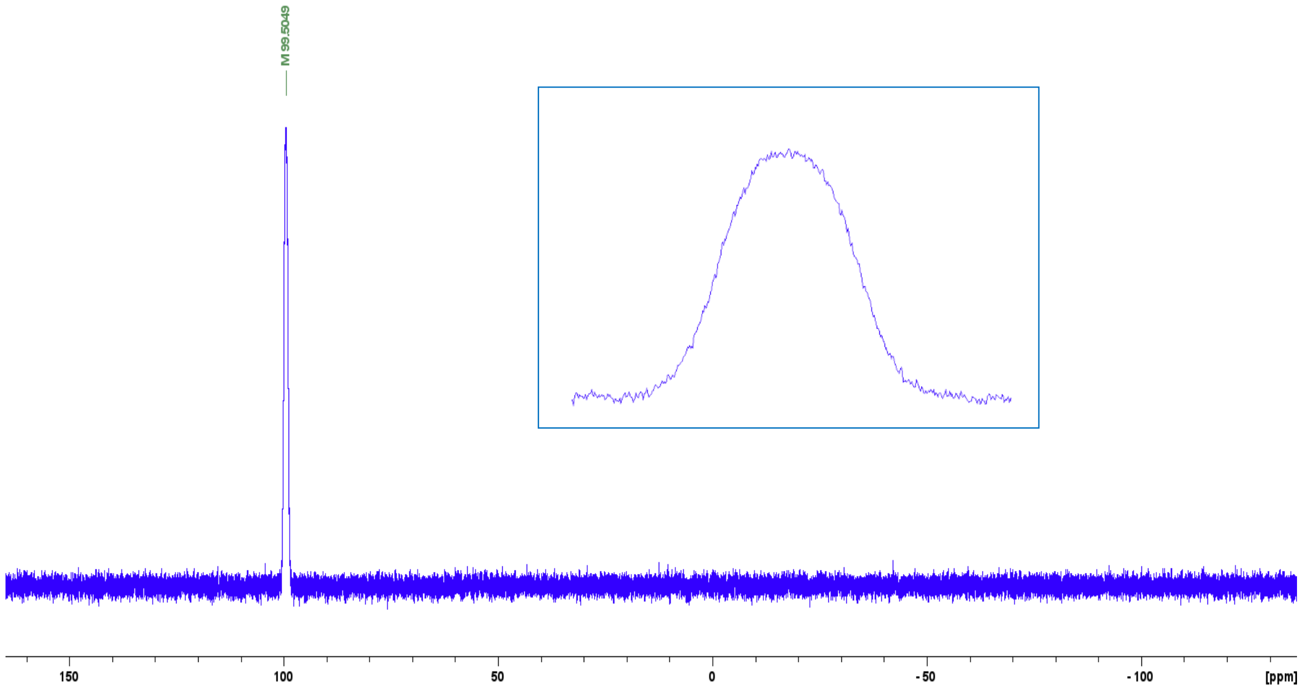

*Figure S42. ^{31}P NMR spectrum of **4** in $CDCl_3$. Figure inside the blue box is zoomed in to show the splittings of the peak.*

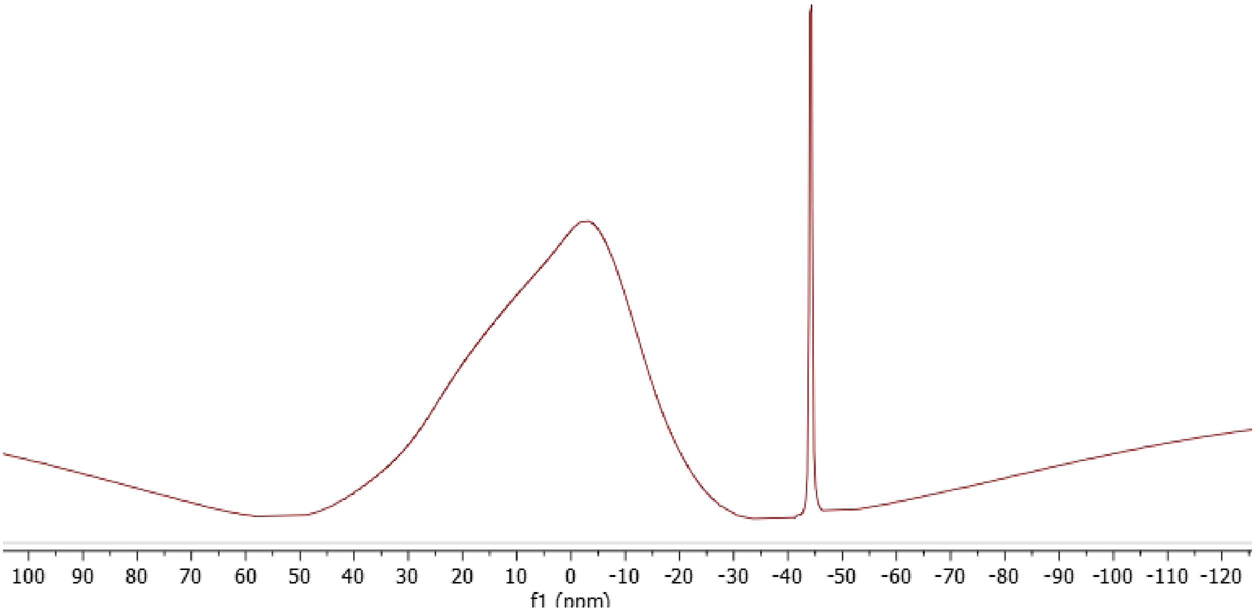

Figure S43. $^{11}B\{^{1}H\}$ *NMR spectrum of* ***4*** *in* $CDCl_3$*.*

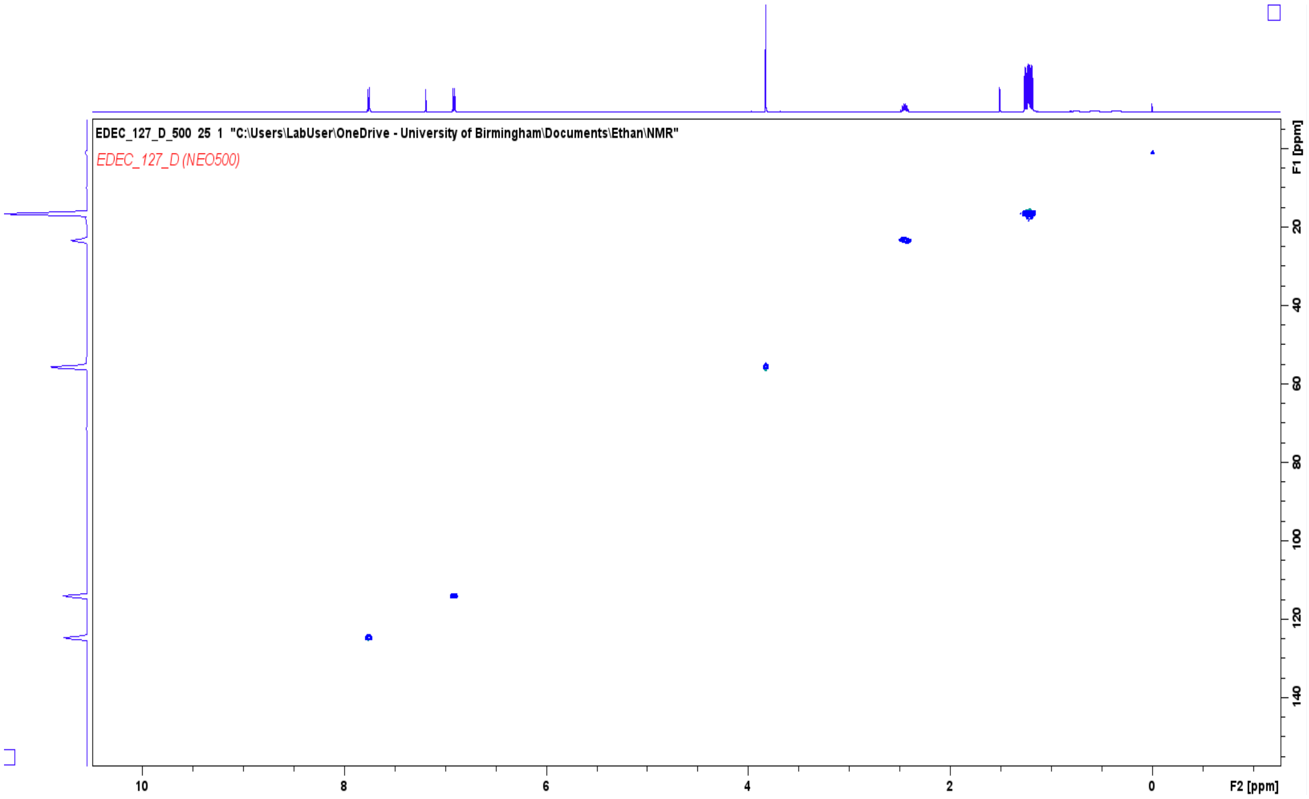

Figure S44. 2D ^{1}H*-*^{13}C *HSQC NMR spectrum of* ***4*** *in* $CDCl_3$*.*

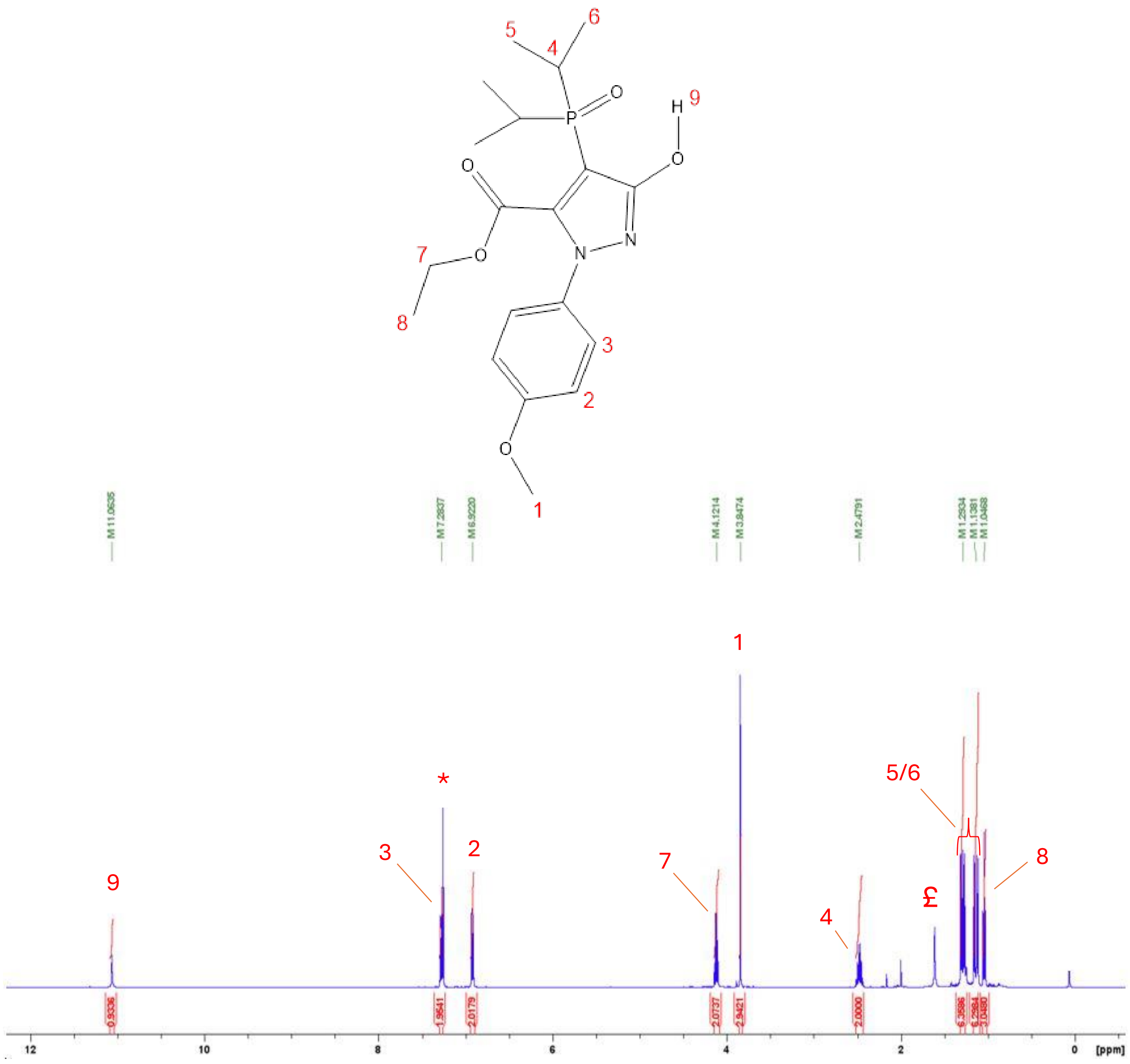

*Figure S45. ^{1}H NMR spectrum of **5** in $CDCl_3$. * = residual $CHCl_3$, 7.26 ppm, to which the spectrum is calibrated; £ = residual H_2O from the non-dried NMR solvent, 1.56 ppm.*

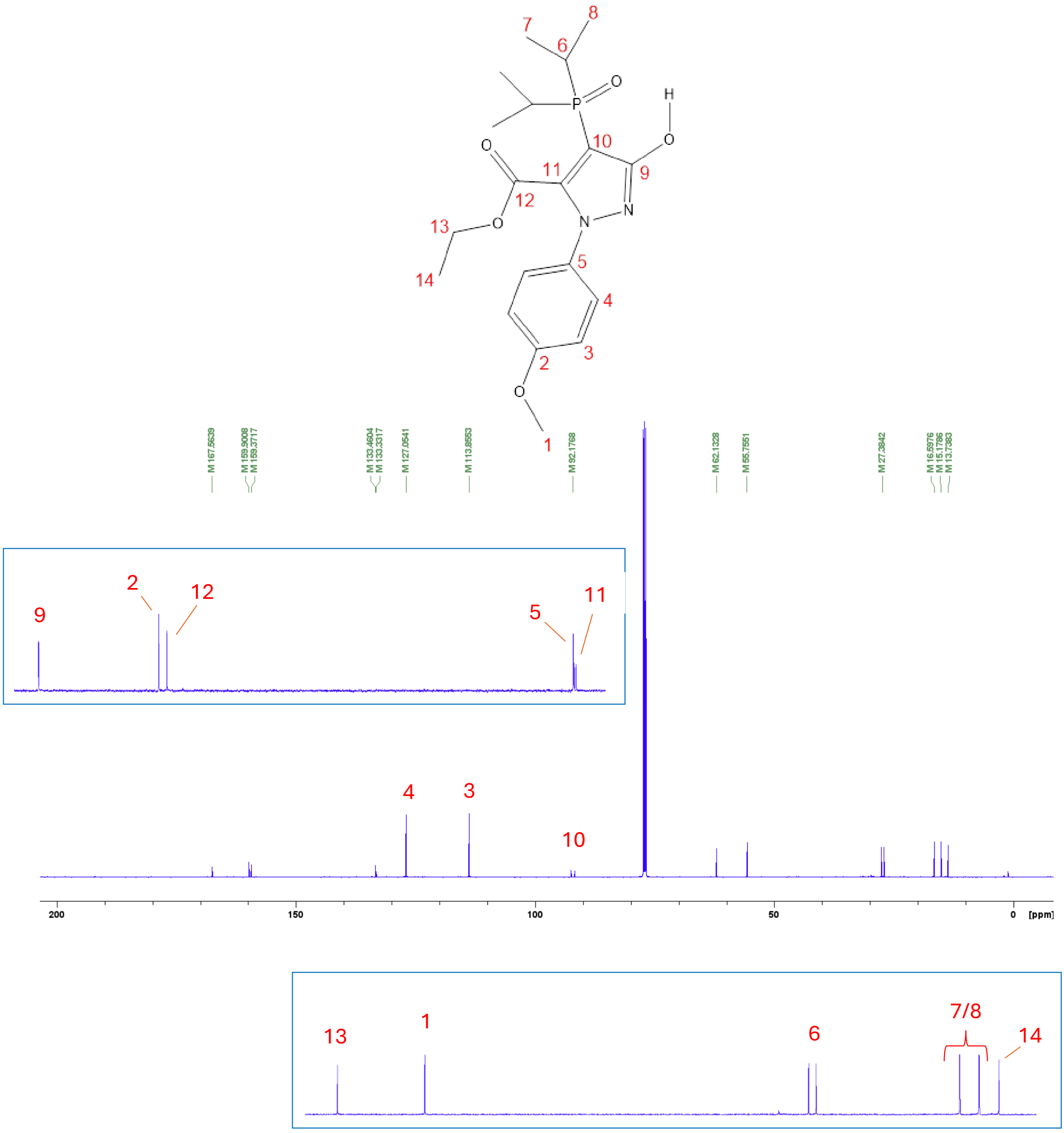

*Figure S46. $^{13}C\{^1H\}$ NMR spectrum of **5** in $CDCl_3$. * = $CDCl_3$ peak, 77.16 ppm, to which the spectrum is calibrated.*

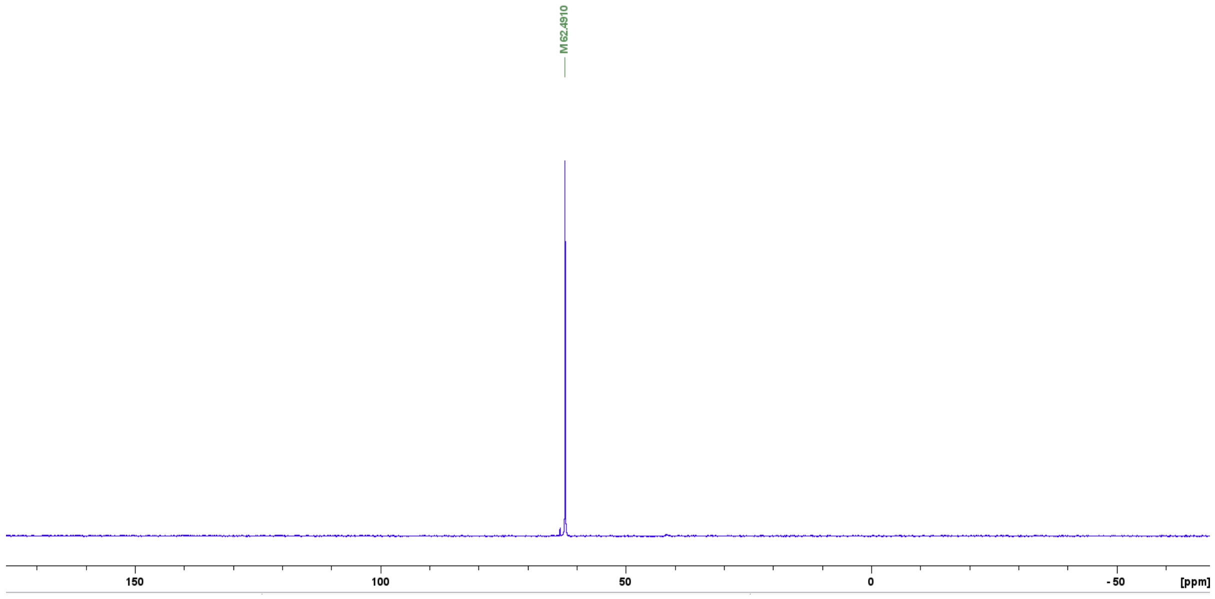

*Figure S47. $^{31}P\{^{1}H\}$ NMR spectrum of **5** in $CDCl_3$.*

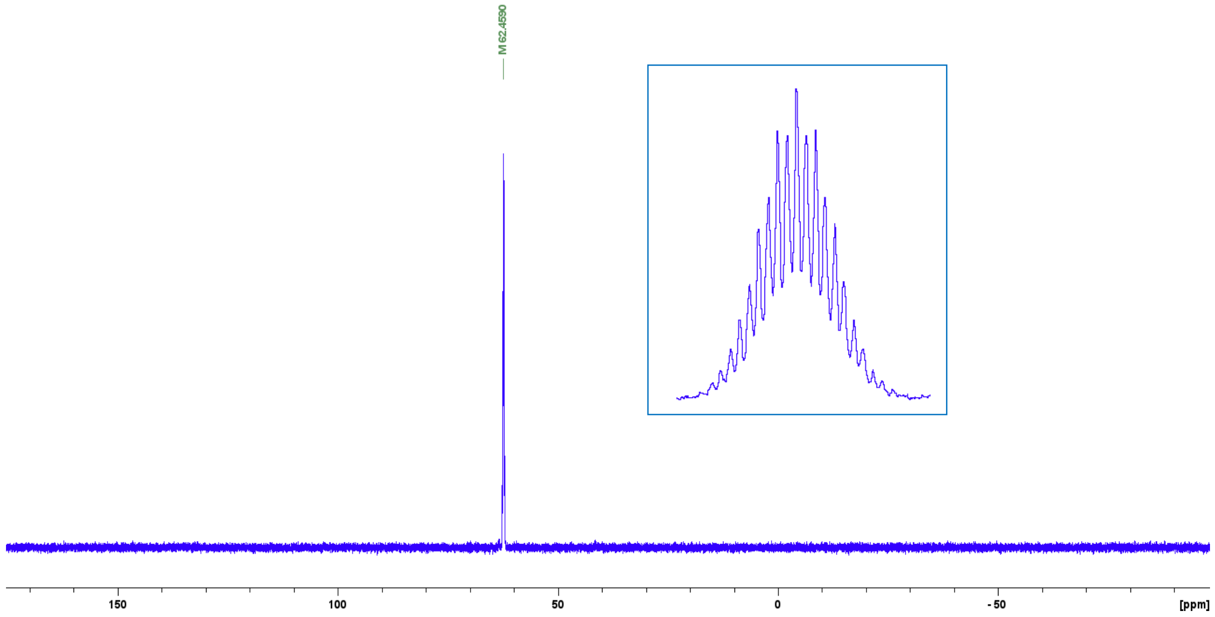

*Figure S48. ^{31}P NMR spectrum of **5** in $CDCl_3$. Figure inside the blue box is zoomed in to show the splittings of the peak.*

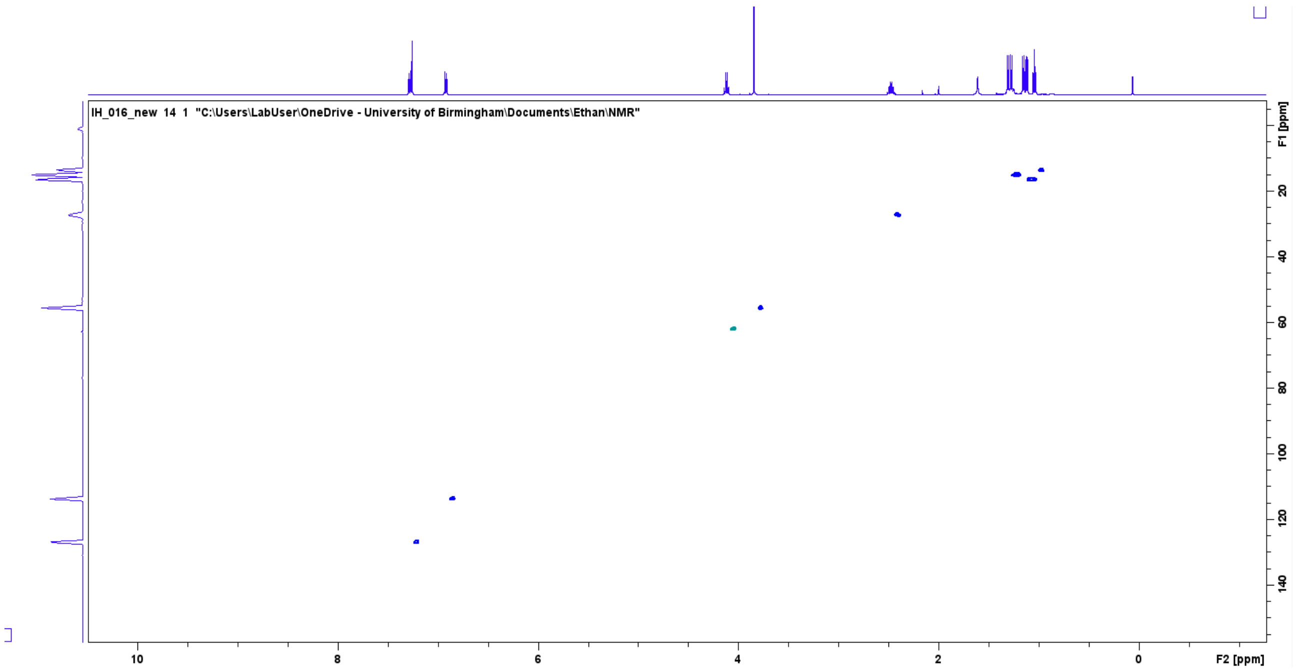

*Figure S49. 2D ^{1}H-^{13}C HSQC NMR spectrum of **5** in $CDCl_3$.*

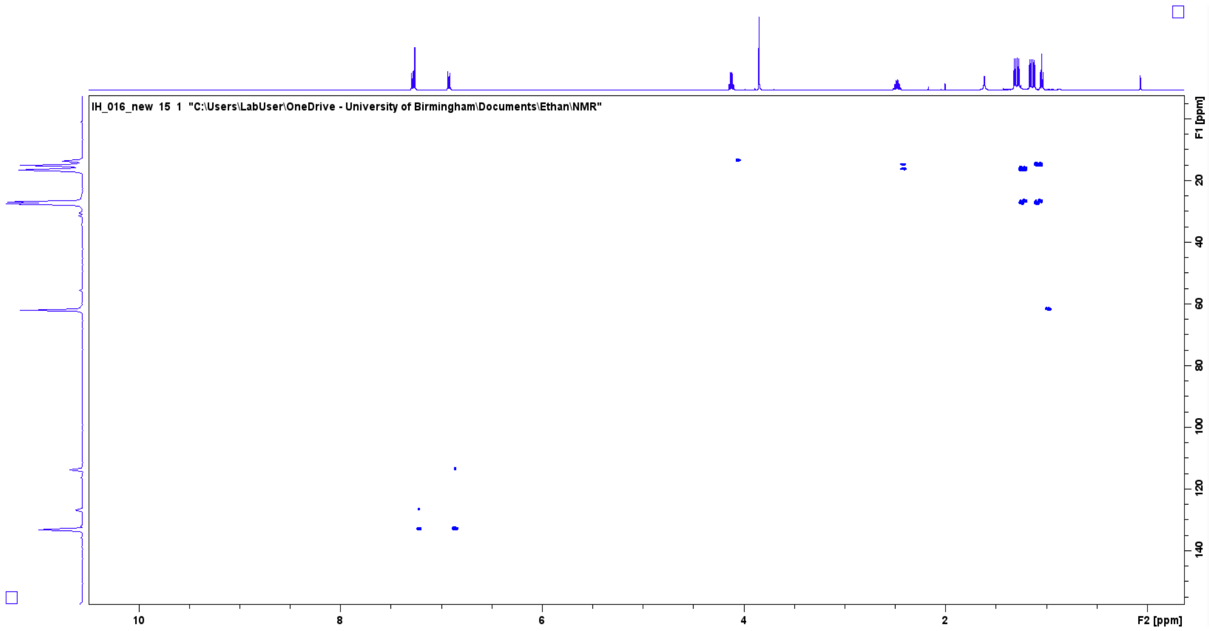

*Figure S50. 2D ^{1}H-^{13}C HMBC NMR spectrum of **5** in $CDCl_3$.*

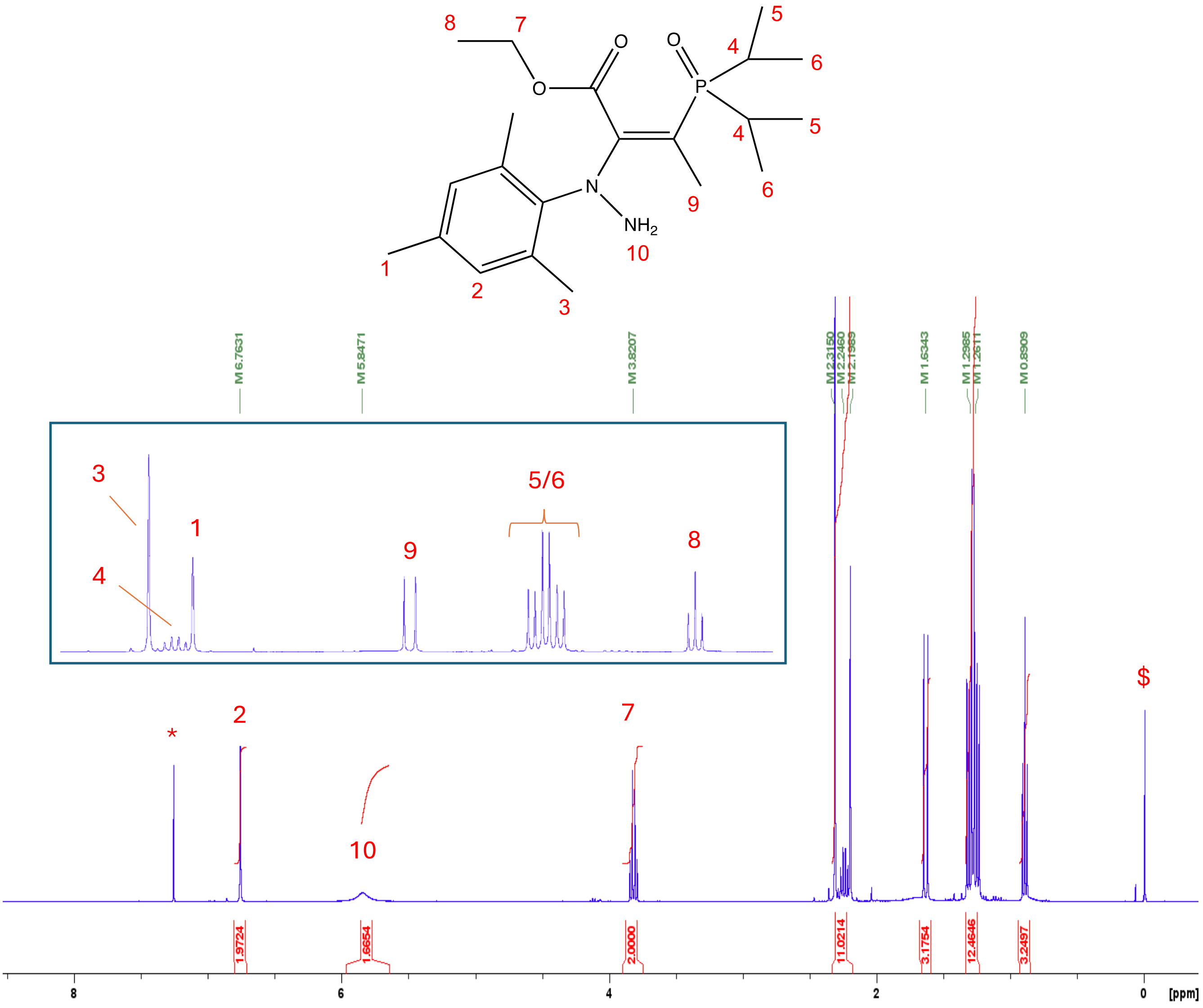

*Figure S51. ¹H NMR spectrum of **7** in $CDCl_3$. $ = TMS, 0.00 ppm. * = residual $CHCl_3$ peak, 7.26 ppm, to which the spectrum is calibrated. Protons 5 and 6 are assigned as two overlapping, but distinct double doublets. This was confirmed by running the same spectrum at a different frequency (500 vs 400 MHz) gave deconvolution of the peaks into their constituent double doublets (not shown here).*

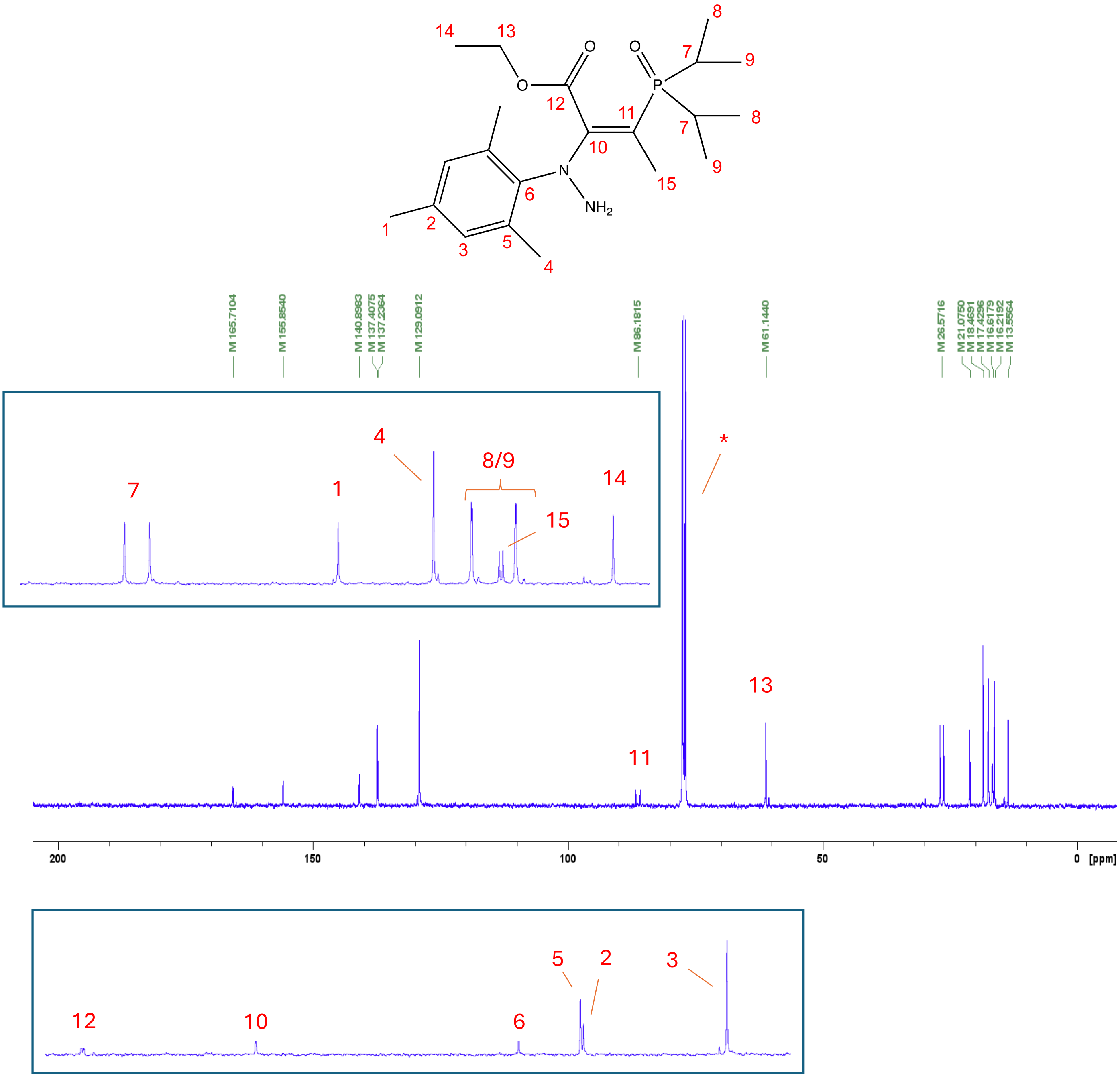

*Figure S52. ^{13}C{^{1}H} NMR spectrum of **7** in $CDCl_3$. * = $CDCl_3$ peak, 77.16 ppm, to which the spectrum is calibrated. Figures inside blue boxes show insets of the same spectrum.*

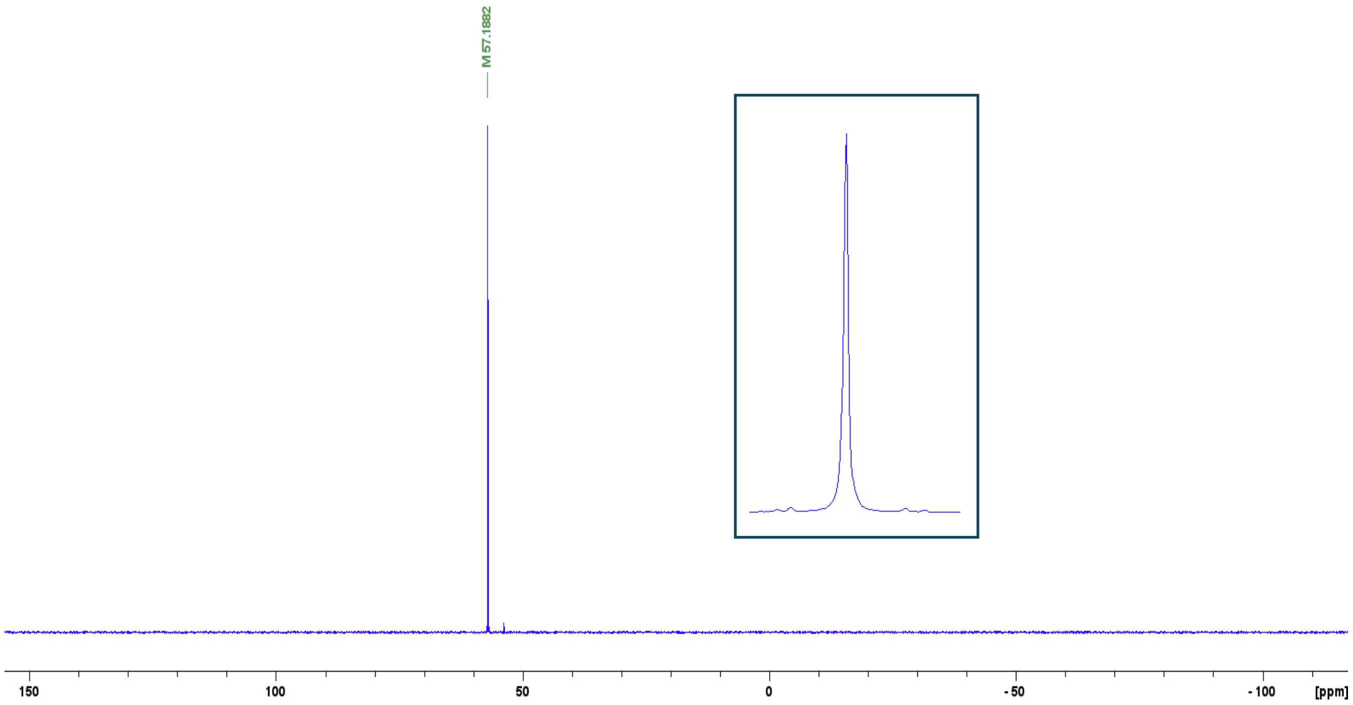

*Figure S53. $^{31}P\{^{1}H\}$ NMR spectrum of **7** in $CDCl_3$. Figure inside the blue box is zoomed in to show the splittings of the peak.*

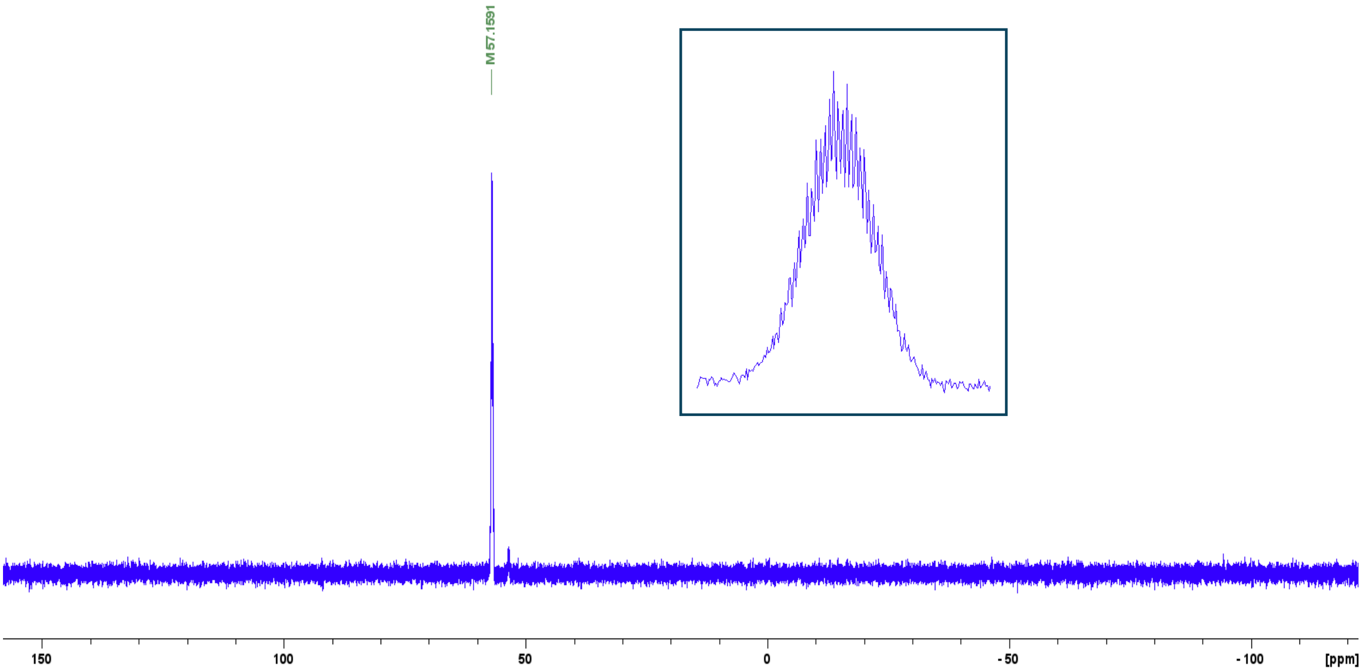

*Figure S54. ^{31}P NMR spectrum of **7** in $CDCl_3$. Figure inside the blue box is zoomed in to show the splittings of the peak.*

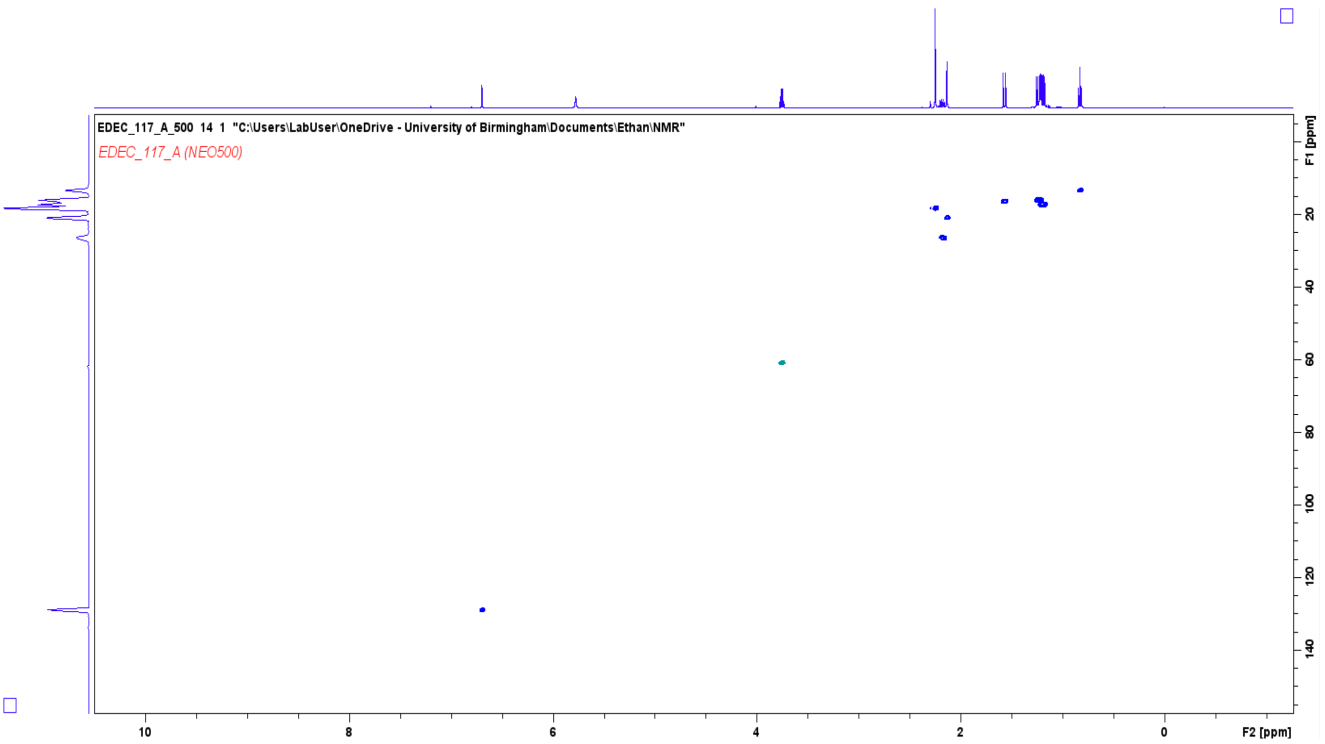

*Figure S55. 2D ^{1}H-^{13}C HSQC NMR spectrum of **7** in $CDCl_3$.*

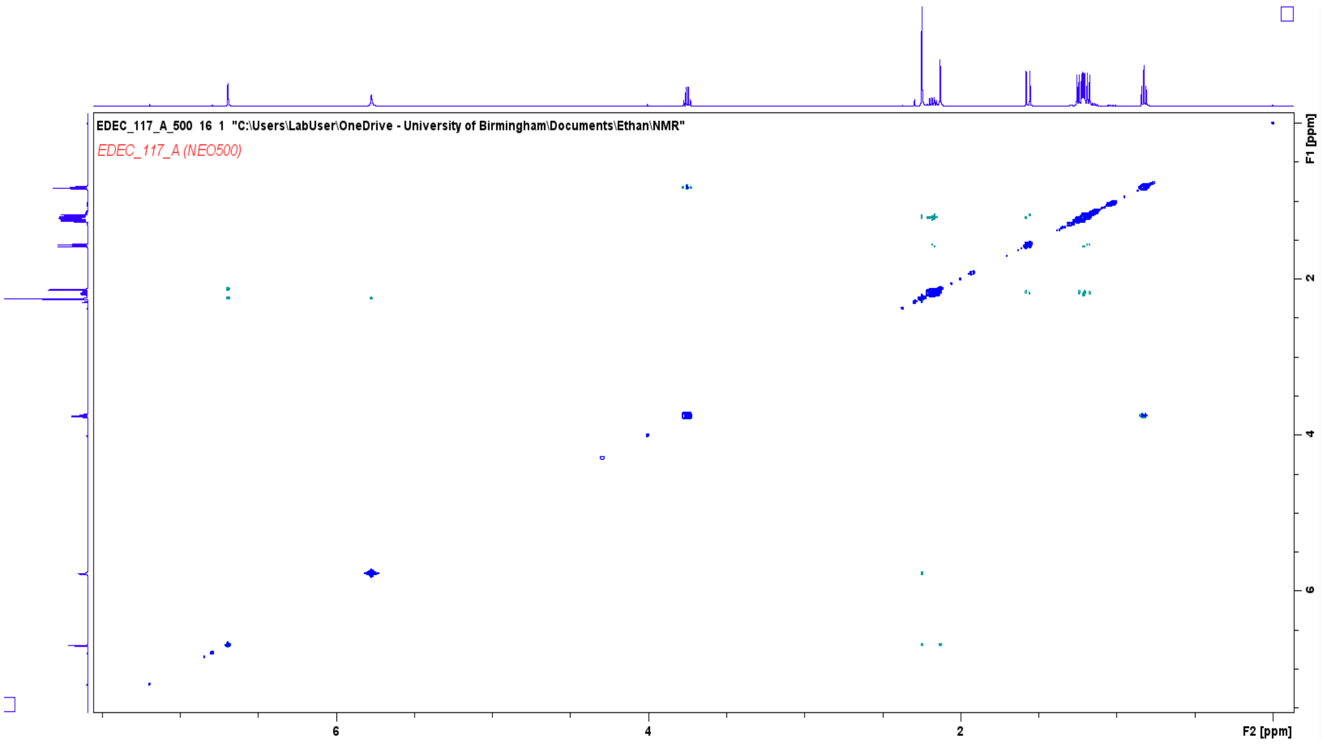

*Figure S56. 2D ^{1}H-^{13}C HMBC NMR spectrum of **7** in $CDCl_3$.*

S3.2. UV/Vis Spectra for **4** and **7**

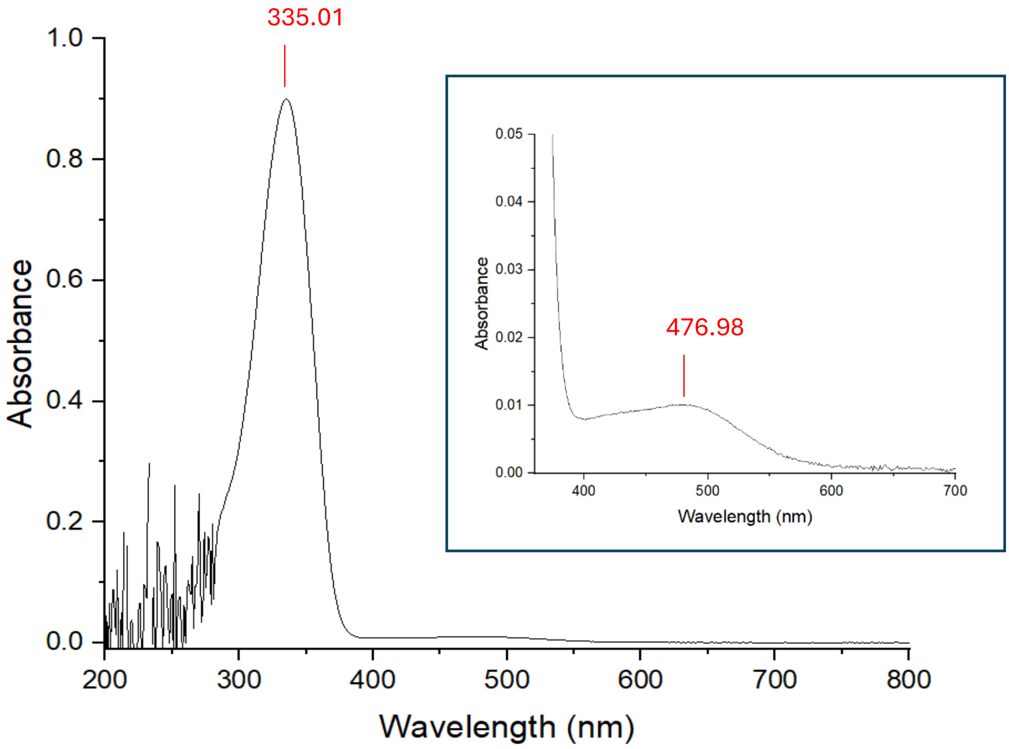

*Figure S57. UV/Vis Spectrum of **4** (5 x 10^{-5} M in toluene). Figure inside the blue box is zoomed in to show the absorbance peak at 477 nm.*

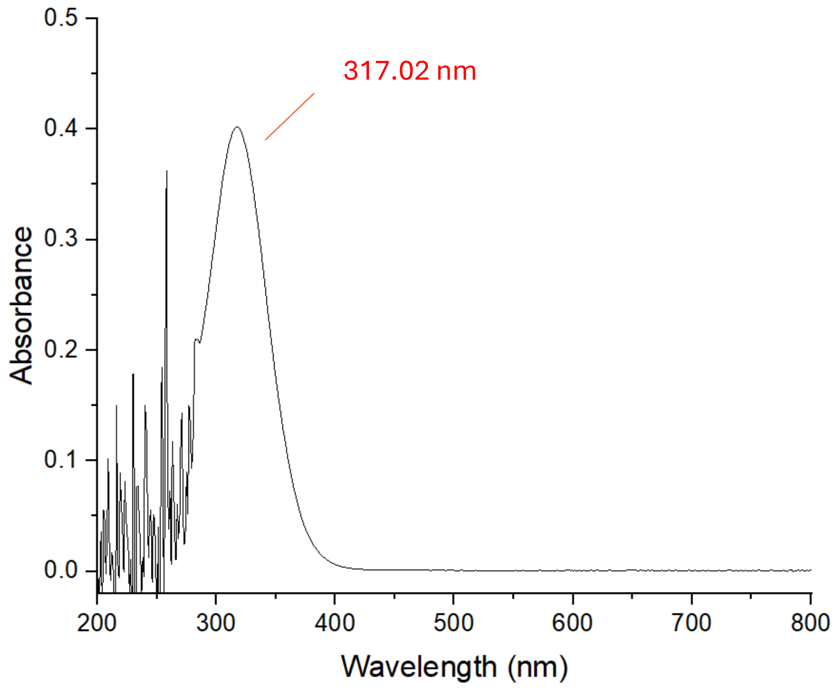

*Figure S58. UV/Vis Spectrum of **7** (5 x 10^{-5} M in toluene).*

S3.3. Mass Spectra

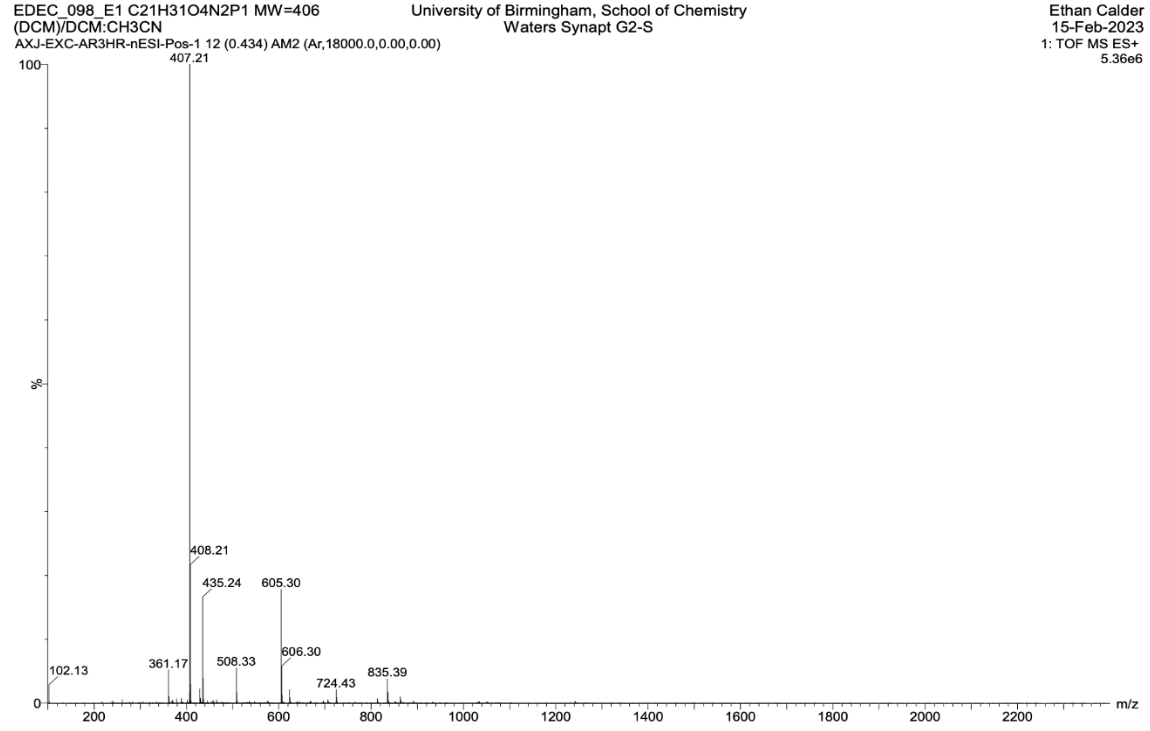

*Figure S59. Mass spectrum of **3** in dichloromethane.*

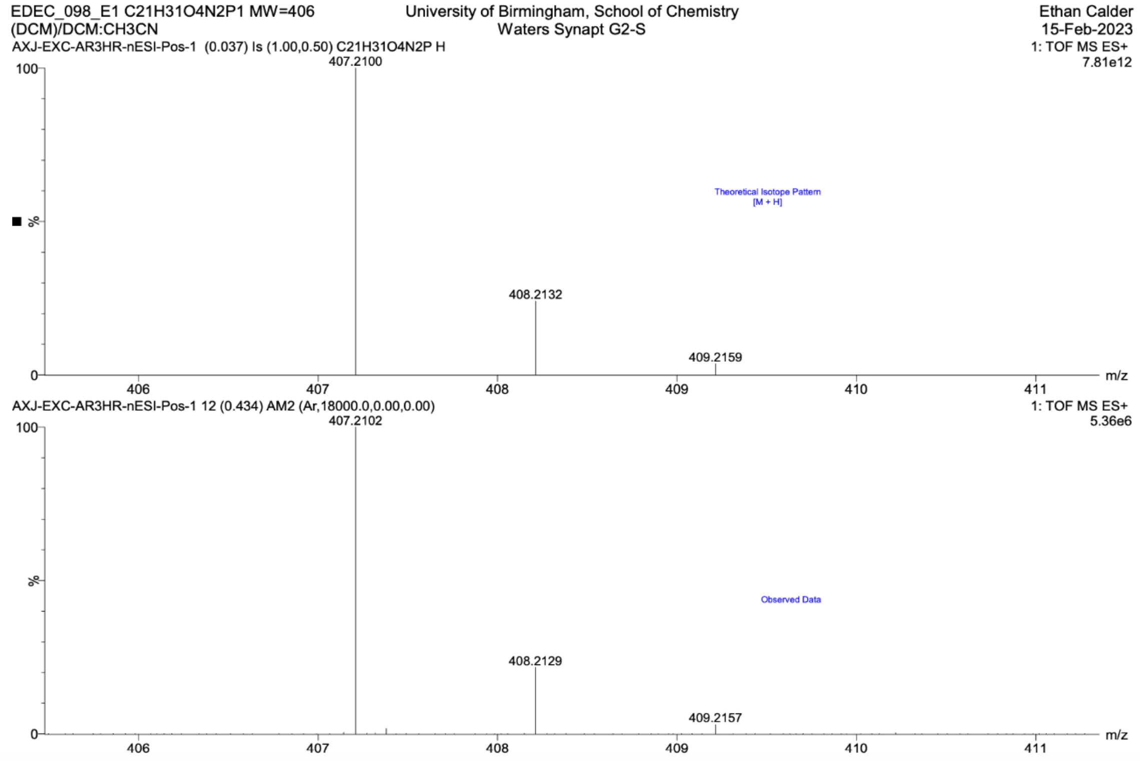

*Figure S60. High-res mass spectrum of **3** in dichloromethane. Top shows the theoretical isotope pattern based on {M + H}$^+$; bottom shows the observed data.*

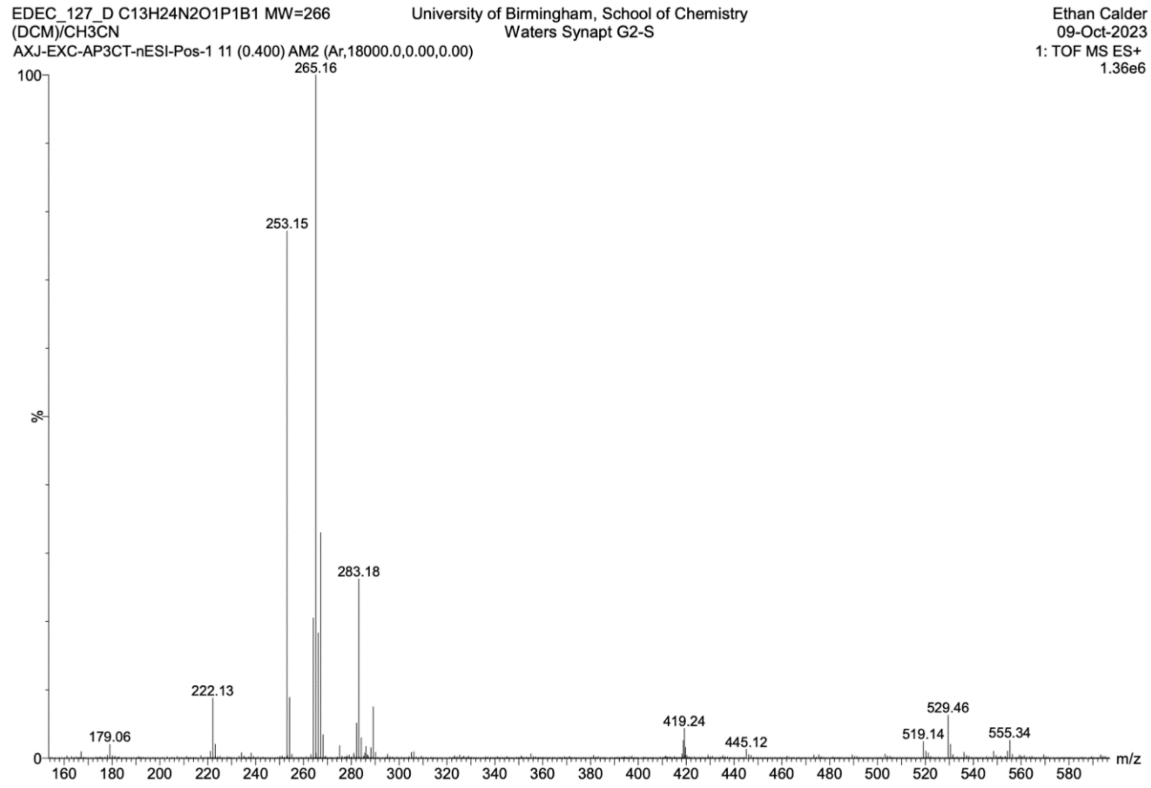

*Figure S61. Mass spectrum of **4** in dichloromethane.*

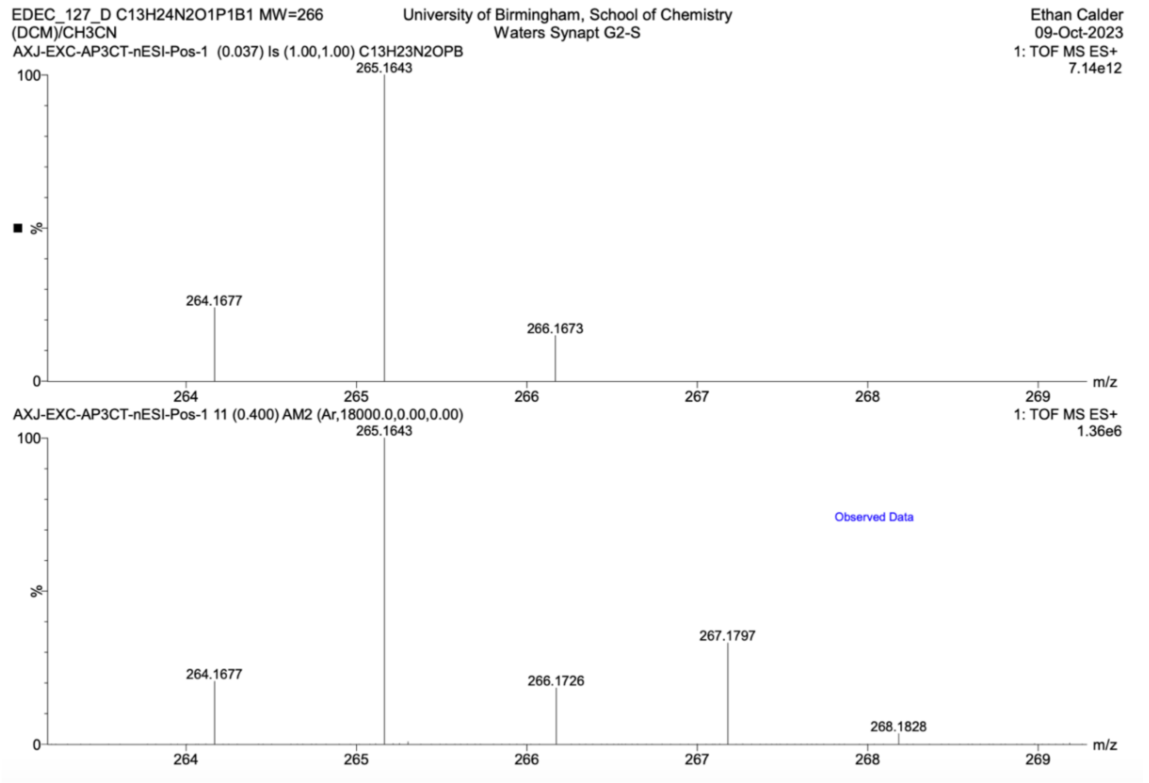

*Figure S62. High-res mass spectrum of **4** in dichloromethane. Top shows the theoretical isotope pattern based on {M - H}$^+$; bottom shows the observed data.*

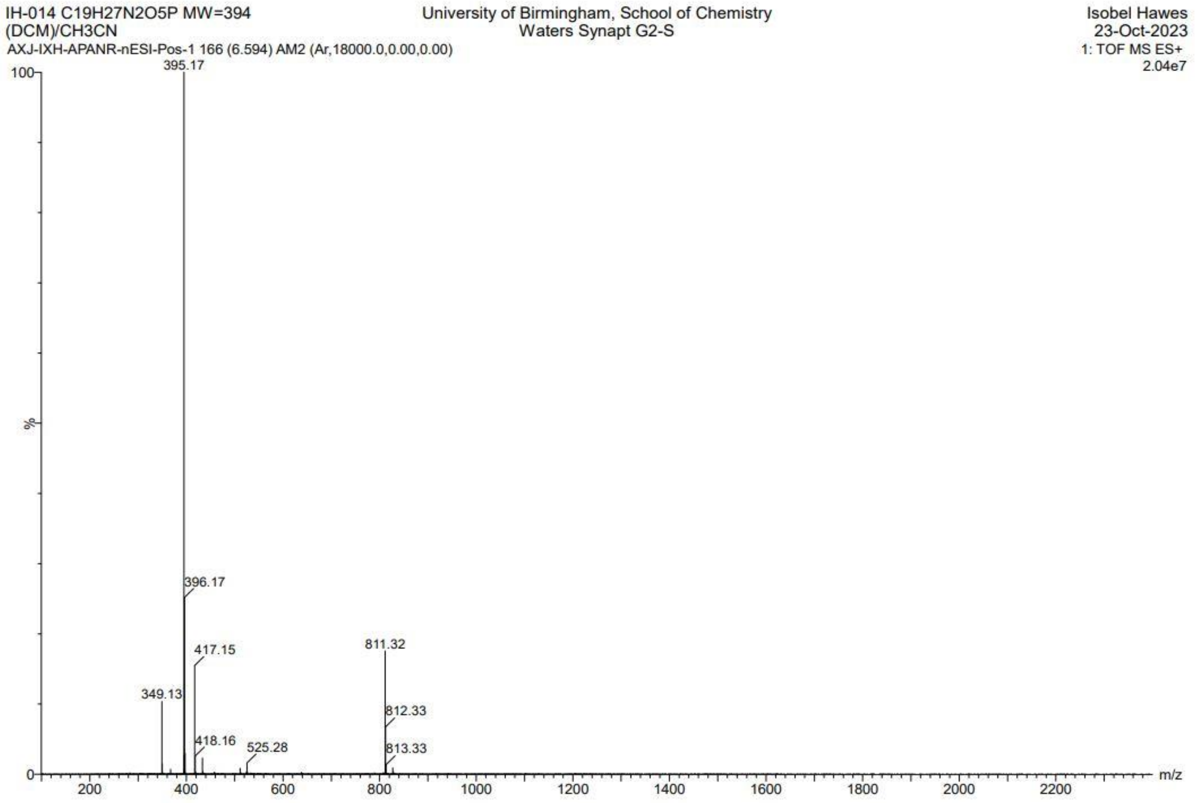

*Figure S63. Mass spectrum of **5** in dichloromethane.*

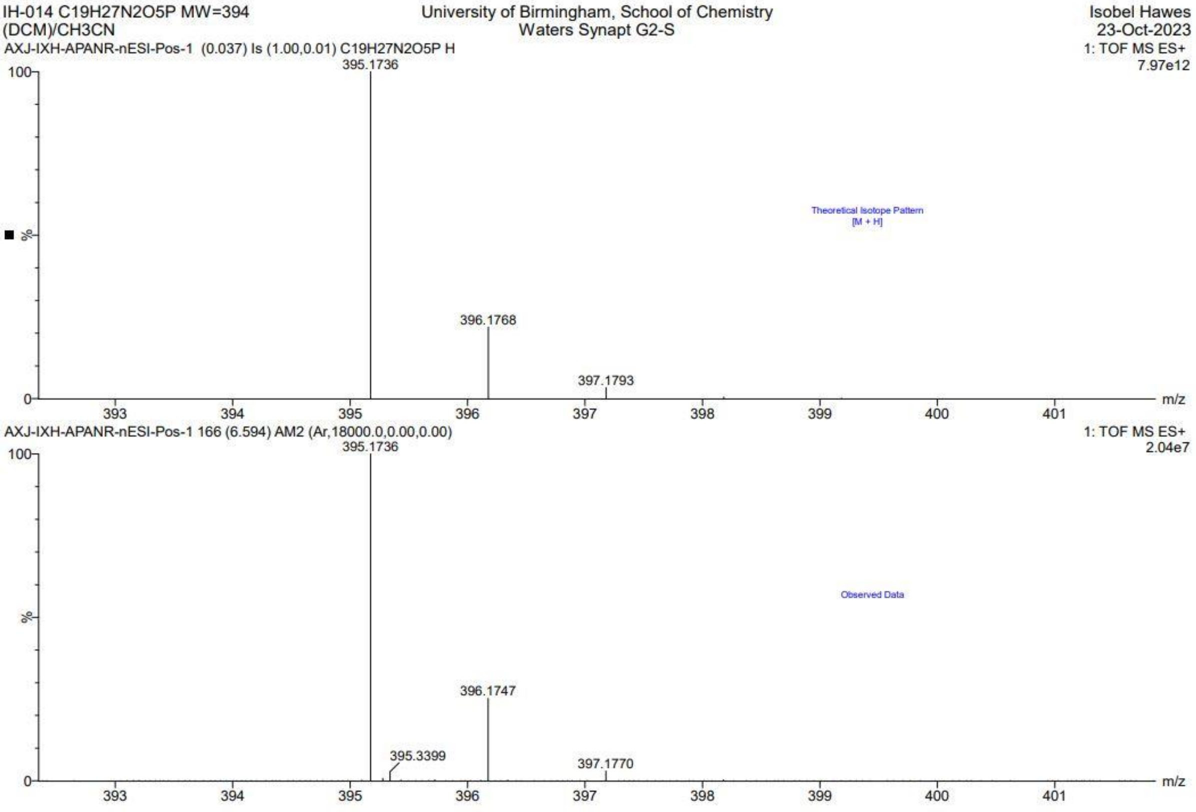

*Figure S64. High-res mass spectrum of **5** in dichloromethane. Top shows the theoretical isotope pattern based on {M - H}$^+$; bottom shows the observed data.*

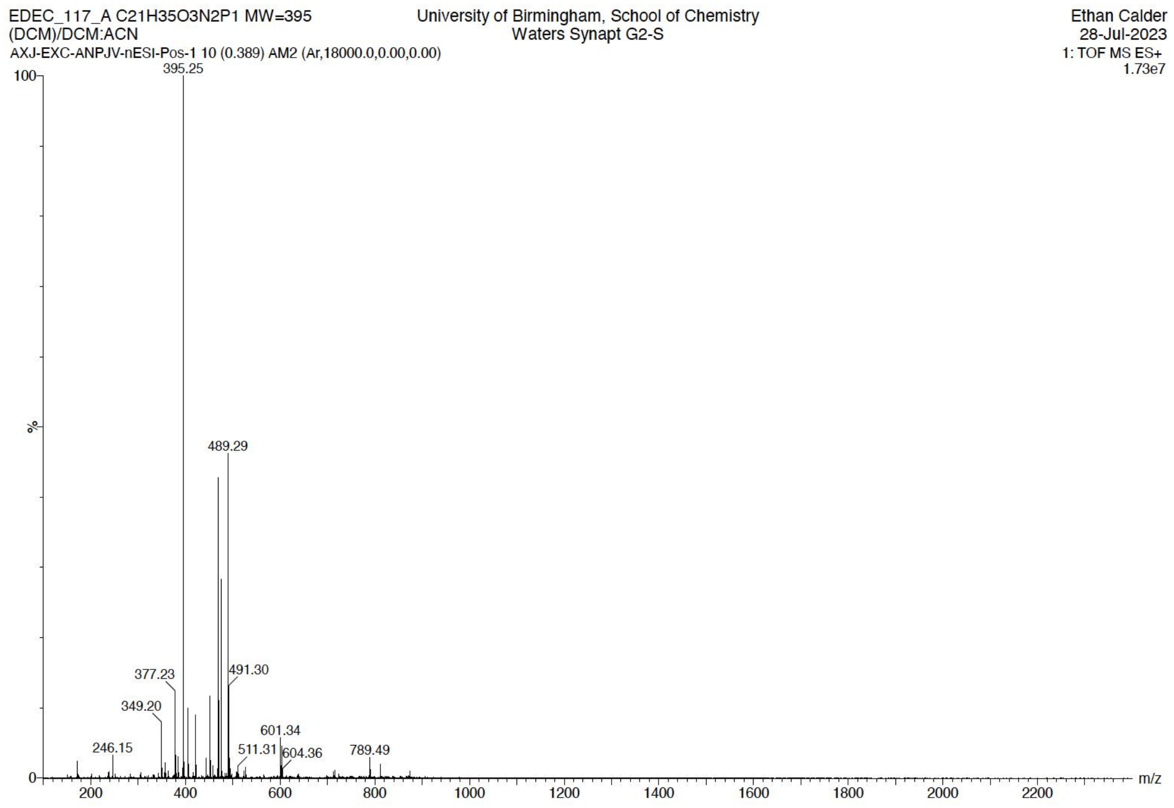

*Figure S65. Mass spectrum of **7** in dichloromethane.*

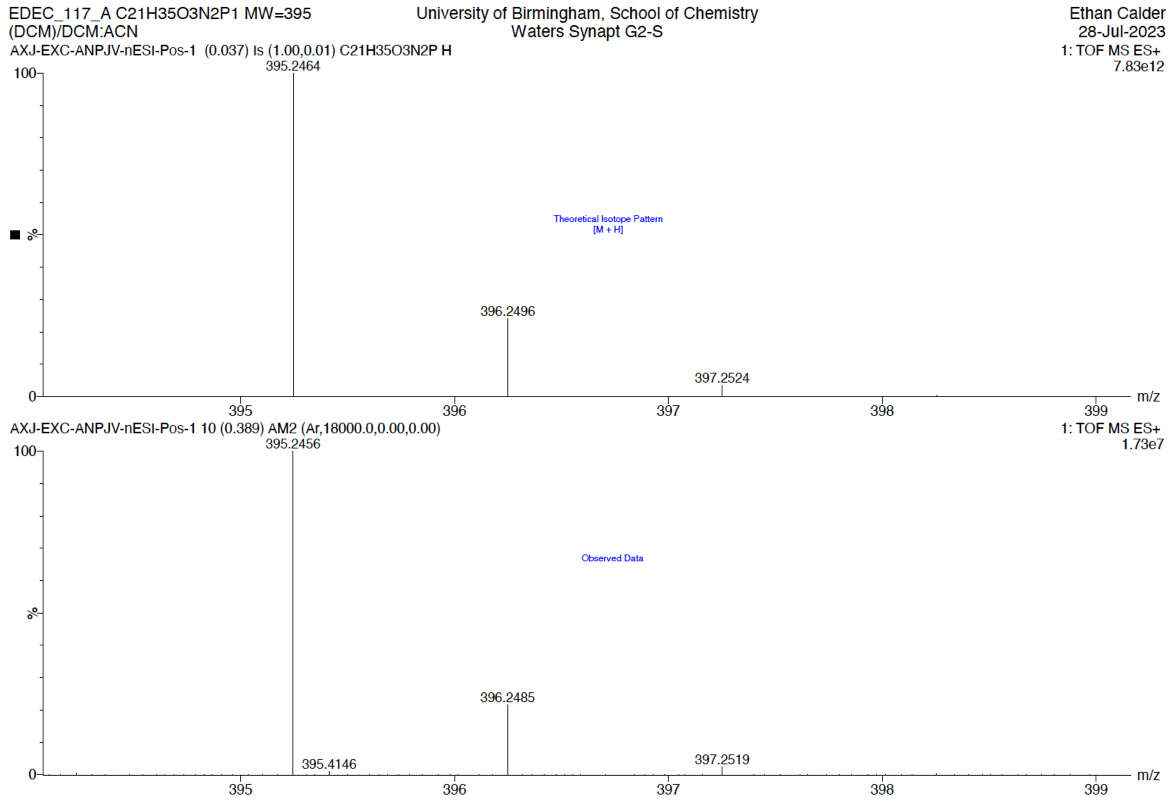

*Figure S66. High-res mass spectrum of **7** in dichloromethane. Top shows the theoretical isotope pattern based on {M + H}$^{+}$; bottom shows the observed data.*

S3.4. Attempts to characterise a complex between **3** and Al^{3+} with mass spectrometry

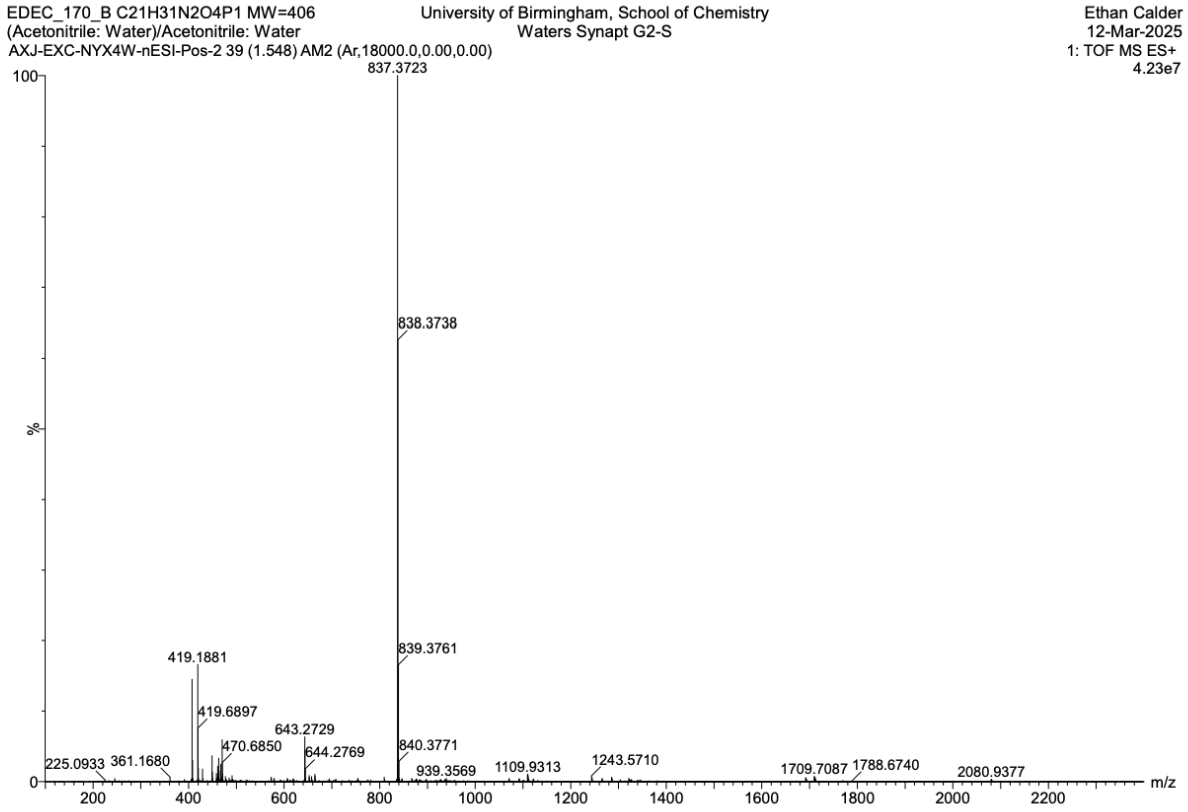

*Figure S67. Mass spectrum of a possible complex of **3** and Al^{3+} in acetonitrile:water.*

To make the sample, 1 equivalent of **3** (5.0 mg, 0.012 mmol) was mixed with 1.5 equiv of $AlCl_3.6H_2O$ (4.5 mg, 0.018 mmol) in 0.5 mL of a 1:1 mixture of acetonitrile:water. A drop of the resulting solution was then transferred to a mass spectrometry vial for analysis. A possible 2:1 complex between **3** and Al^{3+} is observed at m/z = 837.37, with free **3** also observed as a secondary peak at m/z = 407.21.

S3.5. IR Spectra

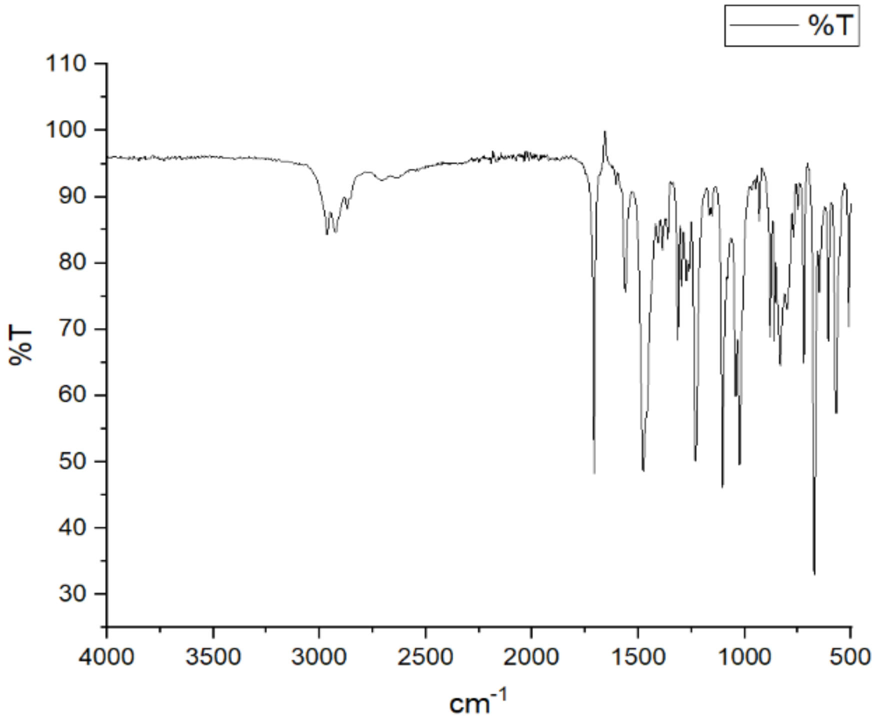

*Figure S68. IR Spectrum of **3**.*

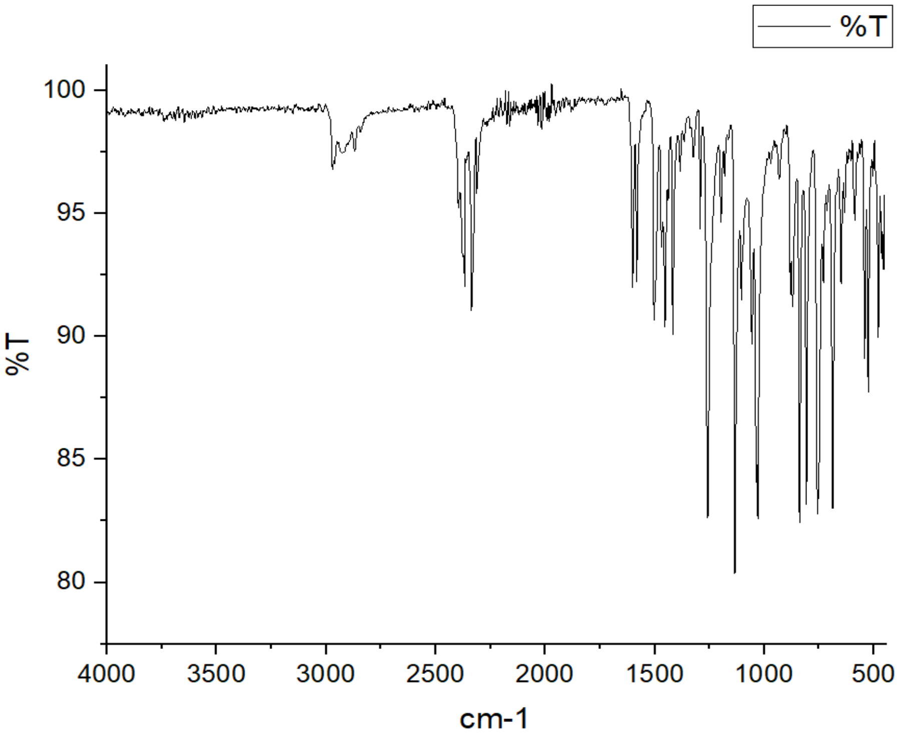

*Figure S69. IR Spectrum of **4**.*

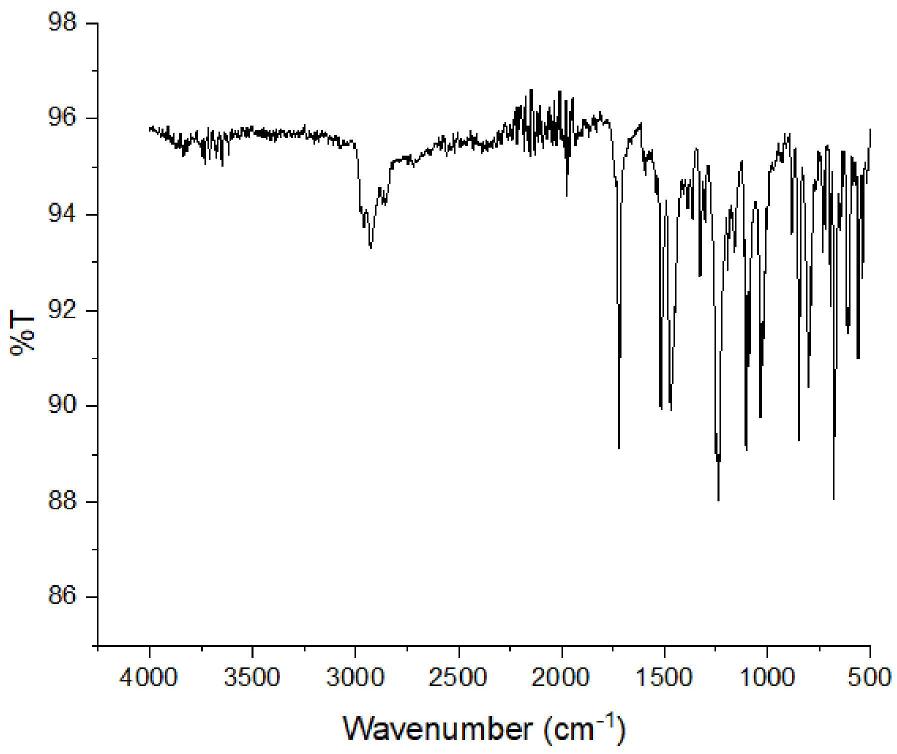

Figure S70. IR Spectrum of ***5.***

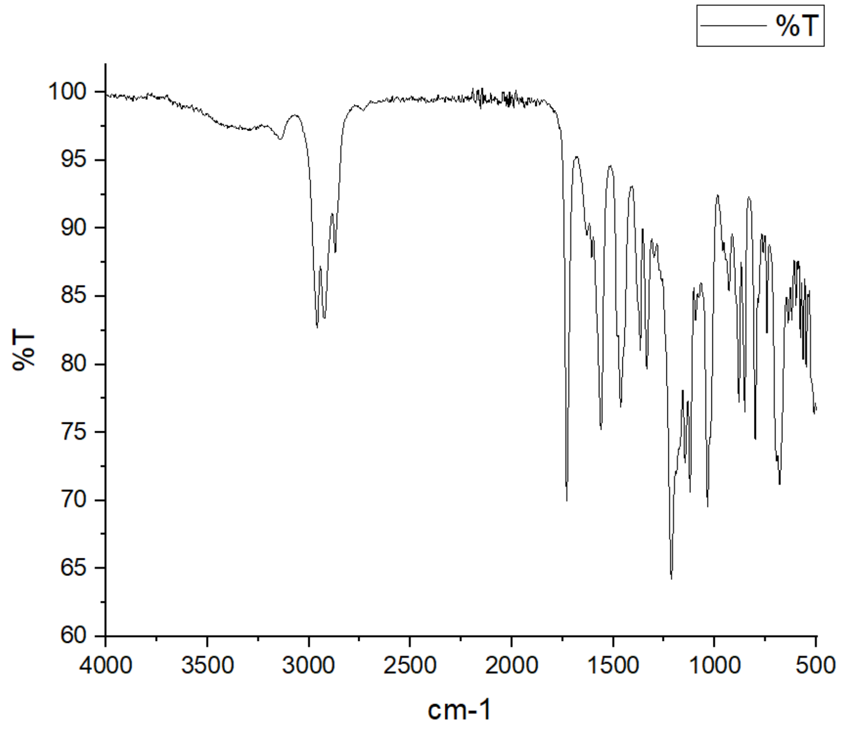

Figure S71. IR Spectrum of ***7****.*

S4. Crystallographic Details

S4.1. General Crystallographic Information

Single crystals suitable for X-ray diffraction were transferred from recrystallisation vials onto a microscope slide under oil. A suitable single crystal was then selected and mounted on the loop. The datasets for species **3α, 3β, 4, 5**, and **7** were measured using Cu-Kα radiation (λ = 1.54184) at either 100 K (for **3α** and **5**) or 120 K (for **3β, 4** and **7**) on a Rigaku SuperNova diffractometer using an Atlas detector. The data collections for these species were driven and processed and absorption corrections were applied using face indexing with a Gaussian absorption correction in CrysAlisPro.[2] Using OLEX2,[3] the structures were solved using ShelXT,[4] and were refined by a full-matrix least-squares procedure on F^2 in ShelXL.[5] In all structures, all non-hydrogen atoms were refined with anisotropic displacement parameters. Unless otherwise stated, all hydrogen atoms were fixed as riding models and the isotropic thermal parameters (U_{iso}) were based on the U_{eq} of the parent atom. Deposition numbers 2408101 (**3α**), 2408102 (**3β**), 2408098 (**4**), 2408099 (**5**), and 2408100 (**7**) contains the supplementary crystallographic data for this paper. These can be obtained free of charge by the joint Cambridge Crystallographic Data Centre and Fachinformationszentrum Karlsruhe via http://www.ccdc.cam.ac.uk/structures.

Single crystals of **3**, in the form of colourless plates (**3α**), were grown from slow evaporation of a concentrated *n*-hexane solution of **3**. The structure was solved in the monoclinic space group Cc with one molecule of **3**, no solvent molecules, and no disorder. A separate data set for **3** (**3β**) was collected in which the orientation of the P(=O)^{i}Pr$_2$ moiety is different; these single crystals were grown as colourless blocks from slow evaporation of a solution of **3** in THF:hexane (approximately 1:5 v/v). The structure was solved in the monoclinic space group P2$_1$/c with two molecules of **3**, one solvent (THF) molecule, and no disorder. Single crystals of **4**, in the form of red plates, were grown from slow evaporation of a $CDCl_3$ solution of **4** directly from a round-bottomed flask. The structure was solved in the monoclinic space group P2$_1$/c with one molecule of **4**, no solvent molecules, and no disorder. The hydrogen atoms bonded to B1 were located in the electron density and freely refined; all other hydrogen atoms were fixed as riding models. Single crystals of **5**, in the form of colourless rhombohedrons, were grown from slow evaporation of a concentrated *n*-hexane solution of **5**. The structure was solved in the monoclinic space group P2$_1$/n with one molecule of **5**, no solvent molecules, and no disorder. Single crystals of **7**, in the form of colourless plates, were grown from slow evaporation of a concentrated *n*-hexane solution of **7**. The structure was solved in the triclinic space group P-1 with two molecules of **7**, no solvent

molecules, and no disorder. The hydrogen atoms bonded to N2 and N102 were located in the electron density and freely refined; all other hydrogen atoms were fixed as riding models.

S4.2. Tables of Crystallographic Data

*Table S4. Crystallographic data and information for **3α, 3β, 4, 5,** and **7.***

Identification Code	**3α** – EDEC_098_D_gaussian	**3β** – EDEC_122_E_gaussian	**4** – EDEC_127_D_gaussian
CCDC Deposition Number	2408101	2408102	2408098
Empirical formula	$C_{21}H_{31}N_2O_4P$	$C_{46}H_{70}N_4O_9P_2$	$C_{13}H_{24}BN_2OP$
Formula weight	406.45	885.00	266.12
Temperature/K	99.95(15)	120.00(10)	120.01(10)
Crystal system	monoclinic	monoclinic	monoclinic
Space group	Cc	$P2_1/c$	$P2_1/c$
a/Å	9.5611(4)	21.6581(4)	6.9647(2)
b/Å	15.1064(5)	13.5091(2)	12.7140(4)
c/Å	16.1491(7)	16.8418(3)	17.5702(5)
α/°	90	90	90
β/°	105.972(4)	92.048(2)	95.093(2)
γ/°	90	90	90
Volume/Å³	2242.43(16)	4924.45(15)	1549.68(8)
Z	4	4	4
$\rho_{calc}g/cm^3$	1.204	1.194	1.141
μ/mm^{-1}	1.311	1.248	1.486
F(000)	872.0	1904.0	576.0
Crystal size/mm³	0.3 × 0.12 × 0.08	0.249 × 0.125 × 0.107	0.311 × 0.132 × 0.084
Radiation	Cu Kα (λ = 1.54184)	Cu Kα (λ = 1.54184)	Cu Kα (λ = 1.54184)
2Θ range for data collection/°	11.268 to 145.344	7.714 to 154.284	8.596 to 153.852
Index ranges	-11 ≤ h ≤ 11, -18 ≤ k ≤ 18, -19 ≤ l ≤ 19	-27 ≤ h ≤ 26, -10 ≤ k ≤ 16, -21 ≤ l ≤ 21	-6 ≤ h ≤ 8, -15 ≤ k ≤ 15, -22 ≤ l ≤ 22
Reflections collected	20414	49926	16058
Independent reflections	4069 [R_{int} = 0.0406, R_{sigma} = 0.0274]	10279 [R_{int} = 0.0501, R_{sigma} = 0.0385]	3235 [R_{int} = 0.0493, R_{sigma} = 0.0367]
Data/restraints/parameters	4069/2/262	10279/0/568	3235/0/180
Goodness-of-fit on F²	1.043	1.093	1.038
Final R indexes [I>=2σ (I)]	R_1 = 0.0357, wR_2 = 0.0924	R_1 = 0.0824, wR_2 = 0.2101	R_1 = 0.0399, wR_2 = 0.0967
Final R indexes [all data]	R_1 = 0.0375, wR_2 = 0.0942	R_1 = 0.0981, wR_2 = 0.2230	R_1 = 0.0535, wR_2 = 0.1071
Largest diff. peak/hole / e Å⁻³	0.15/-0.24	0.84/-0.53	0.29/-0.34
Flack Parameter	0.00(2)	N/A	N/A

Identification Code	**5** – IH_001_gaussian	**7** – EDEC_108_H_gaussian
CCDC Deposition Number	2408099	2408100
Empirical formula	$C_{19}H_{27}N_2O_5P$	$C_{21}H_{35}N_2O_3P$
Formula weight	394.39	394.48
Temperature/K	100.00(10)	120.00(10)
Crystal system	monoclinic	triclinic
Space group	$P2_1/n$	P-1
a/Å	8.4780(3)	11.9969(7)
b/Å	27.4558(8)	12.1442(6)
c/Å	8.8344(3)	16.8100(7)
α/°	90	77.509(4)
β/°	101.761(3)	87.508(4)
γ/°	90	69.254(5)
Volume/Å³	2013.21(12)	2234.6(2)
Z	4	4
$\rho_{calc}g/cm^3$	1.301	1.173
μ/mm^{-1}	1.485	1.262
F(000)	840.0	856.0
Crystal size/mm³	0.214 × 0.14 × 0.083	0.193 × 0.074 × 0.044
Radiation	Cu Kα (λ = 1.54184)	Cu Kα (λ = 1.54184)
2Θ range for data collection/°	10.724 to 154.27	7.886 to 145.36
Index ranges	-10 ≤ h ≤ 10, -34 ≤ k ≤ 34, -11 ≤ l ≤ 10	-14 ≤ h ≤ 14, -15 ≤ k ≤ 10, -20 ≤ l ≤ 18
Reflections collected	33018	15746
Independent reflections	4224 [R_{int} = 0.0582, R_{sigma} = 0.0293]	8391 [R_{int} = 0.0473, R_{sigma} = 0.0684]
Data/restraints/parameters	4224/0/251	8391/0/521
Goodness-of-fit on F^2	1.059	1.065
Final R indexes [I>=2σ (I)]	R_1 = 0.0432, wR_2 = 0.1042	R_1 = 0.0519, wR_2 = 0.1368
Final R indexes [all data]	R_1 = 0.0583, wR_2 = 0.1137	R_1 = 0.0793, wR_2 = 0.1519
Largest diff. peak/hole / e Å⁻³	0.41/-0.46	0.44/-0.36
Flack Parameter	N/A	N/A

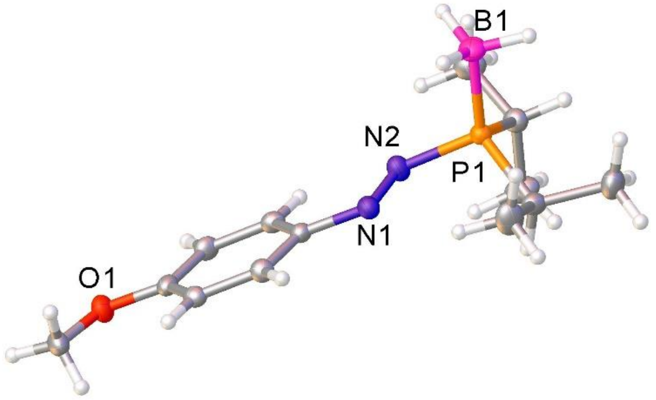

*Figure S72. Single crystal structure of **4**, with thermal ellipsoids drawn at the 50% probability level. Selected bond distance (Å): N1–N2 1.251(2), P1–N2 1.7362(14), P1–B1 1.921(2).*

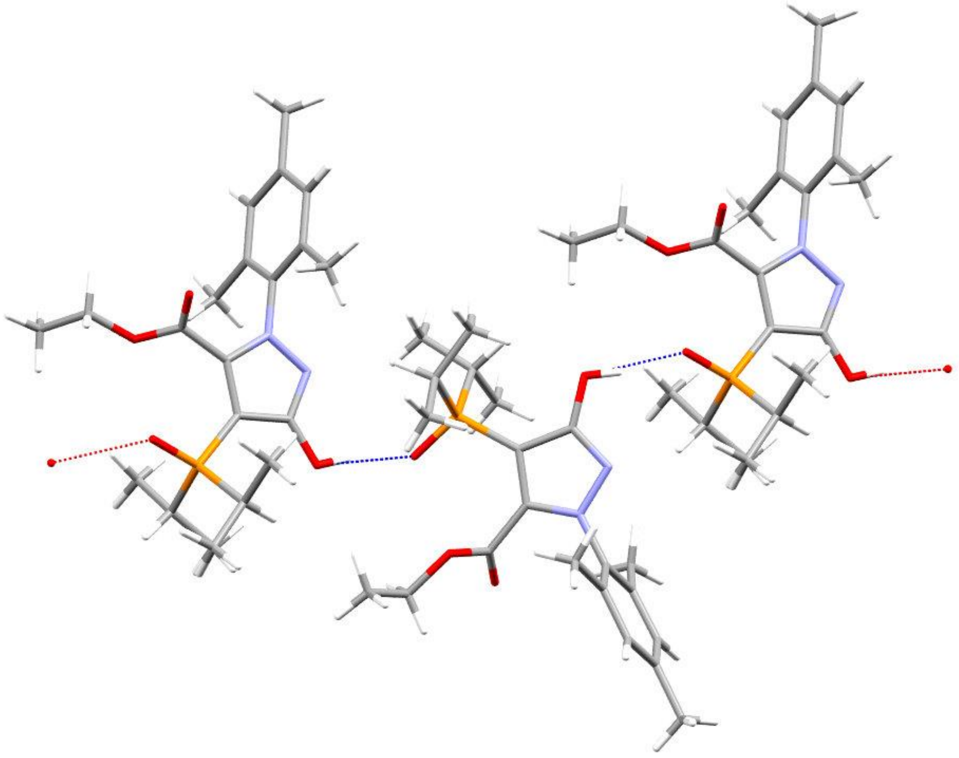

*Figure S73. Extended crystal structure of **3β**, highlighting the intermolecular hydrogen-bonding interactions between separate molecules of **3β** which leads to a coordination network. Blue dotted lines indicate intermolecular H-bonds, red dotted lines indicate where the coordination network continues to other molecules within the crystal (not shown here).*

S5. Computational Details

S5.1. General Computational Information

Unless otherwise stated, calculations for all complexes under study were performed using density functional theory (DFT) in Gaussian 16.[7] Geometry optimisations and frequency calculations were carried out using the ωB97XD functional and the def2-TZVP basis set,[8,9] with symmetry disabled (keyword = nosymm). Optimisations were carried out in the gas phase. Frequency calculations were then carried out on all optimised structures. For non-transition state structures, the absence of any imaginary frequencies confirmed that each optimised structure was located at a minimum. For transition state structures, the presence of exactly one imaginary frequency clearly corresponding to the transformation in question confirmed the structure as a valid transition state. Where necessary, IRC (intrinsic reaction coordinate) calculations were also performed to confirm the validity of transition states. Single point energy corrections were then carried out on these optimised structures using the ωB97XD functional and def2-QZVP basis set.[8,9] For single point energy corrections, solvent interactions were modelled using the polarisable continuum model (using toluene as the solvent, as toluene was also the solvent used experimentally, and is consistent with our previous publications in this area).[10] Where desired, TD-DFT calculations were carried out on the optimised geometries using the keyword TD(nstates=15) (see further details below), and NBO calculations (including Wiberg bond indices) were carried out on optimised geometries using the NBO package in Gaussian 16, using the keywords Pop=(FULL,NBOREAD,SAVENBO).[11] Full cartesian coordinates for all optimised structures can be found in section S5.3.

S5.2. Pertinent Energies and NBO Data on Structures

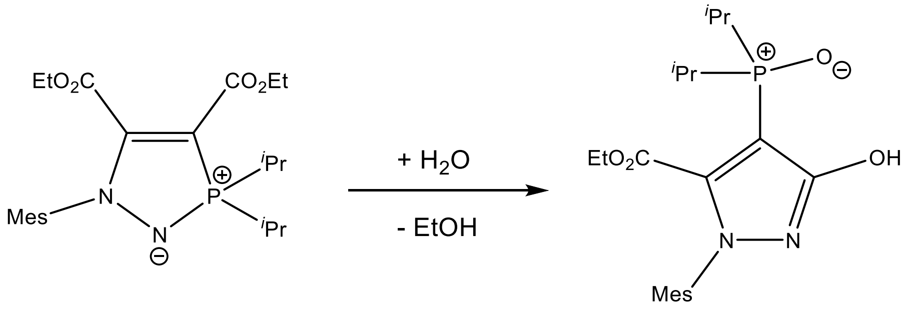

Pyrazolyl-phosphine oxide formation, R = ^{i}Pr:

*Table S5. Pertinent energies for all structures on the reaction pathway for the formation of **3** from **1**.*

Structure	*Single-point electronic energy (kcal.mol^{-1})*	*Thermal correction to Gibbs free energy (kcal.mol^{-1})*	*Total Gibbs energy (kcal.mol^{-1})*
Five-membered heterocycle **1**	-1035071.395	304.638968	-1034766.756
H_2O	-47973.18293	2.087724439	-47971.0952
EtOH	-97308.91021	34.64794256	-97274.26227
**TS1A***	-1131015.341	333.0193447	-1130682.321
**TS1B****	-1083027.607	319.5906393	-1082708.017
**I1**	-1083040.496	323.3400091	-1082717.156
**I2**	-1083066.475	323.495004	-1082742.98
**TS2**	-1083048.336	323.3036136	-1082725.032
**I3**	-1083071.317	323.0325294	-1082748.285
**I4**	-985759.4739	272.3554816	-985487.1184
Product **3**	-985777.6745	272.3347738	-985505.3397

* **TS1A** corresponds to the addition of two molecules of H_2O across the N-P bond, the most favourable pathway.

** **TS1B** corresponds to the addition of one molecule of H_2O across the N-P bond. This pathway is slightly less favourable than **TS1A** for this species, but is shown here for completeness.

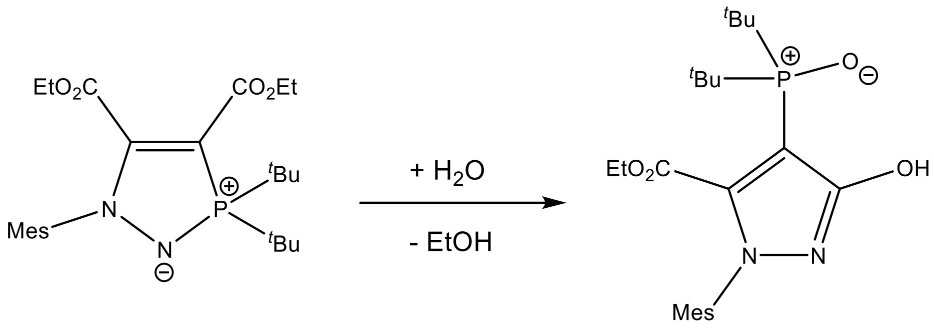

Pyrazolyl-phosphine oxide formation, R = ^{t}Bu:

*Table S6. Pertinent energies for all structures on the reaction pathway for the formation of a hypothetical pyrazolyl-phosphine oxide from **2**.*

Structure	*Single-point electronic energy (kcal.mol^{-1})*	*Thermal correction to Gibbs free energy (kcal.mol^{-1})*	*Total Gibbs energy (kcal.mol^{-1})*
Five-membered heterocycle **2**	-1084421.4	339.2229046	-1084082.177
H_2O	-47973.18293	2.087724439	-47971.0952
EtOH	-97308.91021	34.64794256	-97274.26227
**TS1A***	Not found	Not found	Not found
**TS1B****	-1132365.892	354.4280897	-1132011.464
**I1**	-1132379.776	358.0983934	-1132021.677
**I2**	-1132407.17	359.0089098	-1132048.161
**TS2**	-1132388.654	358.0293673	-1132030.625
**I3**	-1132411.467	358.2408381	-1132053.226
**I4**	-1035101.182	307.5995583	-1034793.583
Product, hypothetical pyrazolyl-phosphine oxide, R = ^{t}Bu	-1035119.426	308.3086442	-1034811.118

* **TS1A** corresponds to the addition of two molecules of H_2O across the N-P bond. For this species (R = ^{t}Bu), such a transition state could not be found; all attempts led to optimise this structure led to 'expulsion' of one H_2O molecule from the reactive site, with the structure instead reverting to TS1B (see below).

** **TS1B** corresponds to the addition of one molecule of H_2O across the N-P bond.

Pertinent NBO Data:

I2:

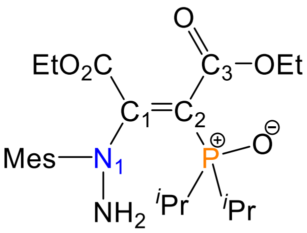

I3:

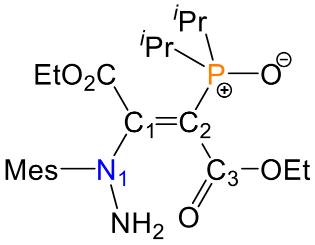

*NBO Data for species **I2** and **I3**:*

*Table S7. Pertinent NBO data for structures **I2** and **I3**.*

	**I2** (R = ^{i}Pr)	**I2** (R = ^{t}Bu)	**I3** (R = ^{i}Pr)	**I3** (R = ^{t}Bu)
Donation from N1 lp into C1=C2 π* orbital	65.25 kcal.mol^{-1}	61.63 kcal.mol^{-1}	67.44 kcal.mol^{-1}	63.90 kcal.mol^{-1}
Optimised bond length, and Wiberg bond index (WBI) for N1-C1 bond	1.35261, 1.2124	1.35587, 1.1970	1.36777, 1.1564	1.37011, 1.1536
Optimised bond length and WBI for C1-C2 bond	1.36915, 1.5649	1.37012, 1.5878	1.35300, 1.6452	1.35651, 1.6485
Optimised bond length and WBI for C2-P bond	1.82284, 0.8000	1.82848, 0.8014	1.81113, 0.8007	1.81963, 0.8098
Optimised bond length and WBI for C2-C3 bond	1.46710, 1.0619	1.47402, 1.0389	1.48550, 0.9913	1.49083, 0.9787

3α:

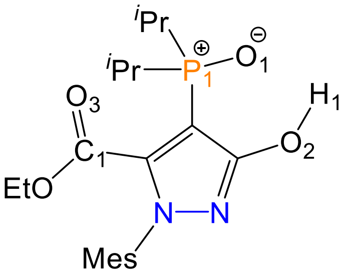

3β:

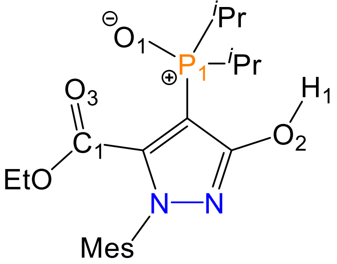

*NBO Data for species **3α** and **3β**:*

*Table S8. Pertinent NBO data for structures **3α** and **3β**.*

	**3α**	**3β**
Donation from O1 lp's into H1-O2 σ* orbital	21.32 kcal.mol^{-1}	N/A
Optimised interatomic distance between O1 and H1 (Å)	1.889	N/A
Donation from O1 lp's into C1=O3 π* orbital	N/A	1.53 kcal.mol^{-1}
Optimised interatomic distance between O1 and C1 (Å)	N/A	2.918

S5.3. TD-DFT Calculations on pyrazolyl-phosphine oxides

To probe the effect of the hydrogen bond between the phosphine oxide and alcohol functionalities on the fluorescence properties of pyrazolyl-phosphine oxides, TD-DFT calculations were performed on the optimised structures of **3α** and **3β**, using the following keywords:

#N wB97XD def2TZVP TD(nstates=15) nosymm

For **3α**, which features a hydrogen bond between the phosphine oxide and alcohol functionality, a HOMO to LUMO transition was observed at 269 nm in the gas phase. This is in very good agreement with the experimental absorbance value for **3** (274 nm in 1:1 MeCN:H_2O), which is the absorbance band which is excited in our fluorescence studies. The HOMO had contributions from the π-orbitals of the pyrazole ring, as well as from the oxygen lone pairs on the alcohol group. The LUMO mainly arose from π*-orbitals of the pyrazole ring. A secondary absorbance band at 237 nm was found which corresponded to a mixture of orbital transitions.

3β, which does not feature a hydrogen bond between the phosphine oxide and alcohol functionality, this absorbance band disappeared completely in our calculations. Only the secondary absorbance band was observed, at 233 nm. This shows that the interaction between the phosphine oxide and alcohol group is crucial for the luminescence properties of pyrazolyl-phosphine oxides, as only in the presence of this interaction was the absorbance band responsible for luminescence calculated.

S5.4. Cartesian coordinates for optimised structures

*Table S9. Cartesian coordinates of the optimised structure of **1**.*

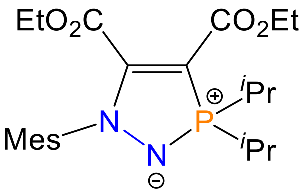

C	1.82719	2.05751	-0.99291
C	2.86722	1.16613	-0.36095
O	3.52989	1.92590	0.65656
C	4.71697	1.56565	1.14263
C	5.45369	0.46000	0.42760
C	5.26347	-0.91689	0.59657
C	4.27896	-1.57728	1.40801
O	3.54279	-0.71195	2.13115
C	2.51655	-1.25624	2.96185
C	1.79294	-0.09363	3.59691
O	4.09894	-2.77816	1.45001
P	6.44730	-1.67015	-0.45765
C	5.76050	-2.73333	-1.78405
C	5.41301	-4.16319	-1.37053
C	4.55603	-2.02235	-2.39862
C	7.77181	-2.58977	0.41816
C	7.31074	-3.63189	1.43452
C	8.80866	-3.12478	-0.56973
N	7.03557	-0.28938	-1.08788
N	6.39362	0.76485	-0.44795
C	6.90895	2.07643	-0.70100
C	6.45931	2.79395	-1.80172
C	5.43557	2.23203	-2.74560
C	6.99324	4.05992	-2.01493
C	7.95303	4.59868	-1.17238
C	8.40113	3.83281	-0.10158
C	7.89972	2.56639	0.15041
C	8.40553	1.73835	1.29440
O	5.18773	2.13109	2.08430

H	1.29037	1.50392	-1.76451
H	2.29079	2.93181	-1.45141
H	1.10878	2.40140	-0.24874
H	3.58757	0.81565	-1.10111
H	2.41010	0.29414	0.10965
H	2.97235	-1.90809	3.70942
H	1.84923	-1.86933	2.35274
H	2.47935	0.51296	4.18772
H	1.00196	-0.46343	4.25079
H	1.34277	0.54602	2.83663
H	6.56743	-2.75240	-2.52581
H	4.74029	-4.17073	-0.51225
H	6.29848	-4.74525	-1.11904
H	4.91691	-4.66795	-2.20223
H	3.72432	-1.99712	-1.69109
H	4.22584	-2.55517	-3.29216
H	4.79976	-0.99827	-2.68242
H	8.23179	-1.76431	0.97191
H	6.86259	-4.50266	0.95902
H	6.57574	-3.22295	2.12619
H	8.17338	-3.97287	2.01161
H	8.41664	-3.96224	-1.14989
H	9.68498	-3.48391	-0.02670
H	9.13681	-2.34765	-1.26244
H	5.53226	1.15006	-2.82781
H	5.55553	2.66382	-3.73886
H	4.42302	2.46501	-2.40875
H	6.65297	4.63304	-2.87059
C	8.49617	5.98152	-1.39938
H	9.16852	4.23167	0.55285
H	9.27882	2.20324	1.74989
H	8.68282	0.74077	0.94809
H	7.63340	1.62975	2.05807
H	8.28720	6.33029	-2.41073
H	9.57543	6.01208	-1.24305

H	8.04418	6.69075	-0.70180

*Table S10. Cartesian coordinates of the optimised structure of **2**.*

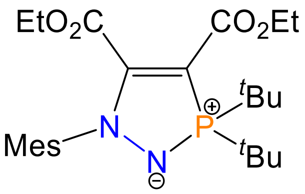

C	2.11254	-2.06779	-2.70873
C	3.53925	-1.61028	-2.87938
O	3.98500	-1.10660	-1.61204
C	5.20569	-0.58720	-1.58203
C	5.54128	-0.07535	-0.21185
C	6.19125	-0.77696	0.80536
C	6.70036	-2.11796	0.75361
O	6.31037	-2.78304	-0.35334
C	6.78275	-4.12086	-0.51521
C	5.89120	-5.11193	0.20212
O	7.41633	-2.62028	1.59863
P	6.39172	0.39552	2.10418
C	8.16412	0.78948	2.53001
C	8.23881	2.24814	3.00161
C	8.78865	-0.14614	3.56640
C	8.94258	0.65705	1.21402
C	5.39670	-0.06141	3.61543
C	3.93826	-0.08020	3.14106
C	5.74890	-1.44959	4.15825
C	5.54020	1.00434	4.70566
N	5.71466	1.65278	1.31599
N	5.28315	1.18370	0.08082
C	4.67401	2.11739	-0.81503
C	3.28804	2.23420	-0.84042
C	2.41781	1.44323	0.09168
C	2.72892	3.13184	-1.74304
C	3.51263	3.90469	-2.58700
C	4.89424	3.76842	-2.51885

C	5.49746	2.88208	-1.64027
C	6.98871	2.74008	-1.57533
O	5.94650	-0.50482	-2.52008
H	1.73810	-2.46109	-3.65463
H	1.47454	-1.23747	-2.40426
H	2.04313	-2.85374	-1.95625
H	3.62792	-0.81578	-3.62180
H	4.19940	-2.42645	-3.17821
H	7.80867	-4.18626	-0.15340
H	6.77344	-4.28752	-1.59207
H	5.92172	-4.94222	1.27813
H	6.23338	-6.12940	0.00423
H	4.85969	-5.02220	-0.14188
H	7.77184	2.39504	3.97480
H	7.75862	2.91736	2.28712
H	9.29031	2.53230	3.09251
H	8.35511	-0.01387	4.55741
H	9.85405	0.08670	3.64609
H	8.69021	-1.19084	3.27060
H	8.49803	1.25656	0.41970
H	8.98747	-0.37958	0.88051
H	9.96452	1.00963	1.37416
H	3.79068	-0.78556	2.32111
H	3.62548	0.90692	2.80520
H	3.29920	-0.38704	3.97268
H	5.63931	-2.21879	3.39478
H	5.06418	-1.68413	4.97823
H	6.76334	-1.51086	4.54388
H	5.35140	2.00560	4.31316
H	6.52594	0.99099	5.16976
H	4.80556	0.80959	5.49141
H	2.68221	0.38601	0.09211
H	1.36914	1.53734	-0.18865
H	2.53152	1.80975	1.11303
H	1.64947	3.22971	-1.78143

C	2.89056	4.88735	-3.53938
H	5.52059	4.36518	-3.17302
H	7.47381	3.42384	-2.27046
H	7.29098	1.72140	-1.82730
H	7.34344	2.95502	-0.56592
H	3.36383	4.83813	-4.52122
H	3.00821	5.90909	-3.17061
H	1.82417	4.69962	-3.66462

Table S11. Cartesian coordinates of the optimised structure of H_2O.

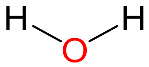

O	-2.52837	0.58957	0.00554
H	-1.57144	0.61935	-0.01151
H	-2.81500	1.38253	-0.44829

Table S12. Cartesian coordinates of the optimised structure of EtOH.

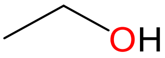

C	-2.47411	1.36316	-0.03391
C	-1.21523	0.51742	-0.08828
H	-3.35613	0.75470	-0.24410
H	-2.43880	2.16519	-0.77614
H	-2.58930	1.81451	0.95242
O	-0.04840	1.24715	0.23697
H	-1.11731	0.05001	-1.07648
H	-1.27026	-0.28753	0.64586
H	0.03887	1.97278	-0.38294

*Table S13. Cartesian coordinates of the optimised structure of **TS1A** (R = ⁱPr).*

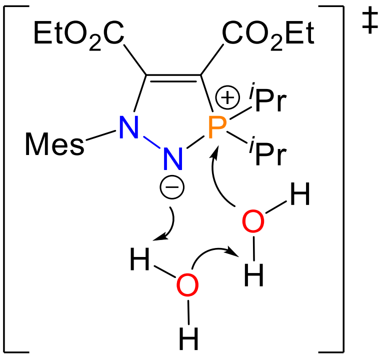

C	1.74761	1.82305	-0.82845
C	2.87280	0.96434	-0.30907
O	3.54702	1.70740	0.71689
C	4.74160	1.34149	1.16566
C	5.48897	0.28721	0.37904
C	5.40398	-1.07925	0.53354
C	4.60561	-1.76760	1.52347
O	3.71600	-0.95508	2.11812
C	2.96084	-1.48964	3.20942
C	2.06694	-0.38451	3.71609
O	4.71688	-2.93872	1.81204
P	6.53552	-1.85498	-0.61098
C	5.54365	-2.76208	-1.86382
C	4.35910	-3.51358	-1.26078
C	5.07954	-1.76746	-2.92540
C	7.78581	-2.86946	0.25646
C	8.02519	-2.36074	1.67587
C	7.46964	-4.36234	0.22452
N	7.12316	-0.36472	-1.05261
N	6.37913	0.69177	-0.51569
C	6.87234	2.01989	-0.72425
C	6.37259	2.76868	-1.78227
C	5.33572	2.22626	-2.72291
C	6.87107	4.05441	-1.95930
C	7.84679	4.57882	-1.12779
C	8.34654	3.78160	-0.10274
C	7.88308	2.49619	0.11455

C	8.45786	1.63454	1.19845
O	5.23188	1.85558	2.12567
H	1.20261	1.28154	-1.60266
H	2.13167	2.74936	-1.25726
H	1.05336	2.07657	-0.02746
H	3.57183	0.71212	-1.10757
H	2.50247	0.03757	0.13122
H	3.65224	-1.83734	3.97883
H	2.39064	-2.35185	2.85949
H	2.65793	0.47425	4.03479
H	1.48429	-0.74260	4.56571
H	1.37492	-0.05633	2.93929
H	6.24760	-3.44614	-2.33071
H	3.61855	-2.82738	-0.84486
H	4.64889	-4.20613	-0.47363
H	3.87212	-4.08178	-2.05582
H	4.43625	-0.99585	-2.49166
H	4.49478	-2.29618	-3.68022
H	5.92758	-1.29321	-3.41629
H	8.66905	-2.67684	-0.35095
H	7.18148	-2.59122	2.32572
H	8.20014	-1.28516	1.70012
H	8.91158	-2.85254	2.08105
H	6.56851	-4.58383	0.79735
H	8.29808	-4.90929	0.67842
H	7.34945	-4.72777	-0.79516
H	5.42408	2.70383	-3.69805
H	4.32758	2.42577	-2.35199
H	5.44182	1.15124	-2.86243
H	6.49273	4.65261	-2.78059
C	8.36830	5.97407	-1.32321
H	9.12660	4.17213	0.54125
H	7.68550	1.33609	1.90930
H	9.23445	2.16908	1.74292
H	8.89683	0.73162	0.76998

H	9.45810	5.97875	-1.38053
H	8.08217	6.61303	-0.48489
H	7.97735	6.42159	-2.23652
H	7.58574	-0.13978	-1.98265
O	8.37077	-0.06702	-3.33781
H	7.97403	0.10714	-4.19010
H	8.35977	-1.19285	-3.13034
O	8.16245	-2.34315	-2.62190
H	8.99166	-2.81781	-2.57641

*Table S14. Cartesian coordinates of the optimised structure of **TS1B** (R = ^{i}Pr).*

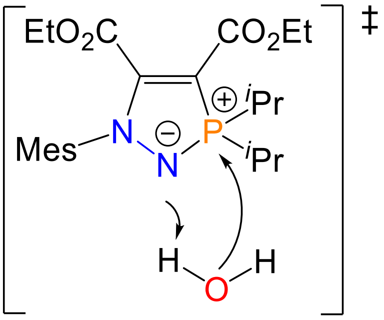

C	1.69001	1.67617	-0.93683
C	2.85824	0.88134	-0.41022
O	3.48931	1.66543	0.61115
C	4.69287	1.35392	1.08146
C	5.49700	0.31791	0.32856
C	5.44697	-1.05374	0.48010
C	4.63415	-1.75736	1.44691
O	3.72861	-0.95722	2.03846
C	2.95334	-1.51287	3.10351
C	2.03781	-0.42247	3.60403
O	4.74517	-2.93089	1.72592
P	6.64588	-1.79072	-0.63390
C	5.71309	-2.69401	-1.93622
C	4.53866	-3.50376	-1.38754
C	5.22797	-1.69610	-2.98529
C	7.86319	-2.84088	0.23090
C	8.14961	-2.29764	1.62802

C	7.49120	-4.32158	0.26254
N	7.21411	-0.28293	-0.99594
N	6.42123	0.75418	-0.52362
C	6.88190	2.09604	-0.70454
C	6.35574	2.86107	-1.73816
C	5.30717	2.32875	-2.67235
C	6.82639	4.16158	-1.89027
C	7.79652	4.68788	-1.05406
C	8.31486	3.88023	-0.04583
C	7.87859	2.58157	0.14735
C	8.45560	1.71861	1.22862
O	5.14878	1.91078	2.03497
H	1.17534	1.10204	-1.70833
H	2.02427	2.61921	-1.37093
H	0.98214	1.89737	-0.13812
H	3.57178	0.66153	-1.20540
H	2.53516	-0.06102	0.03385
H	3.62897	-1.86562	3.88465
H	2.39862	-2.37523	2.72954
H	2.61346	0.43692	3.94814
H	1.43810	-0.79694	4.43456
H	1.36199	-0.08896	2.81528
H	6.44634	-3.35856	-2.39120
H	3.76627	-2.84916	-0.97930
H	4.82864	-4.19976	-0.60397
H	4.09122	-4.07194	-2.20577
H	4.51668	-0.98965	-2.54795
H	4.70928	-2.22799	-3.78518
H	6.05789	-1.13987	-3.41769
H	8.73340	-2.70331	-0.41461
H	7.30391	-2.46129	2.29700
H	8.37806	-1.23223	1.61126
H	9.01534	-2.81865	2.04133
H	6.59794	-4.49174	0.86425
H	8.31455	-4.87918	0.71313

H	7.33312	-4.72633	-0.73738
H	5.38742	1.24997	-2.79884
H	5.39914	2.79513	-3.65278
H	4.30574	2.55255	-2.29713
H	6.42505	4.77158	-2.69189
C	8.28571	6.09876	-1.21965
H	9.08257	4.27661	0.60947
H	7.67059	1.36450	1.89913
H	9.18365	2.27440	1.81718
H	8.95088	0.84756	0.79616
H	7.98564	6.71244	-0.36746
H	7.88314	6.55705	-2.12256
H	9.37506	6.13071	-1.27725
H	7.83103	-0.40770	-1.88806
O	8.38436	-1.70285	-2.37543
H	8.30327	-2.00898	-3.27822

*Table S15. Cartesian coordinates of the optimised structure of **TS1B** (R = ^{t}Bu).*

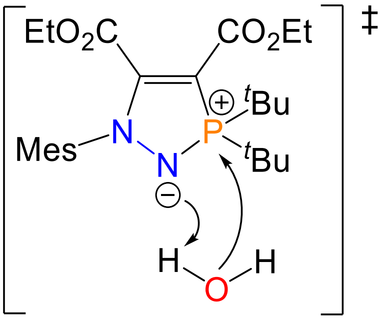

C	1.82936	1.94707	-0.86528
C	2.89206	1.04286	-0.29248
O	3.57450	1.77034	0.73548
C	4.78452	1.42351	1.17114
C	5.54953	0.38026	0.38634
C	5.37751	-0.98860	0.39058
C	4.30365	-1.61248	1.14425
O	3.84530	-0.80629	2.12093

C	2.68396	-1.23319	2.83632
C	2.27717	-0.09890	3.74509
O	3.82099	-2.70473	0.94540
P	6.66636	-1.74996	-0.65441
C	5.80720	-2.72854	-2.00447
C	5.31319	-4.08208	-1.47566
C	4.59009	-1.90293	-2.44377
C	7.71234	-2.74458	0.56106
C	6.82762	-3.34905	1.66028
C	8.53494	-3.86821	-0.07360
N	7.07365	-0.20762	-1.15481
N	6.50934	0.82489	-0.41554
C	6.92065	2.16776	-0.68260
C	6.38683	2.84456	-1.77582
C	5.37176	2.21512	-2.68561
C	6.81587	4.14515	-2.01222
C	7.74886	4.76558	-1.19682
C	8.27766	4.04768	-0.13015
C	7.88915	2.74622	0.14222
C	8.50250	1.98083	1.27573
O	5.27802	1.97509	2.10838
H	1.27882	1.41667	-1.64327
H	2.27413	2.84199	-1.30200
H	1.12656	2.25640	-0.09177
H	3.59505	0.72209	-1.06157
H	2.44814	0.15054	0.15365
H	2.92516	-2.13975	3.39394
H	1.89702	-1.48668	2.12226
H	3.07530	0.13926	4.44811
H	1.38770	-0.38049	4.31026
H	2.05503	0.79911	3.16759
C	6.69926	-2.96660	-3.22239
H	4.67810	-3.97169	-0.60004
H	6.13591	-4.75545	-1.24574
H	4.72087	-4.55182	-2.26510

H	3.85516	-1.80911	-1.64530
H	4.11318	-2.41289	-3.28378
H	4.88105	-0.90696	-2.77907
C	8.65537	-1.72226	1.20791
H	6.05847	-4.01463	1.27318
H	6.35146	-2.58640	2.27533
H	7.47686	-3.93262	2.31687
H	7.90955	-4.68051	-0.44185
H	9.18113	-4.28909	0.70161
H	9.14775	-3.47450	-0.87884
H	5.49583	1.13510	-2.73753
H	5.46350	2.61756	-3.69395
H	4.35789	2.42947	-2.34092
H	6.40896	4.68222	-2.86194
C	8.17915	6.18300	-1.44731
H	9.02272	4.51332	0.50522
H	9.22906	2.59648	1.80370
H	9.01445	1.09092	0.90698
H	7.73874	1.66265	1.98628
H	9.26155	6.28675	-1.35932
H	7.72546	6.85567	-0.71571
H	7.88250	6.52008	-2.44029
H	8.06600	-0.23570	-1.50752
O	8.80691	-1.60050	-1.77154
H	8.99208	-1.88352	-2.66543
H	9.34685	-1.32124	0.46896
H	9.22525	-2.22649	1.99241
H	8.10025	-0.90840	1.67939
H	6.11141	-3.50514	-3.97100
H	7.57001	-3.56861	-2.97112
H	7.03796	-2.02868	-3.66023

*Table S16. Cartesian coordinates of the optimised structure of **I1** (R = ^{i}Pr).*

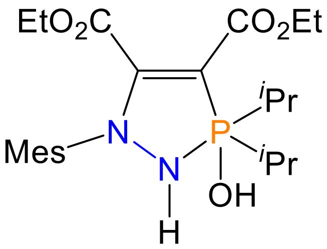

C	1.66830	1.84499	-1.00456
C	2.87871	1.04884	-0.58585
O	3.52119	1.76627	0.47433
C	4.71916	1.39451	0.92322
C	5.49330	0.37547	0.11975
C	5.51356	-0.97025	0.24915
C	4.62277	-1.70787	1.11913
O	3.75217	-0.92301	1.78317
C	2.85892	-1.55405	2.70057
C	1.96831	-0.47567	3.26858
O	4.62238	-2.91428	1.25199
P	6.80019	-1.76525	-0.88510
C	5.58189	-2.75110	-1.94321
C	5.47155	-4.23837	-1.60671
C	4.19519	-2.12472	-2.06004
C	7.89256	-2.51606	0.45293
C	7.63831	-2.09810	1.89794
C	8.05889	-4.03413	0.38540
N	7.13889	-0.16211	-1.32322
N	6.33152	0.86454	-0.82581
C	6.89263	2.18268	-0.84576
C	6.40721	3.09167	-1.78068
C	5.29480	2.74156	-2.72857
C	6.96708	4.36536	-1.81322
C	7.98660	4.73442	-0.95228
C	8.45544	3.79511	-0.03875
C	7.92685	2.51811	0.03611
C	8.42697	1.53628	1.05236
O	5.18832	1.89798	1.90103

H	1.14406	1.32053	-1.80453
H	1.95821	2.83162	-1.36821
H	0.98283	1.97555	-0.16709
H	3.57042	0.92212	-1.41919
H	2.59951	0.06077	-0.21784
H	3.44197	-2.05008	3.47887
H	2.28799	-2.32233	2.17548
H	2.56113	0.29186	3.76626
H	1.27771	-0.90951	3.99299
H	1.38533	0.00196	2.47981
H	6.03899	-2.66200	-2.92933
H	5.09445	-4.38343	-0.59528
H	6.42452	-4.75932	-1.69854
H	4.77524	-4.71164	-2.30357
H	3.59519	-2.27247	-1.16289
H	3.66602	-2.59952	-2.89029
H	4.24899	-1.05585	-2.26993
H	8.84017	-2.06851	0.14231
H	6.78465	-2.62164	2.32490
H	7.47521	-1.02728	2.00198
H	8.51828	-2.35822	2.49185
H	7.13748	-4.54198	0.67024
H	8.83727	-4.34015	1.08807
H	8.36332	-4.38877	-0.59950
H	5.30609	1.68543	-2.99633
H	5.36925	3.33210	-3.64138
H	4.32386	2.95761	-2.27622
H	6.59533	5.08179	-2.53750
C	8.57217	6.11784	-0.98534
H	9.25444	4.07009	0.64126
H	9.29126	1.93504	1.58135
H	8.70832	0.59285	0.58325
H	7.64277	1.32898	1.78341
H	8.22356	6.67771	-1.85289
H	9.66240	6.08196	-1.01742

H	8.29015	6.67601	-0.08974
H	7.56712	0.03570	-2.21030
O	8.06893	-2.09793	-2.05183
H	8.12142	-3.02502	-2.28121

*Table S17. Cartesian coordinates of the optimised structure of **I1** (R = ^{t}Bu).*

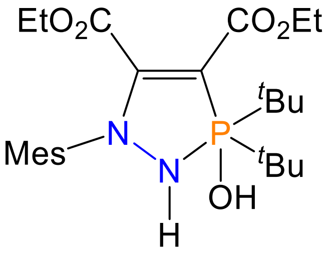

C	1.90048	1.90964	-0.93438
C	2.94392	0.99140	-0.34763
O	3.61424	1.70370	0.69662
C	4.83575	1.37735	1.12178
C	5.62957	0.36288	0.32888
C	5.47855	-0.99052	0.25533
C	4.37763	-1.61301	0.97823
O	3.98092	-0.87230	2.03732
C	2.80395	-1.29093	2.72733
C	2.47117	-0.21833	3.73623
O	3.81884	-2.65352	0.70690
P	6.87394	-1.77458	-0.80724
C	5.79196	-2.68921	-2.09608
C	5.42399	-4.09300	-1.60142
C	4.50488	-1.89539	-2.35703
C	7.66548	-2.74745	0.65179
C	6.69288	-3.30811	1.69360
C	8.57718	-3.90523	0.21185
N	7.23219	-0.13914	-1.16090
N	6.63226	0.86065	-0.40633
C	6.97997	2.21658	-0.67785
C	6.45076	2.86148	-1.79456
C	5.48600	2.17713	-2.71963
C	6.83270	4.17545	-2.03535

C	7.71466	4.84432	-1.20016
C	8.24104	4.16201	-0.10977
C	7.89792	2.84772	0.16672
C	8.49565	2.12528	1.33752
O	5.31109	1.93700	2.06573
H	1.35726	1.38838	-1.72374
H	2.36270	2.80133	-1.36000
H	1.18808	2.22438	-0.17183
H	3.65651	0.66491	-1.10422
H	2.48214	0.10024	0.08301
H	2.99371	-2.25600	3.20119
H	1.99592	-1.43544	2.00612
H	3.28539	-0.09185	4.44986
H	1.56829	-0.49316	4.28348
H	2.30343	0.73842	3.24052
C	6.49812	-2.82366	-3.45382
H	4.86784	-4.05844	-0.66818
H	6.30423	-4.72516	-1.47798
H	4.78704	-4.57171	-2.35087
H	3.84996	-1.87339	-1.49230
H	3.95901	-2.37762	-3.17186
H	4.72503	-0.87247	-2.66798
C	8.53811	-1.69117	1.34988
H	5.96810	-4.00073	1.27082
H	6.15933	-2.52494	2.22689
H	7.27476	-3.84956	2.44376
H	8.05844	-4.63626	-0.41056
H	8.92140	-4.43882	1.10095
H	9.46283	-3.55417	-0.31324
H	5.68149	1.10768	-2.78162
H	5.55416	2.59797	-3.72240
H	4.45863	2.31744	-2.37712
H	6.42858	4.68636	-2.90256
C	8.08480	6.27820	-1.45432
H	8.94571	4.66651	0.54200

H	9.24587	2.74426	1.82770
H	8.97158	1.19454	1.02477
H	7.72143	1.87232	2.06289
H	7.89230	6.56192	-2.48896
H	9.13877	6.45844	-1.23923
H	7.50104	6.94262	-0.81264
H	8.17207	0.08092	-1.44276
O	8.34694	-1.97617	-1.71051
H	8.49940	-2.87546	-1.99178
H	9.29350	-1.28722	0.67446
H	9.05352	-2.15511	2.19535
H	7.93464	-0.86974	1.74072
H	5.77254	-3.19680	-4.18093
H	7.31798	-3.54321	-3.43870
H	6.88600	-1.87117	-3.81275

Table S18. Cartesian coordinates of the optimised structure of ***I2*** *(R = ^{i}Pr).*

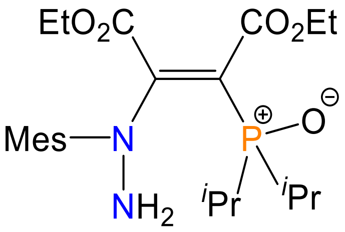

N	1.43422	-0.80075	-0.16459
N	2.73357	-0.26259	-0.17075
C	3.00922	0.82999	-1.05420
C	3.24690	2.09632	-0.51051
C	3.25214	2.36083	0.96842
C	3.47718	3.15223	-1.38600
C	3.45220	2.98905	-2.76055
C	3.16150	1.72730	-3.26336
C	2.93413	0.63915	-2.43762
C	2.61027	-0.69445	-3.04557
C	3.74521	4.13578	-3.68648
C	3.50155	-0.62556	0.88187
C	2.67067	-0.99364	2.11161

O	2.96013	-2.22599	2.51230
C	2.31133	-2.67448	3.70751
C	2.85084	-4.04558	4.02950
O	1.87644	-0.26662	2.63593
C	4.86895	-0.67395	0.93140
P	5.93585	-1.02953	-0.50318
C	7.27970	0.20919	-0.42078
C	8.41216	-0.09985	-1.39938
C	6.68567	1.59209	-0.68823
C	6.64649	-2.69798	-0.17519
C	7.14363	-3.29942	-1.49377
C	5.61371	-3.63330	0.45604
O	5.21482	-1.02233	-1.79931
C	5.54531	-0.71384	2.23268
O	4.83076	-0.12327	3.20584
C	5.34426	-0.21429	4.53578
C	4.40368	0.55148	5.43404
O	6.65025	-1.16627	2.43491
H	1.30278	-1.34433	-1.00745
H	0.75873	-0.04568	-0.14732
H	2.45090	1.83298	1.48587
H	3.13984	3.42803	1.15615
H	4.18533	2.03537	1.43422
H	3.67216	4.13508	-0.97094
H	3.12492	1.57967	-4.33740
H	3.05135	-0.77093	-4.03817
H	1.52853	-0.82195	-3.15726
H	3.02350	-1.50416	-2.44771
H	3.63101	5.09458	-3.18031
H	3.08223	4.12523	-4.55283
H	4.77099	4.07674	-4.05890
H	1.23308	-2.68471	3.53975
H	2.51821	-1.95742	4.50455
H	2.64521	-4.74408	3.21816
H	2.37909	-4.42230	4.93791

H	3.92915	-4.01015	4.18908
H	7.67097	0.16972	0.59840
H	8.03152	-0.21915	-2.41578
H	8.95556	-1.00342	-1.12298
H	9.12735	0.72550	-1.40323
H	6.18907	1.62349	-1.66010
H	7.47606	2.34564	-0.67866
H	5.95116	1.86833	0.06775
H	7.47692	-2.56461	0.51974
H	6.31335	-3.42791	-2.18851
H	7.58778	-4.27809	-1.29810
H	7.89215	-2.68452	-1.98990
H	4.70841	-3.68217	-0.15427
H	5.32952	-3.32285	1.45904
H	6.02701	-4.64257	0.51715
H	6.35510	0.19521	4.55789
H	5.40934	-1.26828	4.81728
H	4.36972	1.60360	5.15035
H	4.74300	0.48220	6.46841
H	3.39091	0.15215	5.36978

*Table S19. Cartesian coordinates of the optimised structure of **I2** (R = ^{t}Bu).*

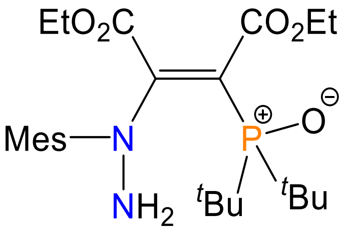

N	1.47039	-0.88930	-0.25718
N	2.75197	-0.31296	-0.19178
C	3.02485	0.81362	-1.03036
C	3.20456	2.07087	-0.44499
C	3.16441	2.29190	1.04029
C	3.42466	3.15998	-1.28207
C	3.44813	3.03803	-2.66050

C	3.21805	1.78223	-3.20790
C	3.00095	0.66324	-2.42141
C	2.74055	-0.65990	-3.08105
C	3.72949	4.22100	-3.54346
C	3.47492	-0.69108	0.89115
C	2.58141	-1.06289	2.07790
O	2.89952	-2.26916	2.53502
C	2.22413	-2.70418	3.72081
C	2.87392	-3.99166	4.16236
O	1.73276	-0.35607	2.54177
C	4.83550	-0.77107	1.03138
P	5.97550	-1.05150	-0.37044
C	7.28376	0.26304	-0.30789
C	8.29934	-0.01867	-1.42302
C	6.55700	1.57393	-0.63746
C	6.66416	-2.79486	-0.22313
C	6.96855	-3.24082	-1.66482
C	5.53826	-3.68559	0.32067
C	5.35327	-0.89476	2.40591
O	4.68851	-0.08891	3.25377
C	4.97741	-0.23901	4.64538
C	4.03620	0.66896	5.39857
O	6.27234	-1.58391	2.77602
H	1.39357	-1.42053	-1.11427
H	0.77019	-0.15703	-0.25379
H	2.34781	1.74868	1.51577
H	3.04483	3.35311	1.25567
H	4.08266	1.95260	1.52536
H	3.57422	4.13557	-0.83245
H	3.21995	1.66532	-4.28643
H	3.21676	-0.69088	-4.06001
H	1.66801	-0.81683	-3.23498
H	3.15803	-1.47420	-2.49260
H	3.57288	5.15979	-3.01176
H	3.08939	4.21759	-4.42697

H	4.76577	4.20446	-3.89035
H	1.16426	-2.83204	3.49409
H	2.31067	-1.92223	4.47791
H	2.77967	-4.75821	3.39301
H	2.39352	-4.35371	5.07221
H	3.93439	-3.83771	4.36628
C	8.00044	0.42373	1.03367
H	7.79931	-0.18076	-2.37867
H	8.92483	-0.88411	-1.20499
H	8.95972	0.84618	-1.52695
H	6.00616	1.50615	-1.57545
H	7.29340	2.37799	-0.71852
H	5.85475	1.84576	0.15058
C	7.92797	-2.98984	0.61739
H	6.09006	-3.16254	-2.30143
H	7.29736	-4.28318	-1.63639
H	7.76588	-2.65338	-2.11995
H	4.61087	-3.54291	-0.23793
H	5.34158	-3.49382	1.37395
H	5.83544	-4.73224	0.21289
H	6.02415	0.01388	4.82256
H	4.84164	-1.28745	4.92193
H	4.20084	1.71115	5.12373
H	4.20153	0.56434	6.47171
H	2.99816	0.41898	5.17598
H	8.48554	-0.48923	1.37132
H	7.31813	0.75211	1.81818
H	8.76691	1.19661	0.92667
H	8.78009	-2.45092	0.20226
H	8.18540	-4.05293	0.60400
H	7.78342	-2.68887	1.64997
O	5.24842	-0.97130	-1.66199

*Table S20. Cartesian coordinates of the optimised structure of **TS2** (R = ^{i}Pr).*

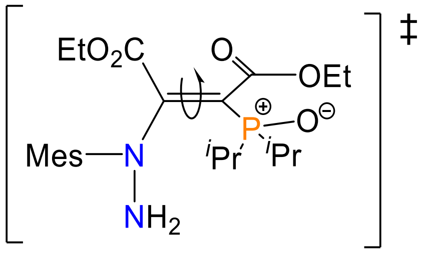

N	1.21570	0.15229	0.59142
N	2.60231	0.24116	0.43270
C	2.99479	1.27532	-0.50575
C	3.35513	2.53034	-0.03195
C	3.45704	2.85832	1.42844
C	3.65653	3.50290	-0.97794
C	3.58626	3.24927	-2.33877
C	3.17710	1.98986	-2.75850
C	2.87035	0.97625	-1.86387
C	2.45938	-0.38173	-2.34765
C	3.96800	4.30058	-3.34091
C	3.41707	-0.60411	0.97970
C	2.75241	-1.69308	1.80964
O	3.14682	-2.87774	1.41508
C	2.62698	-4.02069	2.12006
C	3.09418	-5.25113	1.38530
O	1.98358	-1.46768	2.71136
C	4.85885	-0.49236	0.91968
P	5.78810	-1.43857	-0.25276
C	7.03233	-0.31759	-1.00144
C	7.95307	-1.07878	-1.95730
C	6.35829	0.84563	-1.71977
C	6.83946	-2.70093	0.61622
C	6.89641	-3.96993	-0.23794
C	6.41772	-3.03429	2.04473
O	4.88396	-2.06342	-1.26454
C	5.50752	0.10601	2.02676
O	4.62002	0.46795	3.01329
C	5.16519	1.11281	4.15566

C	4.01785	1.44304	5.08158
O	6.70607	0.28593	2.17321
H	0.82943	1.05862	0.36835
H	1.00766	-0.12037	1.54732
H	2.87619	2.18527	2.05587
H	3.12624	3.88132	1.60813
H	4.49427	2.77594	1.75731
H	3.95603	4.48577	-0.63174
H	3.10321	1.78132	-3.81967
H	2.32136	-0.36917	-3.42767
H	1.52462	-0.70376	-1.88593
H	3.23336	-1.11621	-2.09868
H	5.00288	4.15749	-3.66114
H	3.88645	5.30239	-2.91939
H	3.33889	4.24788	-4.22999
H	1.53931	-3.94506	2.15042
H	2.99940	-3.98475	3.14528
H	2.74210	-5.24107	0.35409
H	2.70189	-6.13981	1.88120
H	4.18237	-5.31058	1.37681
H	7.60927	0.06925	-0.15711
H	7.37439	-1.57258	-2.74103
H	8.55236	-1.83458	-1.44870
H	8.64442	-0.38204	-2.43689
H	5.72044	0.48380	-2.52867
H	7.11371	1.50466	-2.15483
H	5.74567	1.43506	-1.03909
H	7.83812	-2.25921	0.66791
H	5.90514	-4.41813	-0.31861
H	7.57374	-4.69580	0.21938
H	7.23810	-3.77430	-1.25374
H	5.39821	-3.41573	2.07576
H	6.48462	-2.16719	2.69890
H	7.07676	-3.80860	2.44632
H	5.70358	2.01331	3.84816

H	5.89060	0.45344	4.63664
H	3.31651	2.12949	4.60409
H	4.39538	1.91664	5.98912
H	3.47522	0.53924	5.36104

*Table S21. Cartesian coordinates of the optimised structure of **TS2** (R = ^{t}Bu).*

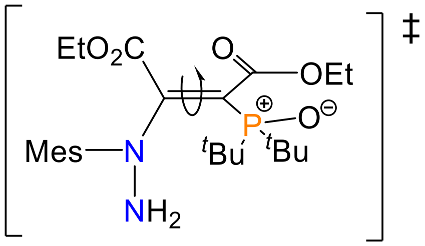

N	1.27333	-0.01276	0.69206
N	2.65681	0.16856	0.52181
C	2.99753	1.21955	-0.41354
C	3.29257	2.50091	0.03264
C	3.38233	2.85762	1.48598
C	3.54005	3.46688	-0.93578
C	3.47487	3.18253	-2.29143
C	3.13972	1.89296	-2.68448
C	2.89986	0.88319	-1.76599
C	2.60367	-0.51736	-2.21429
C	3.78940	4.23433	-3.31628
C	3.48646	-0.67799	1.04102
C	2.81086	-1.85600	1.73238
O	2.94124	-2.94443	1.01325
C	2.44432	-4.17243	1.57603
C	2.78630	-5.27370	0.60537
O	2.25536	-1.76467	2.79573
C	4.92578	-0.48386	1.12044
P	5.98241	-1.40083	0.01936
C	7.11699	-0.21110	-0.87016
C	8.08249	-1.00923	-1.75604
C	6.21262	0.61581	-1.79065
C	6.97960	-2.68106	0.97446
C	7.29163	-3.83205	0.00540

C	6.08234	-3.24044	2.08533
C	5.37056	0.08889	2.34338
O	4.31851	0.45211	3.14758
C	4.64069	0.99770	4.41856
C	3.34042	1.23255	5.15155
O	6.51692	0.24651	2.72656
H	0.81627	0.81469	0.33551
H	1.07971	-0.13317	1.68118
H	2.75204	2.22769	2.11067
H	3.10195	3.89971	1.63723
H	4.40510	2.72406	1.84219
H	3.78806	4.47158	-0.61250
H	3.08254	1.65648	-3.74073
H	2.49776	-0.55064	-3.29743
H	1.68312	-0.89801	-1.76834
H	3.41908	-1.18782	-1.91877
H	4.82674	4.14084	-3.64658
H	3.65907	5.23781	-2.91145
H	3.15414	4.13207	-4.19662
H	1.36885	-4.07326	1.73117
H	2.91625	-4.31952	2.54891
H	2.31795	-5.09631	-0.36242
H	2.43123	-6.22780	0.99691
H	3.86445	-5.33486	0.45721
C	7.88968	0.76256	0.02040
H	7.54784	-1.73874	-2.36647
H	8.84556	-1.52946	-1.17842
H	8.59880	-0.31829	-2.42829
H	5.61357	-0.02030	-2.44157
H	6.83746	1.26055	-2.41528
H	5.54756	1.25507	-1.21228
C	8.27900	-2.18251	1.61349
H	6.38332	-4.20687	-0.46491
H	7.76461	-4.64406	0.56559
H	7.97766	-3.53436	-0.78632

H	5.14790	-3.62160	1.67651
H	5.85819	-2.49212	2.84546
H	6.59937	-4.07109	2.57366
H	5.20226	1.92686	4.28858
H	5.28520	0.30373	4.96118
H	2.71857	1.95731	4.62256
H	3.54027	1.62222	6.15087
H	2.78103	0.30115	5.24431
H	8.57576	0.26789	0.70250
H	7.21636	1.37034	0.62227
H	8.47271	1.42952	-0.62277
H	9.01895	-1.89130	0.86875
H	8.71499	-3.00103	2.19454
H	8.10072	-1.34191	2.28116
O	5.12576	-2.10168	-0.98728

*Table S22. Cartesian coordinates of the optimised structure of **I3** (R = ^{i}Pr).*

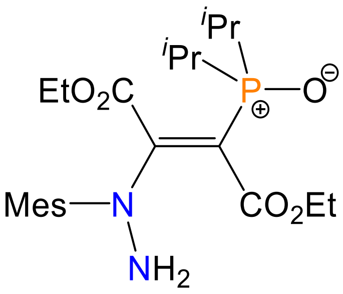

N	2.01477	2.05932	0.98191
N	2.93532	1.14595	0.44484
C	2.98085	1.04243	-0.97750
C	3.61447	2.05833	-1.70428
C	4.30579	3.18955	-1.00021
C	3.60274	1.98864	-3.08542
C	2.98696	0.93663	-3.75970
C	2.36627	-0.04824	-3.01200
C	2.33961	-0.01504	-1.61913
C	1.65785	-1.11578	-0.86002
C	3.01562	0.87802	-5.26113
C	3.53117	0.29405	1.33367

C	2.89626	0.43590	2.71213
O	3.52469	1.33866	3.44318
C	2.95686	1.63428	4.72981
C	3.42009	0.66082	5.78932
O	1.89770	-0.15915	3.00259
C	4.47400	-0.63969	1.06947
C	5.05589	-0.86748	-0.27820
O	5.81113	0.15528	-0.69831
C	6.38687	0.00768	-2.00326
C	7.23926	1.22173	-2.27134
O	4.94070	-1.88940	-0.90903
P	5.08538	-1.74021	2.37149
C	4.58231	-3.43242	1.91000
C	5.27247	-4.46012	2.80871
C	3.06099	-3.53589	2.02494
C	6.91508	-1.65956	2.31334
C	7.36593	-0.22424	2.58829
C	7.61397	-2.25132	1.09060
O	4.60338	-1.35770	3.72112
H	1.09970	1.89222	0.58153
H	2.30700	3.00447	0.77191
H	3.59291	3.94618	-0.65962
H	5.00183	3.69451	-1.66907
H	4.85746	2.82990	-0.13100
H	4.09852	2.76738	-3.65576
H	1.87826	-0.87316	-3.51905
H	0.81752	-1.50909	-1.43160
H	1.29520	-0.78989	0.11473
H	2.36121	-1.93403	-0.68975
H	2.62634	1.79976	-5.69761
H	2.42114	0.04627	-5.63778
H	4.03788	0.75191	-5.62530
H	1.87037	1.63258	4.63644
H	3.29531	2.64765	4.94312
H	3.09211	-0.34933	5.55514

H	3.00769	0.96301	6.75451
H	4.50749	0.65071	5.85576
H	5.57751	-0.09082	-2.72895
H	6.97315	-0.91263	-2.02832
H	6.63542	2.12875	-2.26648
H	7.71100	1.12828	-3.25046
H	8.02305	1.32164	-1.51955
H	4.87348	-3.58767	0.86906
H	5.06349	-4.25460	3.86039
H	6.35490	-4.47380	2.67185
H	4.89819	-5.46015	2.58023
H	2.74108	-3.36435	3.05351
H	2.73298	-4.53123	1.71815
H	2.55606	-2.80099	1.39864
H	7.17424	-2.26804	3.18694
H	7.13802	0.42179	1.73788
H	8.44493	-0.19614	2.75589
H	6.86785	0.18198	3.46869
H	7.49943	-1.61152	0.21586
H	7.24323	-3.24266	0.83007
H	8.68468	-2.33509	1.29001

Table S23. Cartesian coordinates of the optimised structure of ***I3*** *(R = ^{t}Bu).*

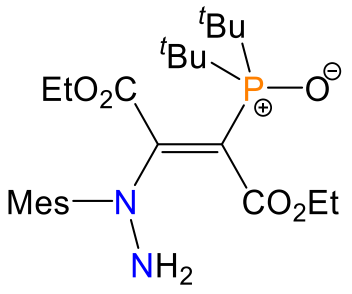

N	1.32468	1.19113	0.89102
N	2.48805	0.60432	0.37109
C	2.86459	0.97271	-0.95537
C	3.88562	1.91170	-1.12994
C	4.59718	2.51047	0.04650
C	4.22351	2.28124	-2.42432

C	3.56594	1.76045	-3.53120
C	2.52978	0.86116	-3.31975
C	2.16269	0.45361	-2.04436
C	1.04098	-0.52253	-1.84893
C	3.99011	2.13877	-4.92232
C	3.25875	-0.11065	1.24973
C	2.78560	0.10351	2.68273
O	3.24119	1.25882	3.15098
C	2.81272	1.63068	4.47014
C	3.69283	1.01762	5.53561
O	2.02409	-0.63767	3.23051
C	4.28213	-0.93478	0.91267
C	4.44935	-1.31706	-0.51858
O	5.56331	-0.82566	-1.06692
C	5.77369	-1.10101	-2.45729
C	6.97802	-0.30439	-2.89267
O	3.67673	-2.00933	-1.13102
P	5.29169	-1.69011	2.22466
C	4.91953	-3.51220	2.18868
C	5.77727	-4.20761	3.25360
C	3.44248	-3.62350	2.59725
C	7.09923	-1.31131	1.92659
C	7.19034	0.17253	1.54532
C	7.81068	-2.16141	0.87269
H	0.50722	0.74745	0.49081
H	1.31748	2.17689	0.66179
H	3.89776	2.84951	0.81200
H	5.21024	3.35556	-0.26383
H	5.24320	1.77571	0.52647
H	5.01713	3.00636	-2.57016
H	1.99934	0.45020	-4.17190
H	0.90859	-1.13945	-2.73642
H	0.09641	0.00013	-1.66908
H	1.24043	-1.18273	-1.00572
H	4.33034	3.17401	-4.96579

H	3.17482	2.01539	-5.63528
H	4.81859	1.50770	-5.25536
H	1.77160	1.33181	4.59410
H	2.87533	2.71855	4.47719
H	3.64494	-0.06839	5.49477
H	3.35945	1.36027	6.51749
H	4.73241	1.31271	5.39351
H	4.87622	-0.81492	-3.00702
H	5.92174	-2.17545	-2.58585
H	6.79476	0.76258	-2.75990
H	7.18326	-0.49229	-3.94743
H	7.86121	-0.58122	-2.31537
C	5.10761	-4.21493	0.84068
H	5.73975	-3.67931	4.20690
H	6.81862	-4.30079	2.94203
H	5.39226	-5.21854	3.41013
H	3.25799	-3.15703	3.56374
H	3.17566	-4.68206	2.65695
H	2.78702	-3.14853	1.86669
C	7.81618	-1.48526	3.27634
H	6.83979	0.34521	0.53010
H	8.23505	0.48976	1.60351
H	6.61223	0.79176	2.23415
H	7.30681	-2.13943	-0.09183
H	7.91369	-3.19854	1.19237
H	8.82001	-1.76547	0.72742
H	8.85349	-1.15963	3.15973
H	7.83083	-2.52016	3.61357
H	7.34296	-0.88370	4.05034
H	4.40539	-3.85496	0.09214
H	4.91246	-5.28168	0.98288
H	6.11804	-4.12233	0.44603
O	4.91725	-1.11991	3.54464

*Table S24. Cartesian coordinates of the optimised structure of **I4** (R = ^{i}Pr).*

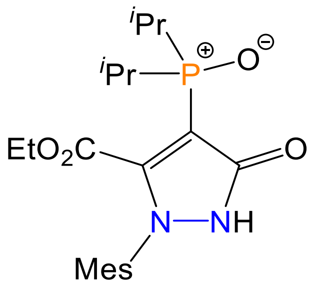

O	-2.11240	0.72427	-0.17375
C	-0.91350	0.81794	-0.08895
N	-0.24374	2.04778	-0.04141
H	-0.69010	2.86199	0.34946
N	1.09719	1.86334	0.25445
C	2.02499	2.87338	-0.17104
C	2.54230	3.72832	0.79944
C	2.14647	3.59063	2.24117
C	3.45374	4.69422	0.39695
C	3.84474	4.82161	-0.93042
C	3.30196	3.95320	-1.86825
C	2.38739	2.96948	-1.51482
C	1.82177	2.03807	-2.55020
C	4.80782	5.89706	-1.34743
C	1.31746	0.52093	0.15324
C	2.72806	0.03362	0.20994
O	3.40542	0.64100	1.17271
C	4.81645	0.37727	1.24933
C	5.40934	1.39297	2.19290
O	3.18353	-0.78983	-0.53634
C	0.16750	-0.18312	-0.02248
P	-0.10491	-1.97427	-0.17093
C	0.44890	-2.28129	-1.88964
C	0.54483	-3.76707	-2.23208
C	-0.51411	-1.56363	-2.83922
C	1.06720	-2.78914	0.99093
C	0.63839	-4.24918	1.17812
C	1.07961	-2.09460	2.35577

O	-1.50077	-2.37586	0.09589
H	1.06860	3.69842	2.37428
H	2.63821	4.34874	2.84938
H	2.41957	2.60582	2.62172
H	3.87047	5.36480	1.14043
H	3.59704	4.04208	-2.90795
H	2.06816	2.38663	-3.55183
H	0.73667	1.96668	-2.46790
H	2.22459	1.02847	-2.44118
H	5.49345	6.15140	-0.53886
H	4.26907	6.80737	-1.62149
H	5.39552	5.58971	-2.21264
H	5.23814	0.45486	0.24676
H	4.95833	-0.64658	1.59912
H	5.23635	2.40414	1.82192
H	6.48480	1.23231	2.27477
H	4.97211	1.30472	3.18781
H	1.44144	-1.82932	-1.96628
H	-0.38739	-4.28546	-1.99915
H	1.35952	-4.25718	-1.69863
H	0.73559	-3.88507	-3.30100
H	-1.52611	-1.95551	-2.72929
H	-0.19331	-1.71305	-3.87237
H	-0.55393	-0.48982	-2.65096
H	2.06474	-2.75485	0.54677
H	-0.36864	-4.29348	1.59313
H	1.32410	-4.74285	1.87025
H	0.63556	-4.81415	0.24838
H	0.06591	-2.02368	2.75593
H	1.49503	-1.08804	2.32611
H	1.67669	-2.67932	3.05888

*Table S25. Cartesian coordinates of the optimised structure of **I4** (R = ᵗBu).*

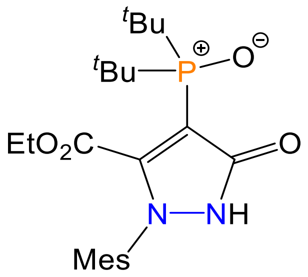

O	-1.97421	0.78739	-0.60961
C	-0.79490	0.91449	-0.39426
N	-0.17061	2.16994	-0.33563
H	-0.67759	2.96692	0.01589
N	1.13651	2.02668	0.09098
C	2.07970	3.07163	-0.17303
C	2.47374	3.87313	0.89682
C	1.93008	3.64255	2.27764
C	3.40273	4.87442	0.65174
C	3.93004	5.08551	-0.61591
C	3.50502	4.27050	-1.65763
C	2.57658	3.25676	-1.46436
C	2.14182	2.37931	-2.60235
C	4.91444	6.19322	-0.86392
C	1.40837	0.69644	0.07245
C	2.86171	0.35428	0.21525
O	3.29701	0.59883	1.44303
C	4.71571	0.47589	1.66755
C	5.42889	1.74452	1.25711
O	3.53818	-0.03986	-0.69342
C	0.29715	-0.05750	-0.16771
P	-0.08031	-1.84101	-0.13090
C	0.21604	-2.40139	-1.87407
C	0.04608	-3.92234	-1.95682
C	-0.90043	-1.73815	-2.69758
C	1.01973	-2.68480	1.12504
C	0.33115	-4.02029	1.45919
C	0.98519	-1.82768	2.40000

H	0.84018	3.69378	2.29406
H	2.31371	4.38931	2.97153
H	2.20740	2.65232	2.64200
H	3.72575	5.50367	1.47366
H	3.90713	4.42678	-2.65249
H	2.44979	2.80709	-3.55509
H	1.05896	2.25115	-2.61363
H	2.59228	1.38824	-2.51560
H	5.48189	6.42944	0.03636
H	4.39553	7.10285	-1.17586
H	5.61743	5.92870	-1.65412
H	5.08413	-0.39009	1.11904
H	4.80239	0.28944	2.73600
H	5.29456	1.93943	0.19288
H	6.49647	1.64308	1.45732
H	5.04999	2.60039	1.81574
C	1.56932	-1.98772	-2.45033
H	-0.88215	-4.24429	-1.48297
H	0.87918	-4.45396	-1.49482
H	0.01126	-4.22028	-3.00797
H	-1.88506	-1.97409	-2.29669
H	-0.83670	-2.09842	-3.72793
H	-0.80162	-0.65200	-2.71524
C	2.46251	-2.97632	0.71232
H	-0.70043	-3.86164	1.76682
H	0.87869	-4.49531	2.27794
H	0.33137	-4.71011	0.61654
H	-0.04160	-1.57788	2.67152
H	1.55053	-0.90247	2.30608
H	1.42266	-2.40079	3.22198
H	1.62196	-2.31622	-3.49209
H	2.41222	-2.42977	-1.92185
H	1.69935	-0.90614	-2.43677
H	2.50782	-3.71198	-0.09116
H	2.99366	-3.39957	1.56976

H	3.01376	-2.09890	0.38410
O	-1.48504	-2.06557	0.27433

Table S26. Cartesian coordinates of the optimised structure of ***3****.*

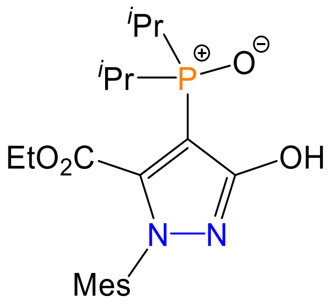

O	4.57228	-0.29136	-0.31625
C	4.42673	0.21822	0.89627
N	3.95945	1.44431	1.06105
N	3.95522	1.64672	2.39010
C	3.41442	2.87269	2.89087
C	4.24221	3.98600	2.96961
C	5.67769	3.90252	2.54197
C	3.70112	5.15721	3.47852
C	2.37772	5.22950	3.89758
C	1.58602	4.09332	3.79592
C	2.08509	2.89964	3.29108
C	1.22940	1.66865	3.20630
C	4.40263	0.56827	3.07004
C	4.51072	0.46683	4.53565
O	4.37280	1.63738	5.14031
C	4.36580	1.66108	6.57314
C	4.05811	3.08047	6.98201
O	4.70836	-0.57605	5.11019
C	4.72194	-0.40316	2.13478
P	5.37790	-2.06545	2.20004
C	6.97446	-1.94571	3.07225
C	7.59161	-3.31739	3.34505
C	7.92276	-1.06174	2.26049
C	4.25337	-3.12695	3.17939
C	4.39940	-4.58189	2.72297

C	2.79957	-2.67059	3.05174
H	6.22128	3.17920	3.15456
H	6.16907	4.86948	2.63989
H	5.75672	3.57409	1.50469
H	4.33070	6.03734	3.55195
C	1.80951	6.52053	4.41705
H	0.55010	4.13581	4.11435
H	0.17944	1.91636	3.35580
H	1.51250	0.94091	3.97170
H	1.33560	1.17998	2.23694
H	5.33914	1.32684	6.93636
H	3.61349	0.95673	6.93182
H	4.82688	3.76291	6.61887
H	4.01513	3.15083	8.06946
H	3.09970	3.39838	6.56954
H	6.74586	-1.46125	4.02540
H	7.70948	-3.88606	2.42049
H	6.99162	-3.90505	4.04029
H	8.58119	-3.19158	3.78869
H	8.13091	-1.51221	1.28873
H	8.86816	-0.94584	2.79395
H	7.50669	-0.06785	2.08910
H	4.56323	-3.02532	4.22192
H	4.10093	-4.68461	1.67949
H	3.75606	-5.21907	3.33304
H	5.41995	-4.95230	2.80935
H	2.48872	-2.63467	2.00510
H	2.64432	-1.68537	3.48881
H	2.14836	-3.37777	3.56931
H	4.93918	-1.19823	-0.19579
O	5.51040	-2.53056	0.78179
H	2.55730	7.08689	4.97338
H	0.95600	6.34425	5.07206
H	1.46824	7.14982	3.59145

Table S27. Cartesian coordinates of the optimised structure of hypothetical pyrazolyl-phosphine oxide, R = ᵗBu (structure below).

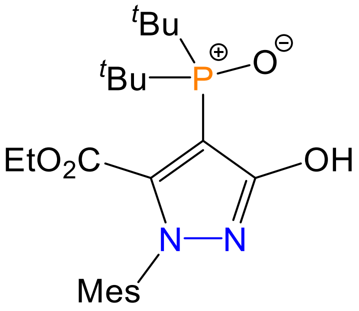

O	4.07568	-0.54283	-0.09503
C	4.10947	0.07811	1.07097
N	3.59827	1.28805	1.19234
N	3.76367	1.60904	2.48868
C	3.40456	2.91892	2.93307
C	4.40386	3.87010	3.09631
C	5.84079	3.55206	2.78943
C	4.02964	5.12812	3.55577
C	2.70597	5.44113	3.82954
C	1.73780	4.46054	3.63942
C	2.06451	3.18942	3.19428
C	1.02372	2.12331	3.02490
C	4.38903	0.63812	3.18182
C	4.71104	0.81556	4.61564
O	3.69090	1.34893	5.27504
C	3.92378	1.79145	6.62094
C	2.84539	2.79209	6.95414
O	5.76992	0.53441	5.11364
C	4.64926	-0.40303	2.30302
P	5.31196	-2.07192	2.38042
C	7.09587	-2.06857	2.90673
C	7.75265	-3.28152	2.22708
C	7.71964	-0.78537	2.33793
C	4.19287	-3.04774	3.48382
C	4.72603	-4.48199	3.58941
C	2.83668	-3.08016	2.76089
H	5.92830	2.99426	1.85618

H	6.29506	2.94503	3.57643
H	6.42395	4.46752	2.69962
H	4.79463	5.88492	3.69020
C	2.31688	6.81794	4.29052
H	0.69960	4.69063	3.85277
H	0.03637	2.49941	3.28924
H	1.24951	1.26598	3.66321
H	0.99875	1.76415	1.99525
H	4.92014	2.23033	6.67749
H	3.90014	0.92044	7.27851
H	2.88911	3.63996	6.26912
H	2.98417	3.15521	7.97325
H	1.85614	2.33942	6.88035
C	7.36068	-2.13622	4.41167
H	7.61956	-3.25629	1.14748
H	7.35144	-4.22638	2.59471
H	8.82174	-3.26321	2.45314
H	7.50719	-0.67114	1.27318
H	8.80428	-0.83763	2.46050
H	7.35946	0.09859	2.86404
C	3.99431	-2.44157	4.87420
H	4.92503	-4.90231	2.60267
H	3.97314	-5.10648	4.07666
H	5.63764	-4.54202	4.18385
H	2.92338	-3.53478	1.77490
H	2.42016	-2.07825	2.64095
H	2.13408	-3.66816	3.35675
H	4.49487	-1.42688	0.04893
O	5.22611	-2.60944	0.98137
H	3.16284	7.34275	4.73454
H	1.51414	6.77605	5.02792
H	1.95853	7.41731	3.45013
H	3.47818	-1.48223	4.81783
H	4.92497	-2.30372	5.42079
H	3.35872	-3.11115	5.45963

H	6.91451	-1.30235	4.94677
H	8.44162	-2.08915	4.56912
H	7.01384	-3.07300	4.84802

S6. References

S1. E. D. E. Calder, L. Male and A. R. Jupp, *Dalton Trans.*, 2024, **53**, 15032-15039

S2. CrysAlisPro, Rigaku Oxford Diffraction, 2021.

S3. O. V. Dolomanov, L. J. Bourhis, R. J. Gildea, J. A. K. Howard and H. Puschmann, *J. Appl. Crystallogr.*, 2009, **42**, 339-341.

S4. G. M. Sheldrick, *Acta Crystallogr.*, 2015, **A71**, 3-8

S5. G. M. Sheldrick, *Acta Crystallogr.*, 2015, **C71**, 3-8.

S6. E.J. Jordan, E.D.E. Calder, H.V. Adcock, L. Male, M. Nieger, J.C. Slootweg, and A.R. Jupp, *Chem. - Eur. J.*, 2024, **30**, e202401358.

S7. Gaussian 16, Revision C.02, M. J. Frisch, G. W. Trucks, H. B. Schlegel, G. E. Scuseria, M. A. Robb, J. R. Cheeseman, G. Scalmani, V. Barone, G. A. Petersson, H. Nakatsuji, X. Li, M. Caricato, A. V. Marenich, J. Bloino, B. G. Janesko, R. Gomperts, B. Mennucci, H. P. Hratchian, J. V. Ortiz, A. F. Izmaylov, J. L. Sonnenberg, D. Williams-Young, F. Ding, F. Lipparini, F. Egidi, J. Goings, B. Peng, A. Petrone, T. Henderson, D. Ranasinghe, V. G. Zakrzewski, J. Gao, N. Rega, G. Zheng, W. Liang, M. Hada, M. Ehara, K. Toyota, R. Fukuda, J. Hasegawa, M. Ishida, T. Nakajima, Y. Honda, O. Kitao, H. Nakai, T. Vreven, K. Throssell, J. A. Montgomery, Jr., J. E. Peralta, F. Ogliaro, M. J. Bearpark, J. J. Heyd, E. N. Brothers, K. N. Kudin, V. N. Staroverov, T. A. Keith, R. Kobayashi, J. Normand, K. Raghavachari, A. P. Rendell, J. C. Burant, S. S. Iyengar, J. Tomasi, M. Cossi, J. M. Millam, M. Klene, C. Adamo, R. Cammi, J. W. Ochterski, R. L. Martin, K. Morokuma, O. Farkas, J. B. Foresman, and D. J. Fox, Gaussian, Inc., Wallingford CT, 2016.

S8. J-D. Chai and M. Head-Gordon, *Phys. Chem. Chem. Phys.*, 2008, **10**, 6615-6620.

S9. F. Weigend and R. Ahlrichs, *Phys. Chem. Chem. Phys*., 2005, **7**, 3297-3305.

S10. M. Nottoli and F. Lipparini, *J. Chem. Phys.*, 2020, **153**, 224108.

S11. NBO Version 3.1, E. D. Glendening, A. E. Reed, J. E. Carpenter, and F. Weinhold.
